# Supplementary material for: Systematic examination of preprint platforms for use in the medical and biomedical sciences setting
Source: BMJ Open. 2020 Dec 29;10(12):e041849. doi: 10.1136/bmjopen-2020-041849 (PMC7778769; doi:10.1136/bmjopen-2020-041849)
Supplement: Supplementary data [file bmjopen-2020-041849supp001.pdf]

Supplementary Table 1: Reasons for excluded preprint platforms

Name of Site	Reason for Exclusion
Chemweb	Inactive
Centre for Health Economics and Policy Analysis (CHEPA)	Inactive
ClinMed NetPrints	Inactive
Cogprints	Inactive
CSTC	Inactive
K-Theory Preprint Archives	Inactive
Mathematics Preprint Search System (MPRESS)	Inactive
National Advisory Committee for Aeronautics (NACA)	Inactive
Nature Precedings	Inactive
The Winnower	Inactive
Geometry Center's Preprints	Inactive
World Health Organisation Zika Open repository	Inactive
Instant Math Preprints (Yale Mathematics Preprint Bulletin Board)	Inactive
Organisation Européenne pour la Recherche Nucléaire document platform	Repository
Hyper Articles en Ligne (HAL)	Repository
ResearchGate	Repository
White Rose Consortium e-Prints Repository	Repository
Zenodo	Repository
Organisation Européenne pour la Recherche Nucléaire Print	Offline
Fermilab	Offline
Physics Information Exchange (PIE)	Offline
Radio Astronomy Preprints – RAPsheet	Offline
Space Telescope Preprints – STEPsheet	Offline
Advance: a SAGE preprints community	Scope: Humanities and Social Sciences
BodoArXiv	Scope: Medieval Studies
Cryptology ePrint Archive	Scope: Cryptology
CORE repository	Scope: Humanities
EarthArXiv	Scope: Earth Sciences
EconStor	Scope: Economics and Business Studies
ECSarXiv	Scope: Electrochemistry and solid state science
engrXiv	Scope: Engineering
E-LIS	Scope: Library and Information Science
Electronic Colloquium on Computational Complexity	Scope: Computer Science
Institute for Fiscal Studies (IFS) Working Papers	Scope: Economics
LawArxiv	Scope: Law
LIS Scholarship Archive	Scope: Library and Information Science
LingBuzz	Scope: Linguistics
MediArXiv	Scope: Media, Film and Communication Studies
Mathematical Physics Preprint Archive (mp_arc)	Scope: Mathematical Physics and Related Areas
National Bureau of Economic Research (NBER) Working Papers	Scope: Economics
Networked Computer Science Technical Reference Library (NCSTRL)	Scope: Computer Science
Philsci Archive	Scope: Philosophy of Science
Munich Personal RePEc Archive (MPRA)	Scope: Economics
Social Science Open Access Repository (SSOAR)	Scope: Social Sciences
Stanford Physics Information Retrieval System (SPIRES)	Scope: Physics
WorldBank's Policy Research Working Paper Series (PRWPs)	Scope: Economics
