# Supplementary material for: Systematic examination of preprint platforms for use in the medical and biomedical sciences setting
Source: BMJ Open. 2020 Dec 29;10(12):e041849. doi: 10.1136/bmjopen-2020-041849 (PMC7778769; doi:10.1136/bmjopen-2020-041849)
Supplement: Supplementary data [file bmjopen-2020-041849supp002.pdf]

Supplementary Table 2: Scope and ownership of preprint platforms

Preprint Server (date launched/content first posted; number of articles[a])	Platform Description	Scope	Ownership (O)/Ownership Type (OT)/For Profit status (P)/Technology (T; name & openness of source code)	Time to publication (T) / Processing Charges (C)
**OSF Communities**				
AfricArxiv [1]<br>(22 June 2018; 103)<br>*Verified*	"..is a free, open source and community-led digital archive for African research"	All scientific fields	O[b]: Small group of enthusiasts<br>OT: Academic community group; charity<br>P: Non-profit or not-for-profit<br>T: Open Science Framework (open source)	T: A few days<br>C: No fee to author
AgriXiv [2]<br>(15 February 2017; 418)<br>*Verified*	"Preprints for Agriculture and Allied Sciences"	Relating to agriculture and allied sciences, including life sciences, medicine and health sciences, social and behavioural sciences	O[b]: Open Access India and small group of enthusiasts<br>OT: Academic community group<br>P: Non-profit or not-for-profit<br>T: Open Science Framework (open source)	T: Unknown<br>C: No fee to author
Arabixiv [3]<br>(2 July 2018; 424)<br>*Verified*	"The Arabic Preprint Server"	All scientific fields	O[b]: Small group of enthusiasts<br>OT: Academic community group<br>P: Non-profit or not-for-profit<br>T: Open Science Framework (open source)	T: Up to 48 hours<br>C: No fee to author
EcoEvoRxiv [4]<br>(21 March 2018; 191)<br>*Verified*	"A free preprint service for ecology, evolution and conservation"	Subject-specific, including ecology, evolution and conservation	O[b]: Transparency in Ecology and Evolution<br>OT: Academic community group<br>P: Non-profit or not-for-profit<br>T: Open Science Framework (open source)	T: Up to 48 hours<br>C: No fee to author
FocUS Archive [5]<br>(20 September 2017; 42)<br>*Verified*	"A free preprint service for the focused ultrasound research community"	Relating to ultrasound, including life sciences, medicine and health sciences	O[b]: Focused Ultrasound Foundation<br>OT: Funding organisation (funder); medical research organisation<br>P: Non-profit or not-for-profit<br>T: Open Science Framework (open source)	T: Unknown<br>C: No fee to author
Frenxiv [6]<br>(06 July 2018; 94)<br>*Verified*	"The French server for Preprints in all the scientific fields"	All scientific fields	O[b]: Small group of enthusiasts<br>OT: Individual or community<br>P: Non-profit or not-for-profit<br>T: Open Science Framework (open source)	T: A few days<br>C: No fee to author
INA-Rxiv [7]<br>(17 August 2017; 16,637)<br>*Verified*	"A preprint server for Indonesian academia to provide an open, free and sustainable scientific repository"	All scientific fields	O[b]: Indonesia open science team<br>OT: Academic community group<br>P: Non-profit or not-for-profit<br>T: Open Science Framework (open source)	T: Unknown<br>C: No fee to author
MarXiv [8]<br>(09 November 2017; 449)<br>*note: no longer on the OSF*<br>*Verified*	"The free research repository for the ocean and marine-climate sciences"	Relating to ocean and marine climate sciences, including life sciences, social and behavioural sciences	O[b]: Open Communications for the Ocean (OCTO group)<br>OT: Publishing organisation<br>P: For-profit<br>T: Open Science Framework (open source)	T: Up to 48 hours<br>C: No fee to author
MetaArXiv [9]<br>(17 March 2017; 121)<br>*Verified*	"An interdisciplinary archive of articles focused on improving research transparency and reproducibility"	Relating to meta-science	O[b]: The Berkeley Initiative for Transparency in the Social Sciences (BITSS), Centre for Effective Global Action, University of California, Berkeley<br>OT: Academic institution	T: Unknown<br>C: No fee to author

Kirkham JJ, *et al. BMJ Open* 2020; 10:e041849. doi: 10.1136/bmjopen-2020-041849

			P: Non-profit or not-for-profit<br>T: Open Science Framework (open source)	
MindRxiv [10]<br>(17 July 2017; 192)<br>*Verified*	"Open archive for research on mind and contemplative practices"	Relating to mind and contemplative practices, including medicine and health sciences, neuroscience and neurobiology, psychology, social and behavioural sciences	O[b]: Mind and Life Institute<br>OT: Academic institution<br>P: Non-profit or not-for-profit<br>T: Open Science Framework (open source)	T: Same day<br>C: No fee to author
NutriXiv [11]<br>(11 September 2017; 63)<br>*Verified*	"A free preprint service for the nutritional sciences"	Relating to nutritional sciences	O[b]: Small group of enthusiasts<br>OT: Academic Institution<br>P: Non-profit or not-for-profit<br>T: Open Science Framework (open source)	T: Unknown<br>C: No fee to author
OSF Preprints [12]<br>(23 May 2007; 15,174)<br>*Verified*	"OSF Preprints is a preprints discovery site, where you can search not only the preprints in all subjects hosted at OSF Preprints, but also preprints on partner sites such as arXiv, AgriXiv, engrXiv, PsyArXiv and SocArXiv"	All scientific fields	O[b]: Center for Open Science<br>OT: Charity<br>P: Non-profit or not-for-profit<br>T: Open Science Framework (open source)	T: Unknown<br>C: No fee to author
PaleorXiv [13]<br>(18 August 2017; 162)<br>*Verified*	"…is a free, open source and community-led digital archive for Paleontology research"	Relating to palaeontology and paleobiology research	O[b]: Small group of enthusiasts<br>OT: Academic community group<br>P: Non-profit or not-for-profit<br>T: Open Science Framework (open source)	T: A few days<br>C: No fee to author
PsyArXiv [14]<br>(09 December 2016; 8,509)<br>*Verified*	"…is designed to facilitate rapid dissemination of psychological research"	Relating to psychology and psychological sciences	O[b]: Society for the Improvement of Psychological Science (SIPS)<br>OT: Scientific society<br>P: Non-profit or not-for-profit<br>T: Open Science Framework (open source)	T: A few days<br>C: No fee to author
SocArxiv [15]<br>(16 June 2017; 5,149)<br>*Verified*	"…open archive of the social sciences"	Relating to social sciences including social and behavioural sciences	O[b]: University of Maryland, College Park (UMD)<br>OT: Academic institution<br>P: Non-profit or not-for-profit<br>T: Open Science Framework (open source)	T: Unknown<br>C: No fee to author
SportRxiv [16]<br>(01 April 2017; 173)<br>*Verified*	"… is the first community-led and open access subject repository dedicated to sport, exercise, performance, and health research"	Relating to sports and exercise science, including rehabilitation and therapy, theatre, dance, physiology, physiotherapy, psychology, sociology	O[b]: Society for Transparency, Openness, and Replication in Kinesiology (STORK)<br>OT: Scientific society<br>P: Non-profit or not-for-profit<br>T: Open Science Framework (open source)	T: A few days<br>C: No fee to author
Thesis Commons [17]<br>(21 April 2017; 583)<br>*Verified*	"An open archive of theses"	All scientific fields	O[b]: Center for Open Science and small group of enthusiasts<br>OT: Academic community group; charity<br>P: Non-profit or not-for-profit<br>T: Open Science Framework (open source)	T: Unknown<br>C: No fee to author
Open Research Central infrastructure				
AAS Open Research [18]<br>(18 April 2018; 61)<br>*Verified*	"…is a platform for rapid publication and open peer review for researchers supported by AAS (African Academy of Sciences) and programs supported through its funding platform, AESA (Accelerating Excellence in	Multiple scientific fields, including health and wellbeing[c]	O: African Academy of Sciences<br>OT: Funding organisation (funder)<br>P: Non-profit or not-for-profit<br>T: Custom F1000 software (closed source)	T: Up to 1 week<br>C: No fee to author (APC between £120-£800 typically paid by AAS)

	Science in Africa's)"			
AMRC Open Research [19] (19 February 2019; 7) *Ve*	"A platform for rapid author-led publication and open peer review of research funded by AMRC (Association of Medical Research Charities) member charities"	Broad life & biomedical research, including basic scientific, translational, applied and clinical research	O: Association of Medical Research Charities<br>OT: Funding organisation (funder), membership organisation<br>P: Non-profit or not-for-profit<br>T: Custom F1000 software (closed source)	T: Up to 1 week<br>C: Author fee applies (min £120; max £800[d]; £20 per poster, slide or document)
Gates Open Research [20] (06 November 2017; 218) *Verified*	"…is a platform for rapid author-led publication and open peer review of research funded by the Bill & Melinda Gates Foundation"	Global health, global development, global growth and opportunity	O: Bill and Melinda Gates Foundation<br>OT: Funding organisation (funder)<br>P: Non-profit or not-for-profit<br>T: Custom F1000 software (closed source)	T: Up to 1 week<br>C: No fee to author (APC between $150-$1500[d] paid by the Gates Foundation)
HRB Open Research [21] (28 February 2018; 80) *Verified*	"…is a platform for HRB-funded (Health Research Board) researchers to rapidly publish their research outputs in an open and accessible way"	Broad life & biomedical research, including exploratory, translational, biomedical, clinical, epidemiological, health services research	O: Health Research Board Ireland<br>OT: Funding organisation (funder)<br>P: Non-profit or not-for-profit<br>T: Custom F1000 software (closed source)	T: Up to 1 week<br>C: No fee to author (APC between €123-€818[d] paid by HRB)
MNI Open Research [22] (05 December 2017; 11) *Verified*	"…is a platform for rapid publication and open peer review of research conducted by MNI (Montreal Neurological Institute) and Hospital researchers"	Subject-specific, including brain imaging, cellular and molecular neuroscience, cognitive neuroscience, neurological diseases/disorders	O: Montreal Neurological Institute and Hospital, McGill University<br>OT: Academic institution<br>P: Non-profit or not-for-profit<br>T: Custom F1000 software (closed source)	T: Up to 1 week<br>C: No fee to author (APC between £116-£775[e] paid by MNI)
Wellcome Open Research [23] (15 November 2016; 602) *Verified*	"A new way for Wellcome-funded researchers to rapidly publish any results they think are worth sharing"	Broad life & biomedical research, including basic scientific, translational and clinical research	O: Wellcome Trust<br>OT: Funding organisation (funder)<br>P: Non-profit or not-for-profit<br>T: Custom F1000 software (closed source)	T: Up to 1 week<br>C: No fee to author (APC between £116-£775[d] paid by Wellcome)
**Others**				
arXiv [24] (14 August 1991; 1,688,374) *Verified*	"Open access to…e-prints…"	Multiple scientific fields, including quantitative biology[c]	O: Cornell University<br>OT: Academic institution<br>P: Non-profit or not-for-profit<br>T: SWORD (open source)	T: A few days<br>C: No fee to author
Authorea [25] (06 August 2013; 2,579) *Verified*	"…is the leading collaborative platform to read, write, and publish research"	All scientific fields	O: Atypon (Wiley)<br>OT: Publishing organisation (publisher)<br>P: For-profit<br>T: Custom Authorea software (proprietary software with some open source components)	T: Same day<br>C: No fee to author
bioRxiv [26] (7 November 2013; 79,370) *Verified*	"…is a free online archive and distribution service for unpublished preprints in the life sciences…"	Broad life & biomedical research (from animal behaviour and cognition to zoology)	O: Cold Spring Harbor Laboratory (CSHL)<br>OT: Academic institution<br>P: Non-profit or not-for-profit<br>T: Highwire Preprint & BenchPress (proprietary software with some open source components)	T: Up to 48 hours<br>C: No fee to author
Cell Press Sneak Peek [27] (02 April 2018; 2,152) *Verified*	"…is an author opt-in preview of the papers under review in [Cell Press] primary research journals"	Subject-specific, including cancer; stem cell; neuron; cell development; biology; immunity	O: Elsevier<br>OT: Publishing organisation (publisher)<br>P: For-profit<br>T: Unknown	T: A few days<br>C: No fee to author
ChemRxiv [28] (15 August 2017; 4,332)	"…is a free submission, distribution and archive service for unpublished preprints in	Subject-specific, including biological and medicinal chemistry	O: American Chemical Society, German Chemical Society, and Royal Society of Chemistry	T: Up to 48 hours<br>C: No fee to author

	chemistry and related areas"		OT: Scientific society<br>P: Non-profit or not-for-profit<br>T: Figshare (closed source)	
ChinaXiv [29]<br>(25 January 2016; 13,561)	"...is an open repository of Chinese scientific research papers in the field of natural sciences"	All scientific fields	O: Chinese Academy of Sciences<br>OT: Academic institution<br>P: Non-profit or not-for-profit<br>T: Java, SQL server (closed source)	T: Unknown<br>C: No fee to author
Earth and Space Science Open Archive (ESSOAr) [30]<br>(11 February 2018; 671)<br>*Verified*	"...is a community server established to accelerate the open discovery and dissemination of Earth and space science"	Relating to earth, environmental, and space sciences (subject-specific topics include biology, microbiology and public health)	O: American Geophysical Union (AGU), Wiley<br>OT: Scientific society; publishing organisation (publisher)<br>P: Non-profit or not-for-profit<br>T: Atypon/Literatum (closed source)	T: A few days<br>C: No fee to author
F1000 Research [31]<br>(13 July 2012; 3,226) – Verified	"...is an Open Research publishing platform for life scientists, offering immediate publication of articles and other research outputs without editorial bias"	Broad life & biomedical research, including basic scientific, translational and clinical research within the life sciences and medicine	O: F1000 Research Ltd.<br>OT: Publishing organisation (publisher)<br>P: For-profit<br>T: Custom F1000 software (closed source)	T: Up to 1 week<br>C: Author fee applies (min $150; max $2000 US)[d]
JMIR Preprints [32]<br>(11 November 2015; Not available)	"A preprint server for pre-publication/pre-peer-review preprints intended for community review as well as ahead-of-print (accepted) manuscripts"	Broad life & biomedical research	O: JMIR Publications Inc.<br>OT: Publishing organisation (publisher)<br>P: Unknown<br>T: Unknown	T: Unknown<br>C: No fee to author
medRxiv [33]<br>(25 June 2019; 3,412)<br>*Verified*	"...is a free online archive and distribution server for complete but unpublished manuscripts (preprints) in the medical, clinical, and related health sciences"	Broad medical, clinical and related health sciences	O: Cold Spring Harbor Laboratory (CSHL), Yale University and BMJ<br>OT: Academic institution<br>P: Non-profit or not-for-profit<br>T: Highwire Preprint & BenchPress (proprietary software with some open source components)	T: A few days<br>C: No fee to author
MitoFit Preprint Archives [34]<br>(12 February 2019; 15)	"Open Access preprint server for mitochondrial physiology and bioenergetics"	Subject-specific, including mitochondrial physiology, bioenergetics, ergodynamics	O: Oroborus Instruments<br>OT: Life sciences tech company<br>P: Non-profit or not-for-profit<br>T: MediaWiki (open source)	T: Up to 1 week<br>C: No fee to author
NeuroImage: Clinical - *First Look* [35]<br>(01 August 2018; 12)<br>*Verified*	"...is an author opt-in preview of the papers currently under consideration in [NeuroImage: Clinical]"	Subject-specific, including diseases, disorders and syndromes involving the nervous system, neuroimaging	O: Elsevier<br>OT: Publishing organisation (publisher)<br>P: For-profit<br>T: Unknown	T: A few days<br>C: No fee to author
PeerJ Preprints [36]<br>(04 April 2013, 5,068; *note: no new preprints accepted after 30 September 2019*)<br>*Verified*	"...is the 'pre-print' area of PeerJ"	Biological, medical and environmental sciences, matching scope to *PeerJ* journal (excludes Clinical Trials, diagnostic, therapeutic or health implications)	O: PeerJ<br>OT: Publishing organisation (publisher)<br>P: For-profit<br>T: Custom PeerJ Preprints software (closed source)	T: Up to 24 hours<br>C: No fee to author
Preprints with The Lancet [37]<br>(30 June 2018; 5,073)<br>*Verified*	"...a place where journals and other research experts identify content of interest prior to publication...Authors have either opted in at submission to The Lancet family of journals to post their preprints on Preprints with The	Medical sciences	O: Elsevier<br>OT: Publishing organisation (publisher)<br>P: For-profit<br>T: Unknown	T: Up to 2 weeks<br>C: No fee to author

	Lancet, or submitted directly via SSRN"			
Preprints.org [38] (02 July 2016; 14,837)	"...is a multidisciplinary preprint platform that makes scientific manuscripts from all fields of research immediately available"	All scientific fields	O: Multidisciplinary Digital Publishing Institute (MDPI)<br>OT: Publishing organisation (publisher)<br>P: Non-profit or not-for-profit<br>T: Custom software (closed source)	T: Up to 24 hours<br>C: No fee to author
Research Square [39] (15 October 2018; 20,917) *Verified*	"...lets you share your work early, gain feedback from the community, and start making changes to your paper prior to peer review in a journal"	All scientific fields	O: Research Square<br>OT: Publishing services organisation<br>P: For-profit<br>T: Custom software (closed source)	T: A few days<br>C: No fee to author
SciELO Preprints [40] (06 July 2018; 31) *Verified*	"…focus on manuscripts related to the main topics of the SciELO (Scientific Electronic Library Online) 20 Years Week agenda"	Multiple scientific fields including biological, health, human, applied social sciences[c]	O: SciELO and FAPESP Program<br>OT: Research infrastructure program<br>P: Non-profit or not-for-profit<br>T: Open Journals System (open source)	T: A few days<br>C: No fee to author
SSRN [41] (1994; 791,714) *Verified*	"…is an open-access online preprint community providing valuable services to leading academic schools and government institutions…"	Multiple scientific fields including applied sciences, health sciences, life sciences, social sciences[c]	O: Elsevier<br>OT: Publishing organisation (publisher)<br>P: For-profit<br>T: Unknown	T: Up to 48 hours<br>C: No fee to author
Surgery Open Science - *First Look* [42] (23 April 2019; 42) *Verified*	"…open access journal affiliated with the journal *Surgery* and striving to incorporate the strongest tenets of the open science movement including allowing author preprint"	Relating to surgery, matching scope to *Surgery* journal	O: Elsevier<br>OT: Publishing organisation (publisher)<br>P: Unknown<br>T: Unknown	T: A few days<br>C: No fee to author
Therapoid [43] (08 February 2017; 9) *Verified*	"an open web platform for scientific collaboration by Open Therapeutics"	Relating to pharmaceutical, medical device, and medical/health diagnostic technologies	O: Open Therapeutics<br>OT: Life sciences tech company<br>P: For-profit<br>T: Custom software (proprietary software with some open source components)	T: Same day<br>C: No fee to author
ViXra [44] (25 February 2007; 35,405)	"An alternative archive… in Science and Mathematics serving the whole scientific community"	Multiple scientific fields, including biology[c]	O: Small group of enthusiasts<br>OT: Individual or community<br>P: Non-profit or not-for-profit<br>T: Custom viXra software with services from formsite.com and disqus.com (closed source)	T: Up to 48 hours<br>C: No fee to author

APC: Article Processing Charge

[a] As of 21st April 2020 (accept for SSRN, last verified 11th September 2019); [b] OSF communities are still working out the balance of ownership between service leads and the Centre for Open Science; [c] Disciplinary scope of server is broader – we list only those relevant to biomedical and health sciences; [d] Based on article length, surcharges may apply for articles with unusually large word counts; [e] MNI will pay for one publication per lab only.

Preprint platform websites

1. AfricArxiv https://info.africarxiv.org/
2. AgriXiv https://agrixiv.org
3. Arabixiv https://arabixiv.org/
4. EcoEvoRxiv https://ecoevorxiv.org
5. FocUS Archive https://osf.io/preprints/focusarchive/
6. Frenxiv https://frenxiv.org
7. INA-Rxiv https://osf.io/preprints/inarxiv
8. MarXiv https://marxiv.org
9. MetaArXiv https://osf.io/preprints/metaarxiv/
10. MindRxiv https://mindrxiv.org
11. NutriXiv https://osf.io/preprints/nutrixiv
12. OSF Preprints https://osf.io/preprints/
13. PaleorXiv https://paleorxiv.org
14. PsyArXiv https://psyarxiv.com
15. SocArXiv https://osf.io/preprints/socarxiv
16. SportRxiv https://osf.io/preprints/sportrxiv
17. Thesis Commons https://thesiscommons.org
18. AAS Open Research https://aasopenresearch.org/
19. AMRC Open Research https://amrcopenresearch.org/
20. Gates Open Research https://gatesopenresearch.org/
21. HRB Open Research https://hrbopenresearch.org/
22. MNI Open Research https://mniopenresearch.org/
23. Wellcome Open Research https://wellcomeopenresearch.org/
24. arXiv https://arxiv.org
25. Authorea https://www.authorea.com
26. bioRxiv https://www.biorxiv.org/
27. Cell Press Sneak Peek https://papers.ssrn.com/sol3/Jeljour_results.cfm?form_name=journalBrowse&journal_id=3184889
28. ChemRxiv https://chemrxiv.org
29. ChinaXiv http://chinaxiv.org
30. ESSOAr https://www.essoar.org
31. F1000 Research https://www.essoar.org
32. JMIR Preprints https://preprints.jmir.org/
33. medRxiv https://www.medrxiv.org
34. MitoFit Preprint Archives https://www.mitofit.org/index.php/MitoFit_Preprint_Archives
35. NeuroImage: Clinical – *First Look* https://papers.ssrn.com/sol3/JELJOUR_Results.cfm?form_name=journalBrowse&journal_id=3178959
36. PeerJ Preprints https://peerj.com/preprints/
37. Preprints with The Lancet https://papers.ssrn.com/sol3/JELJOUR_Results.cfm?form_name=journalBrowse&journal_id=3184962
38. Preprints.org https://www.preprints.org/
39. Research Square https://www.researchsquare.com
40. SciELO Preprints https://preprints.scielo.org/index.php/scielo
41. SSRN https://www.ssrn.com
42. Surgery Open Science – *First Look* https://papers.ssrn.com/sol3/Jeljour_results.cfm?form_name=journalBrowse&journal_id=3303309
43. Therapoid https://therapoid.net
44. ViXra http://vixra.org
