# Supplementary material for: Systematic examination of preprint platforms for use in the medical and biomedical sciences setting
Source: BMJ Open. 2020 Dec 29;10(12):e041849. doi: 10.1136/bmjopen-2020-041849 (PMC7778769; doi:10.1136/bmjopen-2020-041849)
Supplement: Supplementary data [file bmjopen-2020-041849supp003.pdf]

Supplementary Table 3: Submission, journal transfer options and external discoverability

Preprint Server	Major content accepted (level of research[a], where known)	Content Language(s)	Permitted submission formats (S)<br>Viewing options (V)<br>Unique identifier (I; if DOI, prefix in brackets)<br>Versioning policy / version identifiers (VP)<br>Licensing options (L)<br>Reader registration required to view or download full content (R)	Journal submission options<br>Preprint displays link to journal publication (PL)	External content indexing (EI) /<br>Metadata availability (MA) /<br>Machine-readable full-text content (MR)
**OSF Communities**					
AfricArxiv [1] – *Verified*	Any scholarly content	Afrikaans, Akan, English, French, Igbo, Swahili, Zulu, other unspecified native African languages – summary must be provided in English and French, non-English languages held in moderation queue awaiting verification	S: PDF, Word doc, other text formats<br>V: By download in file format submitted by author(s), PDF viewable in browser without download<br>I: DOI (10.31730/osf.io)<br>VP: Accepts any new versions / single DOI retained for all versions<br>L: Authors are provided with a choice of licenses: GNU Lesser General Public License (LGPL) 3.0, BSD 3-Clause "New"/"Revised" License, BSD 2-Clause "Simplified" License, GNU Lesser General Public License (LGPL) 2.1, CC-By Attribution 4.0 International, Artistic License 2.0, CC0 1.0 Universal, Apache License 2.0, Mozilla Public License 2.0, Academic Free License (AFL) 3.0, Eclipse Public License 1.0, MIT License, GNU General Public License (GPL) 3.0, GNU General Public License (GPL) 2.0<br>R: No reader registration required	*Journal submission:* Unrestricted - manuscript can be published in any destination<br><br>*Journals directly associated with platform:* None<br><br>*Facilitated submission to other journals:* None<br><br>PL: Can be added by author	EI: Google Scholar, SHARE, Microsoft Academic, Unpaywall<br><br>MA: Openly available via API (application programming interface)<br><br>MR: No (planned in development roadmap)
AgriXiv [2] – *Verified*	Research manuscripts only	Cyrillic, English, Indonesian, unknown if others	S: Any format supported by OSF platform<br>V: By download in file format submitted by author(s), PDF viewable in browser without download<br>I: DOI (10.31220/osf.io)<br>VP: Accepts any new versions / single DOI retained for all versions<br>L: Authors are provided with a choice of licenses: CC0 1.0 Universal, CC-By Attribution 4.0 International<br>R: No reader registration required	*Journal submission:* Unrestricted - manuscript can be published in any destination<br><br>*Journals directly associated with platform:* None<br><br>*Facilitated submission to other journals:* None<br><br>PL: Can be added by author	EI: Google Scholar, SHARE, Microsoft Academic, Unpaywall<br><br>MA: Openly available via API<br><br>MR: No (planned in development roadmap)
Arabixiv [3] – *Verified*	Any scholarly content	Arabic, English	S: PDF<br>V: By download in file format submitted by author(s), PDF viewable in browser without download<br>I: DOI (10.31221/osf.io)<br>VP: Accepts any new versions / single DOI retained for all versions<br>L: Authors are provided with a choice of licenses: No license, GNU Lesser General Public License (LGPL) 3.0, BSD 3-Clause "New"/"Revised" License, BSD 2-	*Journal submission:* Unrestricted - manuscript can be published in any destination<br><br>*Journals directly associated with platform:* None<br><br>*Facilitated submission to other journals:* None<br><br>PL: Can be added by author	EI: Google Scholar, SHARE, Microsoft Academic, Unpaywall<br><br>MA: Openly available via API<br><br>MR: No (planned in development roadmap)

			Clause "Simplified" License, GNU Lesser General Public License (LGPL) 2.1, CC-By Attribution 4.0 International, Artistic License 2.0, CC0 1.0 Universal, Apache License 2.0, Mozilla Public License 2.0, Academic Free License (AFL) 3.0, Eclipse Public License 1.0, MIT License, GNU General Public License (GPL) 3.0, GNU General Public License (GPL) 2.0<br>R: No reader registration required		
EcoEvoRxiv [4] – *Verified*	All article types, datasets (primary and secondary research)	English	S: PDF<br>V: By download in file format submitted by author(s), PDF viewable in browser without download<br>I: DOI (10.32942/osf.io)<br>VP: Accepts any new versions / single DOI retained for all versions<br>L: Authors are provided with a choice of licenses: CC-By Attribution-ShareAlike 4.0 International, CC-By Attribution-NonCommercial-NoDerivatives 4.0 International, CC-By Attribution 4.0 International, CC0 1.0 Universal, CC-By Attribution-ShareAlike 4.0 International, CC-By Attribution-NonCommercial-NoDerivatives 4.0 International, with none preferred<br>R: No reader registration required	*Journal submission:* Unrestricted - manuscript can be published in any destination<br><br>*Journals directly associated with platform:* None<br><br>*Facilitated submission to other journals:* None<br><br>PL: Can be added by author	EI: Google Scholar, SHARE, Microsoft Academic, Unpaywall<br><br>MA: Openly available via API<br><br>MR: No (planned in development roadmap)
FocUS Archive [5] – *Verified*	Unknown	Unknown	S: Any format supported by OSF platform<br>V: By download in file format submitted by author(s), PDF viewable in browser without download<br>I: DOI (10.31225/osf.io)<br>VP: Accepts any new versions / single DOI retained for all versions<br>L: Authors must use CC-BY license<br>R: No reader registration required	*Journal submission:* Unrestricted - manuscript can be published in any destination<br><br>*Journals directly associated with platform:* None<br><br>*Facilitated submission to other journals:* None<br><br>PL: Can be added by author	EI: Google Scholar, SHARE, Microsoft Academic, Unpaywall<br><br>MA: Openly available via API<br><br>MR: No (planned in development roadmap)
Frenxiv [6] – *Verified*	All article types, datasets, posters (primary and secondary research)	Any language (including Arabic, English, French, Indonesian)	S: PDF, Word doc, LaTeX<br>V: By download in file format submitted by author(s), PDF viewable in browser without download<br>I: DOI (10.31226/osf.io)<br>VP: Accepts any new versions / single DOI retained for all versions<br>L: Authors are provided with a choice of licenses: GNU Lesser General Public License (LGPL) 3.0, BSD 3-Clause "New"/"Revised" License, BSD 2-Clause "Simplified" License, GNU Lesser General Public License (LGPL) 2.1, CC-By Attribution 4.0 International, Artistic License 2.0, CC0 1.0 Universal,	*Journal submission:* Unrestricted - manuscript can be published in any destination<br><br>*Journals directly associated with platform:* None<br><br>*Facilitated submission to other journals:* None<br><br>PL: Can be added by author	EI: Google Scholar, SHARE, Microsoft Academic, Unpaywall<br><br>MA: Openly available via API<br><br>MR: No (planned in development roadmap)

Kirkham JJ, *et al. BMJ Open* 2020; 10:e041849. doi: 10.1136/bmjopen-2020-041849

			Apache License 2.0, Mozilla Public License 2.0, Academic Free License (AFL) 3.0, Eclipse Public License 1.0, MIT License, GNU General Public License (GPL) 3.0, GNU General Public License (GPL) 2.0, No license<br>R: No reader registration required		
INA-Rxiv [7] – *Verified*	Multiple types (including research manuscripts, essays, datasets, multimedia files; types not accepted are: thesis, report, draft book)	English, Indonesian	S: PDF, Word doc, LaTeX, MarkDown<br>V: By download in file format submitted by author(s), PDF viewable in browser without download<br>I: DOI (10.31227/osf.io)<br>VP: Accepts any new versions / single DOI retained for all versions<br>L: Authors are provided with a choice of licenses: GNU Lesser General Public License (LGPL) 3.0, BSD 3-Clause "New"/"Revised" License, BSD 2-Clause "Simplified" License, GNU Lesser General Public License (LGPL) 2.1, CC-By Attribution 4.0 International, Artistic License 2.0, CC0 1.0 Universal, Apache License 2.0, Mozilla Public License 2.0, Academic Free License (AFL) 3.0, Eclipse Public License 1.0, MIT License, GNU General Public License (GPL) 3.0, GNU General Public License (GPL) 2.0, No license, with CC-BY preferred<br>R: No reader registration required	*Journal submission:* Unrestricted - manuscript can be published in any destination<br><br>*Journals directly associated with platform:* None<br><br>*Facilitated submission to other journals:* None<br><br>PL: Can be added by author	EI: Google Scholar, SHARE, Microsoft Academic, Unpaywall<br><br>MA: Openly available via API<br><br>MR: No (planned in development roadmap)
MarXiv [8] – *note: no longer on the OSF – Verified*	Any scholarly content	Unknown	S: PDF, LaTeX<br>V: By download in file format submitted by author(s), PDF viewable in browser without download<br>I: DOI (10.31230/osf.io)<br>VP: Accepts any new versions / single DOI retained for all versions<br>L: Authors are provided with a choice of licenses: CC-BY, CC-BY-NC-ND, No License, GNU Lesser General Public License (LGPL) 3.0, Eclipse Public License 1.0, CC-BY-SA; with CC-BY preferred<br>R: No reader registration required	*Journal submission:* Unrestricted - manuscript can be published in any destination<br><br>*Journals directly associated with platform:* None<br><br>*Facilitated submission to other journals:* None<br><br>PL: Can be added by author	EI: Google Scholar, SHARE, Microsoft Academic, Unpaywall, Altmetric, OpenChannels.org<br><br>MA: Openly available via API<br><br>MR: No (planned in development roadmap)
MetaArXiv [9] – *Verified*	Multiple types (including manuscripts, theses, conference proceedings, tutorials)	English	S: PDF, Word doc, WordPerfect, LaTeX<br>V: By download in file format submitted by author(s), PDF viewable in browser without download<br>I: DOI (10.31222/osf.io)<br>VP: Accepts any new versions / single DOI retained for all versions<br>L: Authors are provided with a choice of licenses: CC0 1.0 Universal, CC-By Attribution 4.0 International<br>R: No reader registration required	*Journal submission:* Unrestricted - manuscript can be published in any destination<br><br>*Journals directly associated with platform:* None<br><br>*Facilitated submission to other journals:* None<br><br>PL: Can be added by author	EI: Google Scholar, SHARE, Microsoft Academic, Unpaywall<br><br>MA: Openly available via API<br><br>MR: No (planned in development roadmap)

Kirkham JJ, *et al. BMJ Open* 2020; 10:e041849. doi: 10.1136/bmjopen-2020-041849

MindRxiv [10] – *Verified*	Research manuscripts only	English	S: Any format supported by OSF platform (PDF preferred)<br>V: By download in file format submitted by author(s), PDF viewable in browser without download<br>I: DOI (10.31231/osf.io)<br>VP: Accepts any new versions / single DOI retained for all versions<br>L: Authors are provided with a choice of licenses: CC0, CC-BY, no license required; with CC0 preferred<br>R: No reader registration required	*Journal submission:* Unrestricted - manuscript can be published in any destination<br>*Journals directly associated with platform:* None<br>*Facilitated submission to other journals:* None<br>PL: Can be added by author	EI: Google Scholar, SHARE, Microsoft Academic, Unpaywall<br>MA: Openly available via API<br>MR: No (planned in development roadmap)
NutriXiv [11] – *Verified*	Unknown	Unknown	S: Any format supported by OSF platform<br>V: By download in file format submitted by author(s), PDF viewable in browser without download<br>I: DOI (10.31232/osf.io)<br>VP: Accepts any new versions / single DOI retained for all versions<br>L: Authors must use CC0 license<br>R: No reader registration required	*Journal submission:* Unrestricted - manuscript can be published in any destination<br>*Journals directly associated with platform:* None<br>*Facilitated submission to other journals:* None<br>PL: Can be added by author	EI: Google Scholar, SHARE, Microsoft Academic, Unpaywall<br>MA: Openly available via API<br>MR: No (planned in development roadmap)
OSF Preprints [12] – *Verified*	Multiple types (including research articles, posters, new data)	Any language	S: PDF, Word doc<br>V: By download in file format submitted by author(s), PDF viewable in browser without download<br>I: DOI (10.31219/osf.io)<br>VP: Accepts any new versions / single DOI retained for all versions<br>L: Authors are provided with a choice of licenses: CC0, CC-BY, no license; with no preference for which license chosen<br>R: No reader registration required	*Journal submission:* Unrestricted - manuscript can be published in any destination<br>*Journals directly associated with platform:* None<br>*Facilitated submission to other journals:* None<br>PL: Can be added by author	EI: Google Scholar, SHARE<br>MA: Openly available via API<br>MR: No (planned in development roadmap)
PaleorXiv [13] – *Verified*	Multiple article types (including systematic reviews, data papers, methods papers)	Any language – non-English languages held in moderation queue awaiting verification	S: PDF, LaTeX, text<br>V: By download in file format submitted by author(s), PDF viewable in browser without download<br>I: DOI (10.31233/osf.io)<br>VP: Accepts any new versions / single DOI retained for all versions<br>L: Authors must use CC-BY license<br>R: No reader registration required	*Journal submission:* Unrestricted - manuscript can be published in any destination<br>*Journals directly associated with platform:* None<br>*Facilitated submission to other journals:* None<br>PL: Can be added by author	EI: Google Scholar, SHARE, Microsoft Academic, Unpaywall<br>MA: Openly available via API<br>MR: No (planned in development roadmap)
PsyArXiv [14] – *Verified*	Any scholarly content	Unknown	S: PDF, Word doc<br>V: By download in file format submitted by author(s), PDF viewable in browser without download<br>I: DOI (10.31234/osf.io)<br>VP: Accepts any new versions / single DOI retained for all versions<br>L: Authors are provided with a choice of licenses: CC0,	*Journal submission:* Unrestricted - manuscript can be published in any destination<br>*Journals directly associated with platform:* None<br>*Facilitated submission to other journals:* None	EI: Google Scholar, SHARE, Microsoft Academic, Unpaywall<br>MA: Openly available via API<br>MR: No (planned in development

			CC-BY, no license; with no preference stated<br>R: No reader registration required	PL: Can be added by author	roadmap)
SocArxiv [15] – *Verified*	Research manuscripts only	Unknown	S: Any format supported by OSF platform<br>V: By download in file format submitted by author(s), PDF viewable in browser without download<br>I: DOI (10.31235/osf.io)<br>VP: Accepts any new versions / single DOI retained for all versions<br>L: Authors are provided with a choice of licenses: CC0, CC-BY, no license; with CC0 or CC-BY preferred<br>R: No reader registration required	*Journal submission:* Unrestricted - manuscript can be published in any destination<br><br>*Journals directly associated with platform:* None<br><br>*Facilitated submission to other journals:* None<br><br>PL: Can be added by author	EI: Google Scholar, SHARE, Microsoft Academic, Unpaywall<br><br>MA: Openly available via API<br><br>MR: No (planned in development roadmap)
SportRxiv [16] – *Verified*	All article types (including research manuscripts, commentaries, review articles, current opinions, case studies, technical reports, short communications)	Any language – non-English languages held in moderation queue awaiting verification	S: PDF<br>V: By download in file format submitted by author(s), PDF viewable in browser without download<br>I: DOI (10.31236/osf.io)<br>VP: Accepts any new versions / single DOI retained for all versions<br>L: Authors are provided with a choice of licenses: CC0, CC-BY; with CC-BY preferred<br>R: No reader registration required	*Journal submission:* Unrestricted - manuscript can be published in any destination<br><br>*Journals directly associated with platform:* None<br><br>*Facilitated submission to other journals:* None<br><br>PL: Can be added by author	EI Google Scholar, SHARE, Microsoft Academic, Unpaywall<br><br>MA: Openly available via API<br><br>MR: No (planned in development roadmap)
Thesis Commons [17] – *Verified*	Theses only	Any language	S: PDF, Word doc<br>V: By download in file format submitted by author(s), PDF viewable in browser without download<br>I: DOI (10.31237/osf.io)<br>VP: Accepts any new versions / single DOI retained for all versions<br>L: Authors are provided with a choice of licenses: CC0, CC-BY; with no preference stated<br>R: No reader registration required	*Journal submission:* Unrestricted - manuscript can be published in any destination<br><br>*Journals directly associated with platform:* None<br><br>*Facilitated submission to other journals:* None<br><br>PL: Can be added by author	EI: Google Scholar, SHARE, Microsoft Academic, Unpaywall<br><br>MA: Openly available via API<br><br>MR: No (planned in development roadmap)
Open Research Central infrastructure					
AAS Open Research [18] – *Verified*<br><br>AMRC Open Research [19] – *Verified*<br><br>Gates Open Research [20] – *Verified*<br><br>HRB Open Research [21] – *Verified*	All article types (primary and secondary research)<br><br>Editorials (Wellcome Open Research only)<br><br>Posters and slides (AAS, AMRC, Gates	English (articles)<br><br>Accepts posters and slides in all languages – metadata and description must be in English	S: Word doc, RTF (Wellcome Open Research also supports LaTeX via Overleaf template)<br>V: By PDF download, full-text viewable in browser without PDF (e.g. as HTML), by XML download<br>I: DOI (10.12688/aasopenres; …/amrcopenres; …/gatesopenres; …/hrbopenres; …/mniopenres; …/wellcomeopenres)<br>VP: Accepts any new versions / each version receives its own DOI and own non-DOI citable identifier<br>L: Authors must use CC-BY license<br>R: No reader registration required	*Journal submission:* Restricted - manuscript can only be published in journal linked to server (AAS/AMRC/Gates/HRB/MNI/Wellcome Open Research, respectively)<br><br>PL: Yes, added by platform within one month of publication	EI: Google Scholar, PrePubMed, Europe PMC, SciLit<br><br>MA: Available for free upon request (AAS/AMRC/Gates/HRB/MNI); Openly available via API (Wellcome Open Research)<br><br>MR: Yes (XML and HTML)

MNI Open Research [22] – *Verified*	Open Research only)				
Wellcome Open Research [23] – *Verified*					
**Others**					
arXiv [24] – *Verified*	Research manuscripts only	Any language – abstract must be in English	S: PDF, LaTeX, HTML<br>V: PDF (and other format(s) as supplied by author) viewable in browser without download<br>I: Platform-specific ID (arXiv ID)<br>VP: Accepts any new versions / each version receives its own non-DOI citable identifier<br>L: Authors are provided with a choice of licenses: CC0, CC-BY, CC-BY-SA 4.0, CC-BY-NC-SA 4.0, non-exclusive license to distribute, any other CC license as specified in manuscript text; with no preference for which license chosen<br>R: No reader registration required	*Journal submission:* Unrestricted - manuscript can be published in any destination<br><br>*Journals directly associated with platform:* None<br><br>*Facilitated submission to other journals:* Not available<br><br>PL: Yes, added by platform after one month from publication	EI: Google Scholar, PrePubMed (q-bio only), Europe PMC, SciLit, SHARE, INSPIRE-HEP, The NASA Astrophysics Data System (ADS), The arXiv Search Interface from the National Science Library, Chinese Academy of Sciences (also in Chinese)<br><br>MA: Openly available via API<br><br>MR: Require full-text to be submitted in machine-readable format
Authorea [25] – *Verified*	Any scholarly content (including articles, datasets, code, figures, tables, slides, micropublications, and Jupyter Notebooks)	Any language	S: PDF, Word doc, LaTeX<br>V: Full-text viewable in browser without PDF (e.g. as HTML), by LaTeX download<br>I: DOI (10.22541)<br>VP: Accepts any new versions / each version receives its own DOI<br>L: Authors must use CC-BY license<br>R: No reader registration required	*Journal submission:* Unrestricted - manuscript can be published in any destination<br><br>*Journals directly associated with platform*: None<br><br>*Facilitated submission to other journals:* Yes, listed at https://support.authorea.com/en-us/article/how-do-i-directly-submit-to-a-publisher-atxzf7/<br><br>PL: Yes, added by platform within one month of publication	EI: SciLit, Crossref<br><br>MA: Unknown<br><br>MR: Yes (HTML)
bioRxiv [26] – *Verified*	Research manuscripts only (primary research only; white papers accepted in Scientific Communication and Education category only; types not accepted	English – non-English language translations can be included as supplementary files	S: PDF, Word doc, WordPerfect<br>V: By PDF download, PDF viewable in browser without download, full-text viewable in browser without PDF (e.g. as HTML)<br>I: DOI (10.1101)<br>VP: Accepts new versions until acceptance at journal / single DOI retained but new versions have distinct URLs<br>L: Authors are provided with a choice of licenses: CC0, CC-BY, CC-BY-NC, CC-BY-ND, CC-BY-NC-ND, or no	*Journal submission:* Unrestricted - manuscript can be published in any destination<br><br>*Journals directly associated with platform:* None<br><br>*Facilitated submission to other journals:* Yes, listed at https://www.biorxiv.org/about-biorxiv<br><br>PL: Yes, added by platform within one month of publication	EI: Google Scholar, Microsoft Academic, Meta, EuropePMC, SHARE, Crossref<br><br>MA: Openly available via API<br><br>MR: Yes (HTML)

	are: datasets, theses, figures, protocols, correspondence without new data or analyses)		reuse; with no preference for which license chosen<br>R: No reader registration required		
Cell Press Sneak Peek[b] [27] – *Verified*	Content criteria as directed by associated journal(s) (Cell Press portfolio)	Unknown	S: PDF<br>V: By PDF download, PDF viewable in browser without download<br>I: URL only<br>VP: Unknown / Unknown<br>L: Unknown<br>R: Reader can view basic metadata without account but must register to view and download full content	*Journal submission:* Manuscripts must be submitted to a Cell Press Journal first, authors are then provided with the option of displaying their manuscript while under review using SSRN's 'First Look' (aka 'Sneak Peek')<br><br>PL: Unknown	EI: Unknown<br><br>MA: Unknown<br><br>MR: No
ChemRxiv [28]	Research manuscripts only	English	S: Any (PDF)<br>V: By PDF download, PDF viewable in browser without download<br>I: DOI (10.26434)<br>VP: Accepts new versions until acceptance at journal / each version receives its own DOI suffix<br>L: Authors are provided with a choice of licenses: CC-BY, CC-BY-NC, CC-BY-NC-ND; with CC-BY-NC-ND preferred<br>R: No reader registration required	*Journal submission:* Unrestricted - manuscript can be published in any destination<br><br>*Journals directly associated with platform:* None<br><br>*Facilitated submission to other journals:* Yes, journals owned by American Chemical Society, Royal Society of Chemistry, German Chemical Society (other publishers coming soon)<br><br>PL: Yes, added by platform within one month of publication	EI: Google Scholar, EuropePMC, Chemical Abstracts Services, Crossref<br><br>MA: Openly available via API<br><br>MR: If provided by author in machine-readable format
ChinaXiv [29]	Research manuscripts only	Standard Chinese, English, unknown if other	S: PDF, Word doc, LaTeX<br>V: By PDF download<br>I: DOI (10.12074)<br>VP: Accepts any new versions / single DOI retained for all versions<br>L: Authors are provided with a choice of licenses: CC0, CC-BY, CC-BY-SA, CC-BY-NC-SA; with no preference for which license chosen<br>R: No reader registration required	*Journal submission:* Unrestricted - manuscript can be published in any destination<br><br>*Journals directly associated with platform:* Multiple, listed at http://chinaxiv.org/journal/browse.htm<br><br>*Facilitated submission to other journals:* Not available<br><br>PL: Can be added by author or journal	EI: ROAR (Registry of Open Access Repositories)<br><br>MA: Openly available via API<br><br>MR: No
ESSOAr [30] – *Verified*	Scholarly research content (including manuscripts and posters)	English	S: PDF (Word and LaTeX in development)<br>V: By PDF download<br>I: DOI (10.1002)<br>VP: Accepts new versions until acceptance at journal / each version receives its own DOI suffix<br>L: Authors are provided with a choice of licenses:	*Journal submission:* Unrestricted - manuscript can be published in any destination<br><br>*Journals directly associated with platform:* 21 AGU publications (others in roadmap)	EI: Google Scholar, SHARE, Crossref<br><br>MA: Openly available via Crossref<br><br>MR: Yes

			CC-BY, CC-BY-NC, CC-BY-NC-ND, non-exclusive license; with no preference stated<br>R: No reader registration required	*Facilitated submission to other journals:* Not yet (on roadmap)<br><br>PL: Yes, added by platform within one month of publication; can be added by author or journal	
F1000 Research [31] – *Verified*	Any scholarly content (including articles, posters and slides; primary and secondary research)	English	S: Word doc, RTF, LaTeX via Overleaf only<br>V: By PDF download, full-text viewable in browser without PDF, by XML download<br>I: DOI (10.12688/f1000research)<br>VP: Accepts any new versions / each version receives its own DOI and own non-DOI citable identifier<br>L: Authors must use CC-BY license<br>R: No reader registration required	*Journal submission:* Restricted - manuscript can only be published in journal linked to server (F1000 Research)<br><br>PL: Yes, added by platform within one month of publication	EI: Google Scholar, Europe PMC, SHARE, SciLit, PrePubMed<br><br>MA: Openly available via API<br><br>MR: Yes (XML and HTML)
JMIR Preprints [32]	Research manuscripts, blog posts, "grey" online reports	Unknown	S: Word doc<br>V: By PDF download, PDF viewable in browser without download<br>I: DOI (10.2196/preprints.)<br>VP: Accepts first submission only / not applicable<br>L: Authors must use no license (all rights reserved)<br>R: Author choice: No reader registration required, or reader can view basic content (title, abstract) without account but must register to view and download full content	*Journal submission:* Unrestricted - manuscript can be published in any destination<br><br>*Journals directly associated with platform:* JMIR Publications journals<br><br>*Facilitated submission to other journals:* Unknown<br><br>PL: Unknown	EI: Unknown<br><br>MA: Unknown<br><br>MR: No
medRxiv [33] – *Verified*	Multiple article types (including research articles, systematic reviews and meta-analysis, clinical research design protocols, data articles; types not accepted are: theses, opinion pieces, editorials, narrative reviews, datasets)	English	S: PDF, Word doc, WordPerfect<br>V: By PDF download, PDF viewable in browser without download<br>I: DOI (10.1101)<br>VP: Accepts new versions until acceptance at journal / single DOI retained for all versions<br>L: Authors are provided with a choice of licenses: CC0, CC-BY, CC-BY-NC, CC-BY-ND, CC-BY-NC-ND, or no reuse; with no preference for which license chosen<br>R: No reader registration required	*Journal submission:* Unrestricted - manuscript can be published in any destination<br><br>*Journals directly associated with platform:* None<br><br>*Facilitated submission to other journals:* Yes, listed at https://www.medrxiv.org/content/about-medrxiv<br><br>PL: Yes, added by platform within one month of publication	EI: Google Scholar, Microsoft Academic Search, Crossref<br><br>MA: Openly available via Crossref<br><br>MR: No
MitoFit Preprint Archives [34]	Any scholarly content (primary and secondary research)	English	S: PDF (abstract to be submitted as Word doc)<br>V: By PDF download<br>I: DOI (10.26124)<br>VP: Accepts new versions until acceptance at journal / each version receives its own DOI suffix<br>L: Authors must use CC-BY-NC-ND license<br>R: No reader registration required	*Journal submission:* Unrestricted - manuscript can be published in any destination<br><br>*Journals directly associated with platform:* None<br><br>*Facilitated submission to other journals:* Unknown<br><br>PL: Yes, added by platform within one month of	EI: Google Scholar<br><br>MA: Openly available via DataCite<br><br>MR: No

				publication	
NeuroImage: Clinical - *First Look*[b] [35]	Content criteria as directed by associated journal (NeuroImage: Clinical)	Unknown	S: PDF, Word doc<br>V: By PDF download, PDF viewable in browser without download<br>I: URL only<br>VP: Unknown / Unknown<br>L: Unknown<br>R: No reader registration required	*Journal submission:* Manuscripts must be submitted to the NeuroImage: Clinical first, authors are then provided with the option of displaying their manuscript while under review using SSRN's 'First Look'<br><br>PL: Unknown	EI: Unknown<br><br>MA: Unknown<br><br>MR: No
PeerJ Preprints [36] – *Verified*	Any scholarly content (primary and secondary research)	English	S: PDF<br>V: By PDF download, PDF viewable in browser without download, by XML download<br>I: DOI (10.7287)<br>VP: Accepts any new versions until publication in a journal / each version receives its own non-DOI citable identifier<br>L: Authors are provided with a choice of licenses: CC0, CC-BY; with CC-BY preferred<br>R: No reader registration required	*Journal submission:* Unrestricted - manuscript can be published in any destination<br><br>*Journals directly associated with platform:* PeerJ journals<br><br>*Facilitated submission to other journals:* Unknown<br><br>PL: Can be added by author or journal	EI: Google Scholar, Europe PMC<br><br>MA: Openly available via Crossref<br><br>MR: No
Preprints with The Lancet[b] [37] – *Verified*	Content criteria as directed by associated journal(s) (Lancet family of journals)	Unknown	S: Unknown<br>V: By PDF download, PDF viewable in browser without download<br>I: URL only<br>VP: Accepts first submission only / not applicable<br>L: Unknown<br>R: Reader can view basic content (title, abstract) without account but must register to view and download full content	*Journal submission:* Manuscripts must be submitted to the Lancet family of journals (including EBioMedicine and EClinicalMedicine) first, authors are then provided with the option of displaying their manuscript while under review using SSRN's 'First Look'<br><br>PL: Can be added by author or journal	EI: Unknown<br><br>MA: Unknown<br><br>MR: No
Preprints.org [38]	Multiple article types (including research manuscripts, comprehensive reviews, data articles, case reports; types not accepted are: editorials, discussion papers, commentaries, coursework)	English	S: Word doc, LaTeX<br>V: By PDF download<br>I: DOI (10.20944)<br>VP: Accepts any new versions / each version receives its own DOI and own non-DOI citable identifier<br>L: Authors must use CC-BY license<br>R: No reader registration required	*Journal submission:* Unrestricted - manuscript can be published in any destination<br><br>*Journals directly associated with platform:* None<br><br>*Facilitated submission to other journals:* Not available<br><br>PL: Unknown	EI: Google Scholar, PrePubMed, Europe PMC, SHARE, SciLit<br><br>MA: Openly available via Crossref<br><br>MR: No
Research Square [39] –	Multiple article	English	S: Word doc, LaTeX	*Journal submission:* Unrestricted - manuscript	EI: Google Scholar, Crossref,

Kirkham JJ, *et al. BMJ Open* 2020; 10:e041849. doi: 10.1136/bmjopen-2020-041849

Verified	types (including research articles, systematic reviews, method articles, data articles; types not accepted are: literature reviews, hypotheses, opinions, theories or commentaries)		V: By PDF download, full-text viewable in browser without PDF (e.g. as HTML)<br>I: DOI (10.21203)<br>VP: Accepts any new versions / each version receives its own DOI<br>L: Authors must use CC-BY license<br>R: No reader registration required	can be published in any destination<br><br>*Journals directly associated with platform:* Submit directly or submit from BMC journals and more from Springer Nature portfolio listed at https://www.researchsquare.com/journals<br><br>*Facilitated submission to other journals:* Not available<br><br>PL: Yes, added by platform within one month of publication	Researcher-app<br><br>MA: Openly available via Crossref<br><br>MR: Yes (HTML)
SciELO Preprints [40] – *Verified*	Research manuscripts only	English, Portuguese, Spanish	S: PDF or any PDF-convertible format<br>V: By PDF download, PDF viewable in browser without download<br>I: URL only<br>VP: Accepts new versions until acceptance at journal / single DOI retained for all versions<br>L: Authors must use CC-BY license<br>R: No reader registration required	*Journal submission:* Unrestricted - manuscript can be published in any destination<br><br>*Journals directly associated with platform:* None (SciELO journals when full operation launched)<br><br>*Facilitated submission to other journals:* Available for any journal using a SWORD (Simple Web-service Offering Repository Deposit) protocol-based manuscript submission platform<br><br>PL: Can be added by author or journal	EI: Google Scholar<br><br>MA: Openly available via API<br><br>MR: No
SSRN [41] – *Verified*	Multiple types (including research manuscripts, abstracts, editorials, opinion papers)	Any language – English preferred, title and abstract must be provided in English, non-English translations can be submitted and linked to English version	S: PDF<br>V: By PDF download, PDF viewable in browser without download<br>I: DOI (10.2139)<br>VP: Accepts any new versions / unknown<br>L: Unknown<br>R: No reader registration required	*Journal submission:* Unrestricted - manuscript can be published in any destination<br><br>*Journals directly associated with platform:* None<br><br>*Facilitated submission to other journals:* Not available<br><br>PL: Unknown	EI: Unknown<br><br>MA: Unknown<br><br>MR: No
Surgery Open Science – *First Look*[b] [42] – *Verified*	Content criteria as directed by associated journal (Surgery Open Science)	Unknown	S: Unknown<br>V: By PDF download, PDF viewable in browser without download<br>I: URL only<br>VP: Unknown<br>L: Unknown<br>R: Reader can view basic content (title, abstract) without account but must register to view and download full content	*Journal submission:* Manuscripts must be submitted to Surgery Open Science first, authors are then provided with the option of displaying their manuscript while under review using SSRN's 'First Look'<br><br>PL: Unknown	EI: Unknown<br><br>MA: Unknown<br><br>MR: No
Therapoid [43] –	Research	English – Chinese	S: PDF	*Journal submission:* Unrestricted – manuscript	EI: Not indexed

Verified	manuscripts only (multimedia content accepted in associated discussion forum)	also to be supported in future	V: By PDF download, PDF viewable in browser without download<br>I: URL only<br>VP: Accepts any new versions / each version receives its own DOI<br>L: Authors must use CC-BY license<br>R: No reader registration required	can be published in any destination<br><br>*Journals directly associated with platform:* None<br><br>*Facilitated submission to other journals:* Not available yet (to be provided for any and only open access journals)<br><br>PL: Yes, added by platform within one month of publication	MA: Openly available via Crossref<br><br>MR: No (plan to require HTML in future)
ViXra [44]	Multiple types (including research articles, books, theses)	Any language – English preferred, non-English translations can be provided with English version, as new versions or submitted separately	S: PDF<br>V: By PDF download, PDF viewable in browser without download<br>I: URL, Platform-specific ID<br>VP: Accepts any new versions / each version retains ID with a version number<br>L: Authors can use any license<br>R: No reader registration required	*Journal submission:* Unrestricted - manuscript can be published in any destination<br><br>*Journals directly associated with platform:* None<br><br>*Facilitated submission to other journals:* Not available<br><br>PL: Can be added by author or journal	EI: Not indexed<br><br>MA: Unknown<br><br>MR: No

[a] Where primary research is research based on empirical data and secondary research is derived from primary research, such as literature reviews and meta-reviews; [b] See SSRN for general platform information

Preprint platform websites

1. AfricArxiv https://info.africarxiv.org/
2. AgriXiv https://agrixiv.org
3. Arabixiv https://arabixiv.org/
4. EcoEvoRxiv https://ecoevorxiv.org
5. FocUS Archive https://osf.io/preprints/focusarchive/
6. Frenxiv https://frenxiv.org
7. INA-Rxiv https://osf.io/preprints/inarxiv
8. MarXiv https://marxiv.org
9. MetaArXiv https://osf.io/preprints/metaarxiv/
10. MindRxiv https://mindrxiv.org
11. NutriXiv https://osf.io/preprints/nutrixiv
12. OSF Preprints https://osf.io/preprints/
13. PaleorXiv https://paleorxiv.org
14. PsyArXiv https://psyarxiv.com
15. SocArXiv https://osf.io/preprints/socarxiv
16. SportRxiv https://osf.io/preprints/sportrxiv
17. Thesis Commons https://thesiscommons.org
18. AAS Open Research https://aasopenresearch.org/
19. AMRC Open Research https://amrcopenresearch.org/
20. Gates Open Research https://gatesopenresearch.org/
21. HRB Open Research https://hrbopenresearch.org/
22. MNI Open Research https://mniopenresearch.org/
23. Wellcome Open Research https://wellcomeopenresearch.org/
24. arXiv https://arxiv.org
25. Authorea https://www.authorea.com
26. bioRxiv https://www.biorxiv.org/
27. Cell Press Sneak Peek https://papers.ssrn.com/sol3/Jeljour_results.cfm?form_name=journalBrowse&journal_id=3184889
28. ChemRxiv https://chemrxiv.org
29. ChinaXiv http://chinaxiv.org
30. ESSOAr https://www.essoar.org
31. F1000 Research https://www.essoar.org
32. JMIR Preprints https://preprints.jmir.org/
33. medRxiv https://www.medrxiv.org
34. MitoFit Preprint Archives https://www.mitofit.org/index.php/MitoFit_Preprint_Archives
35. NeuroImage: Clinical – *First Look* https://papers.ssrn.com/sol3/JELJOUR_Results.cfm?form_name=journalBrowse&journal_id=3178959
36. PeerJ Preprints https://peerj.com/preprints/
37. Preprints with The Lancet https://papers.ssrn.com/sol3/JELJOUR_Results.cfm?form_name=journalBrowse&journal_id=3184962
38. Preprints.org https://www.preprints.org/
39. Research Square https://www.researchsquare.com
40. SciELO Preprints https://preprints.scielo.org/index.php/scielo
41. SSRN https://www.ssrn.com
42. Surgery Open Science – *First Look* https://papers.ssrn.com/sol3/Jeljour_results.cfm?form_name=journalBrowse&journal_id=3303309
43. Therapoid https://therapoid.net
44. ViXra http://vixra.org
