# Supplementary material for: Systematic examination of preprint platforms for use in the medical and biomedical sciences setting
Source: BMJ Open. 2020 Dec 29;10(12):e041849. doi: 10.1136/bmjopen-2020-041849 (PMC7778769; doi:10.1136/bmjopen-2020-041849)
Supplement: Supplementary data [file bmjopen-2020-041849supp004.pdf]

Supplementary Table 4: Screening, moderation, and permanence of content

Preprint Server	Stated not peer reviewed on manuscript record webpage (M) /general server webpages (S)	Advisory Board (A) / Includes Researchers (R)	Commitment to FAIR (Findable, Accessible, Interoperable and Reusable) principles / Data availability statement (DAS)	Screening checks performed between submitting and posting (stating where this process involves active researchers)	Permanence of content (P) / Reasons for retraction (R) / Web presence of removed content (W)	Mechanisms to report concerns about posted content	Sustainability of the service (S) / Preservation of content (P)
**OSF Communities**							
AfricArxiv [1] – *Verified*	M: No<br>S: Yes	A: Yes<br>R: Yes	Yes, endorses, stated on website<br><br>DAS: Not requested or required	'moderated submission check for quality standard, good scientific practices, open science principles', including data/code availability	P: Permanent with some removal options in extraneous circumstances<br>R: Plagiarism, false or inaccurate content<br>W: Basic information remains on a tombstone page	Email administrator; helpdesk support from COS (Centre for Open Science)	S: COS receive external financial support (e.g. grant, individual); operators report no sources of income, staffed on voluntary basis<br>P: COS Preservation Fund to maintain read access for 50+ years
AgriXiv [2] – *Verified*	M: No<br>S: No	A: Yes<br>R: Yes	Yes<br><br>DAS: Unknown	No information	P: Permanent with some removal options in extraneous circumstances<br>R: Plagiarism, false or inaccurate content<br>W: Basic information remains on a tombstone page	Email administrator; helpdesk support from COS	S: COS receive external financial support (e.g. grant, individual); operators report Open Access India plans to raise funds<br>P: COS Preservation Fund to maintain read access for 50+ years
Arabixiv [3] – *Verified*	M: No<br>S: No	A: Yes<br>A: Unknown	Yes<br><br>DAS: Unknown	Arabixiv editors check for: ensuring it is academic research	P: Permanent with some removal options in extraneous circumstances<br>R: Plagiarism, false or inaccurate content<br>W: Basic information remains on a tombstone page	Email administrator; helpdesk support from COS; Readers encouraged to post concerns publicly as new article	S: COS receive external financial support (e.g. grant, individual); no further information to report from operators<br>P: COS Preservation Fund to maintain read access for 50+ years
EcoEvoRxiv [4] – *Verified*	M: No<br>S: Yes	A: Yes<br>R: Yes	Yes<br><br>DAS: Unknown	Steering committee member (*including active researchers*) checks for: competing interests, plagiarism, misconduct or integrity checks, ethical and legal compliance, funder acknowledgment	P: Permanent with some removal options in extraneous circumstances<br>R: Plagiarism, false or inaccurate content<br>W: Basic information remains on a tombstone page	Email administrator; helpdesk support from COS	S: COS receive external financial support (e.g. grant, individual); no further information to report from operators<br>P: COS Preservation Fund to maintain read access for 50+ years
FocUS Archive [5] – *Verified*	M: No<br>S: No	A: Yes<br>R: Yes	Yes<br><br>DAS: Unknown	There is a moderation process, no further information known	P: Permanent with some removal options in extraneous circumstances<br>R: Plagiarism, false or inaccurate content<br>W: Basic information remains	Email administrator; helpdesk support from COS	S: COS receive external financial support (e.g. grant, individual); nothing to report from operators<br>P: COS Preservation Fund to maintain read access for 50+

Kirkham JJ, *et al. BMJ Open* 2020; 10:e041849. doi: 10.1136/bmjopen-2020-041849

					on a tombstone page		years
Frenxiv [6] – *Verified*	M: No<br>S: No	A: Yes<br>R: Yes	Yes<br><br>DAS: Unknown	Editorial-advisory members (*including active researchers*) check for: competing interests, plagiarism, misconduct or integrity checks, authors are genuine, ethical and legal compliance, data availability	P: Permanent with some removal options in extraneous circumstances<br>R: Plagiarism, false or inaccurate content<br>W: Basic information remains on a tombstone page	Email administrator; helpdesk support from COS	S: COS receive external financial support (e.g. grant, individual); nothing to report from operators<br>P: COS Preservation Fund to maintain read access for 50+ years
INA-Rxiv [7] – *Verified*	M: No<br>S: Yes	A: Yes<br>R: Yes	Yes<br><br>DAS: Unknown	Unknown	P: Permanent with some removal options in extraneous circumstances<br>R: Plagiarism, false or inaccurate content<br>W: Basic information remains on a tombstone page	Email administrator; helpdesk support from COS	S: COS receive external financial support (e.g. grant, individual); operators endorse users to donate to COS<br>P: COS Preservation Fund to maintain read access for 50+ years
MarXiv [8] – *note: no longer on the OSF* – *Verified*	M: No<br>S: Yes	A: Yes<br>R: Yes	Yes<br><br>DAS: Unknown	The MarXiv team check for: plagiarism, misconduct or integrity checks, ethical and legal compliance	P: Permanent with some removal options in extraneous circumstances<br>R: Plagiarism, false or inaccurate content<br>W: Basic information remains on a tombstone page	Email administrator; helpdesk support from COS	S: COS receive external financial support (e.g. grant, individual); service moving to another host<br>P: COS Preservation Fund to maintain read access for 50+ years
MetaArXiv [9] – *Verified*	M: No<br>S: No	A: Yes<br>R: Yes	Yes<br><br>DAS: Not requested or required	BITSS staff check for: relevance of material, author name matches submitter	P: Permanent with some removal options in extraneous circumstances<br>R: Plagiarism, false or inaccurate content<br>W: Basic information remains on a tombstone page	Email administrator; helpdesk support from COS; Community moderation via commenting	S: COS receive external financial support (e.g. grant, individual); operation supported by BITSS funders and revenue<br>P: COS Preservation Fund to maintain read access for 50+ years
MindRxiv [10] – *Verified*	M: No<br>S: Yes	A: Yes<br>R: Yes	Yes<br><br>DAS: Not requested or required	Science Director at Mind & Life (*including active researchers*) checks for: relevance of material, ensuring it is academic research	P: Permanent with some removal options in extraneous circumstances<br>R: Plagiarism, false or inaccurate content<br>W: Basic information remains on a tombstone page	Email administrator; helpdesk support from COS	S: COS receive external financial support (e.g. grant, individual); nothing to report from operators<br>P: COS Preservation Fund to maintain read access for 50+ years
NutriXiv [11] – *Verified*	M: No<br>S: No	A: Unknown<br>R: Unknown	Yes<br><br>DAS: Unknown	No information	P: Permanent with some removal options in extraneous circumstances<br>R: Plagiarism, false or inaccurate content<br>W: Basic information remains on a tombstone page	Email administrator; helpdesk support from COS	S: COS receive external financial support (e.g. grant, individual); nothing to report from operators<br>P: COS Preservation Fund to maintain read access for 50+ years
OSF Preprints [12] –	M: No	A: Yes	Yes	OSF staff members run	P: Permanent with some	Email administrator;	S: COS receive external financial

Verified	S: Yes	R: Yes	DAS: Not requested or required	automated checks for spam	removal options in extraneous circumstances<br>R: Plagiarism, false or inaccurate content<br>W: Basic information remains on a tombstone page	helpdesk support from COS	support (e.g. grant, individual)<br>P: COS Preservation Fund to maintain read access for 50+ years
PaleorXiv [13] – *Verified*	M: No<br>S: Yes	A: Yes<br>R: Yes	Yes<br><br>DAS: Not requested or required	A moderator from the PaleoRxiv steering committee (*including active researchers*) checks for: plagiarism, relevance of material, ensuring it is academic research	P: Permanent with some removal options in extraneous circumstances<br>R: Plagiarism, false or inaccurate content<br>W: Basic information remains on a tombstone page	Email administrator; helpdesk support from COS	S: COS receive external financial support (e.g. grant, individual); nothing to report from operators<br>P: COS Preservation Fund to maintain read access for 50+ years
PsyArXiv [14] – *Verified*	M: No<br>S: Yes	A: Yes<br>R: Yes	Yes<br><br>DAS: Not requested or required	Moderation team checks for: ethical and legal compliance, relevance of material, copyright violations, spam	P: Permanent with some removal options in extraneous circumstances<br>R: Plagiarism, false or inaccurate content<br>W: Basic information remains on a tombstone page	Email administrator; helpdesk support from COS	S: COS receive external financial support (e.g. grant, individual); nothing to report from operators<br>P: COS Preservation Fund to maintain read access for 50+ years
SocArxiv [15] – *Verified*	M: No<br>S: Yes	A: Yes<br>R: Yes	Yes<br><br>DAS: Unknown	There is a moderation process, no further information known	P: Permanent with some removal options in extraneous circumstances<br>R: Plagiarism, false or inaccurate content<br>W: Basic information remains on a tombstone page	Email administrator; helpdesk support from COS	S: COS receive external financial support (e.g. grant, individual); nothing to report from operators<br>P: COS Preservation Fund to maintain read access for 50+ years
SportRxiv [16] – *Verified*	M: No<br>S: Yes	A: Yes<br>R: Yes	Yes<br><br>DAS: Not requested or required	SportRxiv editors (*including active researchers*) check for: basic scientific standards, authors are genuine, all authors provide permission to post	P: Permanent with some removal options in extraneous circumstances<br>R: W: Basic information remains on a tombstone page	Email administrator; helpdesk support from COS	S: COS receive external financial support (e.g. grant, individual); operators report membership fees help offset costs<br>P: COS Preservation Fund to maintain read access for 50+ years
Thesis Commons [17] – *Verified*	M: No<br>S: No	A: Yes<br>R: Yes	Yes<br><br>DAS: Not requested or required	There is no moderation process beyond that for OSF Preprints (OSF staff check for spam)	P: Permanent with some removal options in extraneous circumstances<br>R: Plagiarism, false or inaccurate content<br>W: Basic information remains on a tombstone page	Email administrator; helpdesk support from COS	S: COS receive external financial support (e.g. grant, individual); nothing to report from operators<br>P: COS Preservation Fund to maintain read access for 50+ years
Open Research Central infrastructure							
AAS Open Research [18] – *Verified*	M: Yes<br>S: Yes	A: Yes<br>R: Yes	Yes, compliant, stated on website	F1000's in-house editorial team check for: competing interest	P: Permanent with some removal options in	Community moderation via	S: Article processing charges, covered by funding agency for

				declaration, plagiarism, authors are genuine and at least one eligible for platform, authors provide permission to post, ethical and legal compliance, funder acknowledgment, clinical trial registration, data/code availability, minimum reporting standards	extraneous circumstances<br>R: Contravention of copyright, plagiarism, false or inaccurate content, ethical and legal issues<br>W: Basic information remains on a tombstone page	commenting	AAS, Gates, HRB, MNI and Wellcome (MNI - limited to one article per lab per year)<br>P: Preprints permanently archived in Portico
AMRC Open Research [19] – *Verified*	M: Yes<br>S: Yes	A: Not yet<br>R: Still finalising advisory board	DAS: Required				
Gates Open Research [20] – *Verified*	M: Yes<br>S: Yes	A: Yes<br>R: Yes					
HRB Open Research [21] – *Verified*	M: Yes<br>S: Yes	A: Yes<br>R: Yes					
MNI Open Research [22] – *Verified*	M: Yes<br>S: Yes	A: Not yet<br>R: Not applicable					
Wellcome Open Research [23] – *Verified*	M: Yes<br>S: Yes	A: Yes<br>R: Yes					
**Others**							
arXiv [24] – *Verified*	M: No<br>S: Yes	A: Yes<br>R: Yes	None as yet<br><br>DAS: Not requested or required	arXiv moderators (*including active researchers*) check for: plagiarism, misconduct or integrity checks, authors are genuine, ethical and legal compliance, relevance of material	P: Permanent with no removal options (as long as archive exists)<br>R: None<br>W: Not applicable	Social media or direct with authors (except copyright violations or significant issues)	S: External financial support (grants and membership program: libraries, research labs, philanthropy, government funding)<br>P: Persistent access through mirror sites, no external preservation services used as yet
Authorea [25] – *Verified*	M: No<br>S: Yes	A: No<br>R: Not applicable (no advisory board)	Yes, in process of becoming compliant, without stating on website<br><br>DAS: Not requested or required	Submissions are checked for spam	P: Content maybe removed by operator/owner only<br>R: Policy not yet in place<br>W: Not applicable (at this time)	Email administrator	S: Other business model not based on direct preprinting charges or associated journal (acquisition and investment from Atypon, part of Wiley)<br>P: Preprints permanently archived in Portico
bioRxiv [26] – *Verified*	M: Yes<br>S: Yes	A: Yes<br>R: Yes	None as yet<br><br>DAS: Not requested or required	bioRxiv staff and scientific affiliates (*including active researchers*) check for: plagiarism, misconduct or integrity checks, ethical and legal compliance, clinical trial registration, submission is within scope and no danger to human	P: Permanent with withdrawal options and (rare) removal in extraneous circumstances<br>R: Removal: Legal or biohazard reasons; Withdrawal: Plagiarism, false or inaccurate	Email administrator	S: Internal (Cold Spring Harbor Laboratory) and external financial support (multiyear grant funding from Chan Zuckerberg Initiative)<br>P: Preprints permanently archived in Portico

				health	content, ethical and legal issues, co-author(s) did not consent to posting<br>W: Withdrawn articles remain visible; For removed articles, basic information remains on a tombstone page		
Cell Press Sneak Peek [27] – *Verified*	M: Yes<br>S: Yes	A: Yes<br>R: Yes	Unknown	No information	P: Unknown<br>R: Unknown<br>W: Unknown	Unknown	S: Unknown<br>P: Unknown
ChemRxiv [28]	M: Yes<br>S: Yes	A: Yes<br>R: Yes	Yes, compliant, without stating on website<br><br>DAS: Not requested or required	PhD-level chemists (*including active researchers*) check for: competing interests declared, plagiarism, misconduct or integrity checks, authors are genuine, all authors provide permission to post, ethical and legal compliance, funder acknowledgment, external data/code availability	P: Permanent with some removal options in extraneous circumstances<br>R: Contravention of copyright, plagiarism, ethical and legal issues<br>W: Basic information remains on a tombstone page	Email administrator, or contact ethics departments of governing partners	S: Nothing to report from server<br>P: Agreement to dark archive content with TIB (Leibniz Information Centre for Science and Technology University Library)
ChinaXiv [29]	M: No<br>S: No	A: Yes<br>R: Yes	Unknown<br><br>DAS: Not requested or required	Project team and scientists (*including active researchers*) check for: plagiarism, misconduct or integrity checks, authors are genuine	P: Permanent with no removal options<br>R: Contravention of copyright, plagiarism, false or inaccurate content, ethical and legal issues<br>W: Basic information remains on a tombstone page	None	S: Nothing to report from server<br>P: Long term preservation in local
ESSOAr [30] – *Verified*	M: Yes<br>S: Yes	A: Yes<br>R: Yes	Yes, without stating on website<br><br>DAS: Not requested or required	Editorial board members (*including active researchers*) check for: plagiarism, misconduct or integrity checks, authors are genuine, all authors provide permission to post, submission is scholarly and within scope	P: Permanent with some removal options in extraneous circumstances<br>R: Copyright infringement or other ethical or legal issues the Advisory Board deems critical<br>W: Unknown	Email administrator	S: Internal financial support (investment from Wiley and AGU)<br>P: On roadmap: preprints to be permanently archived in Portico
F1000 Research [31] – *Verified*	M: Yes<br>S: Yes	A: Yes<br>R: Yes	Yes, compliant, stated on website<br><br>DAS: Required	F1000's in-house editorial team check for: competing interest declaration, plagiarism, authors are genuine and at least one eligible for platform, authors provide permission to post, ethical and legal compliance, funder acknowledgment, clinical	P: Permanent with some removal options in extraneous circumstances<br>R: Contravention of copyright, false or inaccurate content, ethical and legal issues<br>W: Basic information remains on a tombstone page	Email administrator	S: Nothing to report from server<br>P: Preprints permanently archived in Portico

				trial registration, data/code availability, minimum reporting standards, relevance of material			
JMIR Preprints [32]	M: No<br>S: Yes	A: Unknown<br>R: Unknown	Unknown	Unknown	P: Unknown<br>R: Unknown<br>W: Unknown	Unknown	S: Unknown<br>P: Unknown
medRxiv [33] – *Verified*	M: Yes<br>S: Yes	A: In formation (January 2020)<br>R: Yes	None as yet<br><br>DAS: Required	Submission is checked for: competing interests declared, plagiarism, misconduct or integrity checks, minimal reporting standards, ethical and legal compliance, clinical trial registration, funder acknowledgment, submission is within scope and no danger to human health	P: Permanent with withdrawal options and (rare) removal in extraneous circumstances<br>R: Removal: Legal or biohazard reasons; Withdrawal: Plagiarism, false or inaccurate content, ethical and legal issues, co-author(s) did not consent to posting<br>W: Withdrawn articles remain visible; For removed articles, basic information remains on a tombstone page	Unknown	S: Supported by CSHL<br>P: Preprints permanently archived in Portico
MitoFit Preprint Archives [34]	M: Yes<br>S: Yes	A: Yes<br>R: Yes	Yes, endorses, stated on website<br><br>DAS: Encouraged	Scientific Advisory Board members (*including active researchers*) check for: competing interests declared, misconduct or integrity checks, authors are genuine, funder acknowledgment	P: Permanent with some removal options in extraneous circumstances<br>R: Plagiarism, ethical and legal issues, no appropriate declaration of conflicts of interest<br>W: Basic information remains on a tombstone page	Community moderation via commenting	S: Other business model not based on direct preprinting charges or associated journal (maintained by MitoFit DOI Data Center)<br>P: Backup kept in secondary server
NeuroImage: Clinical - *First Look* [35] – *Verified*	M: Yes<br>S: Yes	A: Unknown<br>R: Unknown	Unknown	No information	P: Unknown<br>R: Unknown<br>W: Unknown	Unknown	S: Unknown<br>P: Unknown
PeerJ Preprints [36] – *Verified*	M: Yes<br>S: Yes	A: Yes<br>R: Yes	None<br><br>DAS: Encouraged	PeerJ staff check for: competing interests declared, misconduct or integrity checks, ethical and legal compliance, funder acknowledgment	P: Permanent with some removal options in extraneous circumstances<br>R: False or inaccurate content, additional reasons not stated<br>W: Basic information remains on a tombstone page	Email administrator	S: Supported by associated journal publishing (PeerJ)<br>P: Preprints permanently archived in Portico and EuropePMC
Preprints with The Lancet [37] – *Verified*	M: Yes<br>S: Yes	A: Unknown<br>R: Unknown	Unknown	SSRN staff and a Lancet editor check for: competing interests	P: Unknown<br>R: Unknown	Unknown	S: Unknown<br>P: Unknown

				declared, ethical and legal compliance, funder acknowledgment, clinical trial registration, relevance of material	W: Unknown		
Preprints.org [38]	M: Yes<br>S: Yes	A: Yes<br>R: Yes	None as yet<br><br>DAS: Encouraged	Preprints staff and advisory board members (*including active researchers*) check for: plagiarism (as needed), authors are genuine, ethical and legal compliance, basic scientific content, data/code availability	P: Permanent with some removal options in extraneous circumstances<br>R: Contravention of copyright, plagiarism, false or inaccurate content, ethical and legal issues, misconduct<br>W: Basic information remains on a tombstone page if DOI has been issued, otherwise no web presence retained	Email administrator	S: Supported by associated journal publishing (MDPI)<br>P: Currently no long-term preservation strategy
Research Square [39] – *Verified*	M: Yes<br>S: Yes	A: Not yet<br>R: Not applicable	None as yet<br><br>DAS: Not requested or required	In-house editorial team checks for: competing interests declared, plagiarism (only for submissions to BMC journals), ethical and legal compliance, funder acknowledgment, clinical trial registration, relevance of material, basic scientific content, language, potential for harm	P: Permanent with some removal options in extraneous circumstances<br>R: Contravention of copyright, ethical and legal issues<br>W: Basic information remains on a tombstone page	Email administrator	S: Other business model not based on direct preprinting charges or associated journal publishing (publishing services by Research Square)<br>P: Preprints permanently archived in Portico
SciELO Preprints [40] – *Verified*	M: No<br>S: No	A: Yes<br>R: Yes	Yes, endorses, without stating on website<br><br>DAS: Not requested or required	SciELO editors (*including active researchers*) will check for: competing interests declared, plagiarism, misconduct and integrity checks, ethical and legal compliance, authors are genuine, all authors provide permission to post, clinical trial registration, funder acknowledgment, data/code availability	P: Permanent with some removal options in extraneous circumstances<br>R: Contravention of copyright, plagiarism, false or inaccurate content, ethical and legal issues<br>W: No web presence retained	Email administrator	S: Internal financial support (SciELO program)<br>P: Plan to use LOCKSS-compatible preservation strategy [once full operation launched]
SSRN [41] – *Verified*	M: No<br>S: No	A: Yes<br>R: Yes	Unknown	SSRN staff check for relevance of material	P: Content maybe removed by author (or operator/owner)<br>R: Contravention of copyright, plagiarism, false or inaccurate content, ethical and legal issues<br>W: Unknown	Email administrator	S: Unknown<br>P: Unknown
Surgery Open Science	M: No	A: Unknown	Unknown	Unknown	P: Unknown	Unknown	S: Unknown

- *First Look* [42] – *Verified*	S: No	R: Unknown			R: Unknown<br>W: Unknown		P: Unknown
Therapoid [43] – *Verified*	M: No<br>S: No	A: Yes<br>R: Yes	None as yet<br><br>DAS: Not requested or required	There is no pre-moderation of submissions; any checks are performed by site users (*including active researchers*) after posting	P: Permanent with some removal options in extraneous circumstances<br>R: Contravention of copyright, plagiarism, false or inaccurate content, ethical and legal issues<br>W: No web presence retained	Not yet in place: expect community moderation via forum	S: Unknown<br>P: All content is preserved – no further information reported by server
ViXra [44]	M: Yes<br>S: Yes	A: Yes<br>R: Yes	Mostly compliant, without endorsing, without stating on website<br><br>DAS: Not requested or required	Administrators do not perform formal screening checks but reject submissions in response to issues relating to: plagiarism, misconduct and integrity checks, ethical and legal compliance, format (PDF)	P: Content maybe removed by author (or operator/owner)<br>R: Contravention of copyright, plagiarism, false or inaccurate content, ethical and legal issues<br>W: No web presence retained	Email administrator	S: Sustainable low-cost web hosting service secured, previously run on donations<br>P: Long-term archiving provided by mirror site and cloud storage of database

Preprint platform websites

1. AfricArxiv https://info.africarxiv.org/
2. AgriXiv https://agrixiv.org
3. Arabixiv https://arabixiv.org/
4. EcoEvoRxiv https://ecoevorxiv.org
5. FocUS Archive https://osf.io/preprints/focusarchive/
6. Frenxiv https://frenxiv.org
7. INA-Rxiv https://osf.io/preprints/inarxiv
8. MarXiv https://marxiv.org
9. MetaArXiv https://osf.io/preprints/metaarxiv/
10. MindRxiv https://mindrxiv.org
11. NutriXiv https://osf.io/preprints/nutrixiv
12. OSF Preprints https://osf.io/preprints/
13. PaleorXiv https://paleorxiv.org
14. PsyArXiv https://psyarxiv.com
15. SocArXiv https://osf.io/preprints/socarxiv
16. SportRxiv https://osf.io/preprints/sportrxiv
17. Thesis Commons https://thesiscommons.org
18. AAS Open Research https://aasopenresearch.org/
19. AMRC Open Research https://amrcopenresearch.org/
20. Gates Open Research https://gatesopenresearch.org/
21. HRB Open Research https://hrbopenresearch.org/
22. MNI Open Research https://mniopenresearch.org/
23. Wellcome Open Research https://wellcomeopenresearch.org/
24. arXiv https://arxiv.org
25. Authorea https://www.authorea.com
26. bioRxiv https://www.biorxiv.org/
27. Cell Press Sneak Peek https://papers.ssrn.com/sol3/Jeljour_results.cfm?form_name=journalBrowse&journal_id=3184889
28. ChemRxiv https://chemrxiv.org
29. ChinaXiv http://chinaxiv.org
30. ESSOAr https://www.essoar.org
31. F1000 Research https://www.essoar.org
32. JMIR Preprints https://preprints.jmir.org/
33. medRxiv https://www.medrxiv.org
34. MitoFit Preprint Archives https://www.mitofit.org/index.php/MitoFit_Preprint_Archives
35. NeuroImage: Clinical – *First Look* https://papers.ssrn.com/sol3/JELJOUR_Results.cfm?form_name=journalBrowse&journal_id=3178959
36. PeerJ Preprints https://peerj.com/preprints/
37. Preprints with The Lancet https://papers.ssrn.com/sol3/JELJOUR_Results.cfm?form_name=journalBrowse&journal_id=3184962
38. Preprints.org https://www.preprints.org/
39. Research Square https://www.researchsquare.com
40. SciELO Preprints https://preprints.scielo.org/index.php/scielo
41. SSRN https://www.ssrn.com
42. Surgery Open Science – *First Look* https://papers.ssrn.com/sol3/Jeljour_results.cfm?form_name=journalBrowse&journal_id=3303309
43. Therapoid https://therapoid.net
44. ViXra http://vixra.org
