# Supplementary material for: Systematic examination of preprint platforms for use in the medical and biomedical sciences setting
Source: BMJ Open. 2020 Dec 29;10(12):e041849. doi: 10.1136/bmjopen-2020-041849 (PMC7778769; doi:10.1136/bmjopen-2020-041849)
Supplement: Supplementary data [file bmjopen-2020-041849supp005.pdf]

Supplementary Table 5: Usage metrics & other features

Preprint Server	Usage metrics on abstract page						Other features			
**OSF Communities**	Number of views	Number of downloads	Number of citations (e.g. Google Scholar)	Number of comments	Altmetrics	Social media interactions (from platform not Altmetric)	Commenting (including annotation plug-ins)	Onsite search	Alerts (e.g. RSS (Really Simple Syndication) feeds, email alerts)	Other
AfricArxiv [1] – *Verified*		⊗					⊗	⊗		
AgriXiv [2] – *Verified*		⊗					⊗	⊗		
Arabixiv [3] – *Verified*		⊗					⊗	⊗		
EcoEvoRxiv [4] – *Verified*		⊗						⊗		
FocUS Archive [5] – *Verified*		⊗						⊗		
Frenxiv [6]] – *Verified*		⊗					⊗	⊗		
INA-Rxiv [7]] – *Verified*		⊗					⊗	⊗		
MarXiv [8] *– note: no longer on the OSF – Verified*		⊗					⊗	⊗		
MetaArXiv [9] – *Verified*		⊗					⊗	⊗		
MindRxiv [10] – *Verified*		⊗					⊗	⊗		
NutriXiv [11] – *Verified*		⊗					⊗	⊗		
OSF Preprints [12] – *Verified*		⊗					⊗	⊗		
PaleorXiv [13] – *Verified*		⊗					⊗	⊗		
PsyArXiv [14] – *Verified*		⊗					⊗	⊗		
SocArxiv [15] – *Verified*		⊗					⊗	⊗	⊗	
SportRxiv [16] – *Verified*		⊗					⊗	⊗		
Thesis Commons [17] – *Verified*		⊗					⊗	⊗		
Open Research Central infrastructure										
AAS Open Research [18] – *Verified*	⊗	⊗	⊗		⊗		⊗	⊗		'Link to Google Scholar citations, Blog

										and gateways’
AMRC Open Research [19] – *Verified*	⊗	⊗	⊗		⊗		⊗	⊗		‘Link to Google Scholar citations’
Gates Open Research [20] – *Verified*	⊗	⊗	⊗		⊗		⊗	⊗		‘Link to Google Scholar citations, Gateways’
HRB Open Research [21] – *Verified*	⊗	⊗	⊗		⊗		⊗	⊗		‘Link to Google Scholar citations, Blog’
MNI Open Research [22] – *Verified*	⊗	⊗	⊗		⊗		⊗	⊗		‘Link to Google Scholar citations’
Wellcome Open Research [23] – *Verified*	⊗	⊗	⊗		⊗		⊗	⊗		‘Link to Google Scholar citations, Blog and gateways’
**Others**										
arXiv [24] – *Verified*								⊗	⊗	
Authorea [25] – *Verified*	⊗	⊗				⊗	⊗			‘Forking (copying an article that may or may not be authored by you), follow the article’
bioRxiv [26] – *Verified*	⊗	⊗		⊗	⊗	⊗	⊗	⊗	⊗	‘Channels, PREreview reviews linked, PreLights blog linked, Pingbacks to blog, PCI, biOverlay and Twitter thread embedded’
Cell Press Sneak Peek [27] – *Verified*	⊗	⊗								Unknown ‘other’
ChemRxiv [28]	⊗	⊗	⊗		⊗					Unknown ‘other’
ChinaXiv [29]	⊗	⊗					⊗		⊗	‘News, Author blacklist’
ESSOAr [30] – *Verified*		⊗			⊗			⊗	⊗	‘Plaudit, QR code, bookmarks, reference management tools’
F1000 Research [31] – *Verified*	⊗	⊗	⊗		⊗		⊗	⊗	⊗	‘Link to Google Scholar citations’
JMIR Preprints [32]						⊗	⊗	⊗		
medRxiv [33] – *Verified*	⊗	⊗		⊗	⊗	⊗	⊗	⊗	⊗	
MitoFit Preprint Archives [34]										None

Kirkham JJ, *et al. BMJ Open* 2020; 10:e041849. doi: 10.1136/bmjopen-2020-041849

NeuroImage: Clinical - *First Look* [35] – *Verified*	⊗	⊗								Unknown 'other'
PeerJ Preprints [36] – *Verified*	⊗	⊗		⊗		⊗	⊗	⊗	⊗	
Preprints with The Lancet [37] – *Verified*	⊗	⊗								Unknown 'other'
Preprints.org [38]	⊗	⊗		⊗	⊗		⊗	⊗	⊗	Embedded bookmark tabs for reading and reference management tools
Research Square [39] – *Verified*	⊗	⊗		⊗	⊗		⊗	⊗		Email alerts with comments
SciELO Preprints [40] – *Verified*	⊗	⊗			⊗	⊗ (when regular operation launches in 2020)	⊗ (when regular operation launches in 2020)	⊗ (when regular operation launches in 2020)		
SSRN [41] – *Verified*	⊗	⊗								Plum metrics
Surgery Open Science - *First Look* [42] – *Verified*	⊗	⊗						⊗	⊗ (SSRN subscription)	
Therapoid [43] – *Verified*	'metrics coming soon'						⊗	⊗	⊗	'Blockchain technology being implemented'
ViXra [44]	⊗						⊗		⊗	

Preprint platform websites

1. AfricArxiv https://info.africarxiv.org/
2. AgriXiv https://agrixiv.org
3. Arabixiv https://arabixiv.org/
4. EcoEvoRxiv https://ecoevorxiv.org
5. FocUS Archive https://osf.io/preprints/focusarchive/
6. Frenxiv https://frenxiv.org
7. INA-Rxiv https://osf.io/preprints/inarxiv
8. MarXiv https://marxiv.org
9. MetaArXiv https://osf.io/preprints/metaarxiv/
10. MindRxiv https://mindrxiv.org
11. NutriXiv https://osf.io/preprints/nutrixiv
12. OSF Preprints https://osf.io/preprints/
13. PaleorXiv https://paleorxiv.org
14. PsyArXiv https://psyarxiv.com
15. SocArXiv https://osf.io/preprints/socarxiv
16. SportRxiv https://osf.io/preprints/sportrxiv
17. Thesis Commons https://thesiscommons.org
18. AAS Open Research https://aasopenresearch.org/
19. AMRC Open Research https://amrcopenresearch.org/
20. Gates Open Research https://gatesopenresearch.org/
21. HRB Open Research https://hrbopenresearch.org/
22. MNI Open Research https://mniopenresearch.org/
23. Wellcome Open Research https://wellcomeopenresearch.org/
24. arXiv https://arxiv.org
25. Authorea https://www.authorea.com
26. bioRxiv https://www.biorxiv.org/
27. Cell Press Sneak Peek https://papers.ssrn.com/sol3/Jeljour_results.cfm?form_name=journalBrowse&journal_id=3184889
28. ChemRxiv https://chemrxiv.org
29. ChinaXiv http://chinaxiv.org
30. ESSOAr https://www.essoar.org
31. F1000 Research https://www.essoar.org
32. JMIR Preprints https://preprints.jmir.org/
33. medRxiv https://www.medrxiv.org
34. MitoFit Preprint Archives https://www.mitofit.org/index.php/MitoFit_Preprint_Archives
35. NeuroImage: Clinical – *First Look* https://papers.ssrn.com/sol3/JELJOUR_Results.cfm?form_name=journalBrowse&journal_id=3178959
36. PeerJ Preprints https://peerj.com/preprints/
37. Preprints with The Lancet https://papers.ssrn.com/sol3/JELJOUR_Results.cfm?form_name=journalBrowse&journal_id=3184962
38. Preprints.org https://www.preprints.org/
39. Research Square https://www.researchsquare.com
40. SciELO Preprints https://preprints.scielo.org/index.php/scielo
41. SSRN https://www.ssrn.com
42. Surgery Open Science – *First Look* https://papers.ssrn.com/sol3/Jeljour_results.cfm?form_name=journalBrowse&journal_id=3303309
43. Therapoid https://therapoid.net
44. ViXra http://vixra.org
