# Supplementary material for: Systematic examination of preprint platforms for use in the medical and biomedical sciences setting
Source: BMJ Open. 2020 Dec 29;10(12):e041849. doi: 10.1136/bmjopen-2020-041849 (PMC7778769; doi:10.1136/bmjopen-2020-041849)
Supplement: Supplementary data [file bmjopen-2020-041849supp006.pdf]

Table 5: Metadata contents

Preprint Server	Title	DOI or identifier	Publication or deposition date	Author name(s)	Author affiliation(s)	Funder acknowledge-ment(s)	Subject category	License	Abstract	Full-text content	References	Relational link to journal publication version (where it exists)	Other
**OSF Communities**													
AfricArxiv [1] – *Verified*	⊗	⊗	⊗	⊗	⊗		⊗	⊗	⊗			⊗	
AgriXiv [2] – *Verified*	⊗	⊗	⊗	⊗			⊗	⊗	⊗			⊗	
Arabixiv [3] – *Verified*	⊗	⊗	⊗	⊗			⊗	⊗	⊗			⊗	
EcoEvoRxiv [4] – *Verified*	⊗	⊗	⊗	⊗			⊗	⊗	⊗				
FocUS Archive [5] – *Verified*	⊗	⊗	⊗	⊗			⊗	⊗	⊗			⊗	
Frenxiv [6] – *Verified*	⊗	⊗	⊗	⊗			⊗	⊗	⊗			⊗	
INA-Rxiv [7] – *Verified*	⊗	⊗	⊗	⊗			⊗	⊗	⊗			⊗	
MarXiv [8] – *Verified*	⊗	⊗	⊗	⊗			⊗	⊗	⊗			⊗	
MetaArXiv [9]– *Verified*	⊗	⊗	⊗	⊗			⊗	⊗	⊗			⊗	
MindRxiv [10] – *Verified*	⊗	⊗	⊗	⊗			⊗	⊗	⊗			⊗	
NutriXiv [11] – *Verified*	⊗	⊗	⊗	⊗					⊗				
OSF Preprints [12] – *Verified*	⊗	⊗	⊗	⊗			⊗	⊗	⊗			⊗	
PaleorXiv [13] – *Verified*	⊗	⊗	⊗	⊗			⊗	⊗	⊗			⊗	
PsyArXiv [14] – *Verified*	⊗	⊗	⊗	⊗			⊗	⊗	⊗			⊗	
SocArxiv [15] – *Verified*	⊗	⊗	⊗	⊗			⊗	⊗	⊗			⊗	
SportRxiv [16] – *Verified*	⊗	⊗	⊗	⊗			⊗	⊗	⊗			⊗	
Thesis Commons [17] – *Verified*	⊗	⊗	⊗	⊗			⊗	⊗	⊗			⊗	
Open Research Central													

Kirkham JJ, *et al. BMJ Open* 2020; 10:e041849. doi: 10.1136/bmjopen-2020-041849

infrastructure													
AAS Open Research [18] – *Verified*	⊗	⊗	⊗	⊗	⊗	⊗	⊗		⊗	⊗			Competing interests, Referee (name, affiliation, report), Commenter (name, affiliation, comment text), Study type, Article type
AMRC Open Research [19] – *Verified*													
Gates Open Research [20] – *Verified*													
HRB Open Research [21] – *Verified*													
MNI Open Research [22] – *Verified*													
Wellcome Open Research [23] – *Verified*													
**Others**													
arXiv [24] – *Verified*	⊗	⊗	⊗	⊗					⊗			⊗	
Authorea [25] – *Verified*	⊗	⊗	⊗	⊗	⊗		⊗		⊗	⊗			Keywords
bioRxiv [26] – *Verified*	⊗	⊗	⊗	⊗	⊗		⊗	⊗	⊗	⊗	⊗	⊗	
Cell Press Sneak Peek[b] [27] – *Verified*													No information
ChemRxiv [28]	⊗	⊗	⊗	⊗	⊗	⊗	⊗	⊗	⊗	⊗	⊗		
ChinaXiv [29]	⊗	⊗	⊗	⊗			⊗		⊗				
ESSOAr [30] – *Verified*	⊗	⊗	⊗	⊗	⊗	⊗	⊗	⊗	⊗			⊗	ORCID, keywords
F1000 Research [31] – *Verified*	⊗	⊗	⊗	⊗	⊗	⊗	⊗		⊗	⊗			Competing interests, Article is a Faculty Review (yes/no), Referee (name, affiliation,

													report), Commenter (name, affiliation, comment text), Study type, Article type
JMIR Preprints [32]	⊗	⊗	⊗	⊗				⊗	⊗				Journal currently submitted to
medRxiv [33] – *Verified*	⊗	⊗	⊗	⊗	⊗	⊗	⊗	⊗	⊗		⊗	⊗	
MitoFit Preprint Archives [34]	⊗	⊗	⊗	⊗	⊗	⊗	⊗	⊗	⊗				ORCID if provided, Language, Version
NeuroImage: Clinical - *First Look*[b] [35] – *Verified*													No information
PeerJ Preprints [36] – *Verified*	⊗	⊗	⊗	⊗	⊗	⊗	⊗	⊗	⊗			⊗	
Preprints with The Lancet[b] [37] – *Verified*													No information
Preprints.org [38]	⊗	⊗	⊗	⊗				⊗	⊗			⊗	
Research Square [39] – *Verified*	⊗	⊗	⊗	⊗	⊗	⊗	⊗	⊗	⊗	⊗	⊗	⊗	
SciELO Preprints [40] – *Verified*	⊗		⊗	⊗	⊗		⊗	⊗	⊗				Language
SSRN [41] – *Verified*	⊗	⊗	⊗	⊗					⊗		⊗		
Surgery Open Science – *First Look*[b] [42] – *Verified*													No information
Therapoid [43] – *Verified*	⊗	⊗	⊗	⊗			⊗		⊗				"Anything else the author provides"
ViXra [44]	⊗	⊗	⊗	⊗					⊗				

BMJ Publishing Group Limited (BMJ) disclaims all liability and responsibility arising from any reliance placed on this supplemental material which has been supplied by the author(s)

Kirkham JJ, *et al. BMJ Open* 2020; 10:e041849. doi: 10.1136/bmjopen-2020-041849

Preprint platform websites

1. AfricArxiv https://info.africarxiv.org/
2. AgriXiv https://agrixiv.org
3. Arabixiv https://arabixiv.org/
4. EcoEvoRxiv https://ecoevorxiv.org
5. FocUS Archive https://osf.io/preprints/focusarchive/
6. Frenxiv https://frenxiv.org
7. INA-Rxiv https://osf.io/preprints/inarxiv
8. MarXiv https://marxiv.org
9. MetaArXiv https://osf.io/preprints/metaarxiv/
10. MindRxiv https://mindrxiv.org
11. NutriXiv https://osf.io/preprints/nutrixiv
12. OSF Preprints https://osf.io/preprints/
13. PaleorXiv https://paleorxiv.org
14. PsyArXiv https://psyarxiv.com
15. SocArXiv https://osf.io/preprints/socarxiv
16. SportRxiv https://osf.io/preprints/sportrxiv
17. Thesis Commons https://thesiscommons.org
18. AAS Open Research https://aasopenresearch.org/
19. AMRC Open Research https://amrcopenresearch.org/
20. Gates Open Research https://gatesopenresearch.org/
21. HRB Open Research https://hrbopenresearch.org/
22. MNI Open Research https://mniopenresearch.org/
23. Wellcome Open Research https://wellcomeopenresearch.org/
24. arXiv https://arxiv.org
25. Authorea https://www.authorea.com
26. bioRxiv https://www.biorxiv.org/
27. Cell Press Sneak Peek https://papers.ssrn.com/sol3/Jeljour_results.cfm?form_name=journalBrowse&journal_id=3184889
28. ChemRxiv https://chemrxiv.org
29. ChinaXiv http://chinaxiv.org
30. ESSOAr https://www.essoar.org
31. F1000 Research https://www.essoar.org
32. JMIR Preprints https://preprints.jmir.org/
33. medRxiv https://www.medrxiv.org
34. MitoFit Preprint Archives https://www.mitofit.org/index.php/MitoFit_Preprint_Archives
35. NeuroImage: Clinical – *First Look* https://papers.ssrn.com/sol3/JELJOUR_Results.cfm?form_name=journalBrowse&journal_id=3178959
36. PeerJ Preprints https://peerj.com/preprints/
37. Preprints with The Lancet https://papers.ssrn.com/sol3/JELJOUR_Results.cfm?form_name=journalBrowse&journal_id=3184962
38. Preprints.org https://www.preprints.org/
39. Research Square https://www.researchsquare.com
40. SciELO Preprints https://preprints.scielo.org/index.php/scielo
41. SSRN https://www.ssrn.com
42. Surgery Open Science – *First Look* https://papers.ssrn.com/sol3/Jeljour_results.cfm?form_name=journalBrowse&journal_id=3303309
43. Therapoid https://therapoid.net
44. ViXra http://vixra.org
