# Supplementary material for: Systematic examination of preprint platforms for use in the medical and biomedical sciences setting
Source: BMJ Open. 2020 Dec 29;10(12):e041849. doi: 10.1136/bmjopen-2020-041849 (PMC7778769; doi:10.1136/bmjopen-2020-041849)
Supplement: Author's manuscript [file bmjopen-2020-041849.draft_revisions.pdf]

BMJ Open is committed to open peer review. As part of this commitment we make the peer review history of every article we publish publicly available.

When an article is published we post the peer reviewers' comments and the authors' responses online. We also post the versions of the paper that were used during peer review. These are the versions that the peer review comments apply to.

The versions of the paper that follow are the versions that were submitted during the peer review process. They are not the versions of record or the final published versions. They should not be cited or distributed as the published version of this manuscript.

BMJ Open is an open access journal and the full, final, typeset and author-corrected version of record of the manuscript is available on our site with no access controls, subscription charges or pay-per-view fees (http://bmjopen.bmj.com).

If you have any questions on BMJ Open's open peer review process please email info.bmjopen@bmj.com

BMJ Open

A systematic examination of preprint platforms for use in the medical and biomedical sciences setting

Journal:	*BMJ Open*
Manuscript ID	bmjopen-2020-041849
Article Type:	Original research
Date Submitted by the Author:	18-Jun-2020
Complete List of Authors:	Kirkham, Jamie J.; Manchester University, Biostatistics<br>Penfold, Naomi; ASAPbio<br>Murphy, Fiona; Murphy Mitchell Consulting Ltd<br>Boutron, Isabelle; Center of Research in Epidemilogy and Statistics Sorbone Paris, CRESS UMR 1153, METHODS team,<br>Ioannidis, John; Stanford University, Stanford Prevention Research Center, Department of Medicine and Department of Health Research and Policy<br>Polka, Jessica; ASAPbio<br>Moher, David; Ottawa Hospital Research Institute, Ottawa Methods Centre
Keywords:	STATISTICS & RESEARCH METHODS, MEDICAL JOURNALISM, MEDICAL ETHICS

SCHOLARONE™
Manuscripts

I, the Submitting Author has the right to grant and does grant on behalf of all authors of the Work (as defined in the below author licence), an exclusive licence and/or a non-exclusive licence for contributions from authors who are: i) UK Crown employees; ii) where BMJ has agreed a CC-BY licence shall apply, and/or iii) in accordance with the terms applicable for US Federal Government officers or employees acting as part of their official duties; on a worldwide, perpetual, irrevocable, royalty-free basis to BMJ Publishing Group Ltd ("BMJ") its licensees and where the relevant Journal is co-owned by BMJ to the co-owners of the Journal, to publish the Work in this journal and any other BMJ products and to exploit all rights, as set out in our licence.

The Submitting Author accepts and understands that any supply made under these terms is made by BMJ to the Submitting Author unless you are acting as an employee on behalf of your employer or a postgraduate student of an affiliated institution which is paying any applicable article publishing charge ("APC") for Open Access articles. Where the Submitting Author wishes to make the Work available on an Open Access basis (and intends to pay the relevant APC), the terms of reuse of such Open Access shall be governed by a Creative Commons licence – details of these licences and which Creative Commons licence will apply to this Work are set out in our licence referred to above.

Other than as permitted in any relevant BMJ Author's Self Archiving Policies, I confirm this Work has not been accepted for publication elsewhere, is not being considered for publication elsewhere and does not duplicate material already published. I confirm all authors consent to publication of this Work and authorise the granting of this licence.

For peer review only

A systematic examination of preprint platforms for use in the medical and biomedical sciences setting

Jamie J Kirkham[1*], Naomi Penfold[2], Fiona Murphy[3], Isabelle Boutron[4], John PA Ioannidis[5], Jessica K Polka[2], David Moher[6,7]

[1]Centre for Biostatistics, Manchester Academic Health Science Centre, University of Manchester, Manchester, United Kingdom.

[2]ASAPbio, San Francisco, CA, USA.

[3]Murphy Mitchell Consulting Ltd.

[4]Université de Paris, Centre of Research in Epidemiology and Statistics (CRESS), Inserm, Paris, F-75004 France.

[5]Meta-Research Innovation Center at Stanford (METRICS) and Departments of Medicine, of Epidemiology and Population Health, of Biomedical Data Science, and of Statistics, Stanford University, Stanford, CA, USA.

[6]Centre for Journalology, Clinical Epidemiology Program, Ottawa Hospital Research Institute, Ottawa, Canada.

[7]School of Epidemiology and Public Health, Faculty of Medicine, University of Ottawa, Ottawa, Canada.

[*]Corresponding Author:

Professor Jamie Kirkham

Centre for Biostatistics
Faculty of Biology, Medicine and Health
The University of Manchester
Jean McFarlane Building
Oxford Road
Manchester, M13 9PL, UK

Email: jamie.kirkham@manchester.ac.uk
Tel: +44 (0)161 275 1135

For peer review only

ABSTRACT

Objectives

The objective of this review is to identify all preprint platforms with biomedical and medical scope and to compare and contrast the key characteristics and policies of these platforms.

Study Design and Setting

Preprint platforms that were launched up to 25th June 2019 and have a biomedical and medical scope according to MEDLINE's journal selection criteria were identified using existing lists, web-based searches and the expertise of both academic and non-academic publication scientists. A data extraction form was developed, pilot-tested and used to collect data from each preprint platform's webpage(s).

Results

A total of 44 preprint platforms were identified as having biomedical and medical scope, 17 (39%) were hosted by the Open Science Framework preprint infrastructure, six (14%) were provided by F1000 Research Ltd (the Open Research Central infrastructure) and 21 (48%) were other independent preprint platforms. Preprint platforms were either owned by non-profit academic groups, scientific societies or funding organisations (n=28; 64%), owned/partly owned by for-profit publishers or companies (n=14; 32%) or owned by individuals/small communities (n=2; 5%). Twenty-four (55%) preprint platforms accepted content from all scientific fields although some of these had restrictions relating to funding source, geographical region or an affiliated journal's remit. Thirty-three (75%) preprint platforms provided details about article screening (basic checks) and 14 (32%) of these actively involved researchers with context expertise in the screening process. Almost all preprint platforms allow submission to any peer-reviewed journal following publication, have a preservation plan for read-access, and most have a policy regarding reasons for retraction and the sustainability of the service.

Conclusion

A large number of preprint platforms exist for use in biomedical and medical sciences, all of which offer researchers an opportunity to rapidly disseminate their research findings onto an open-access public server, subject to scope and eligibility.

Keywords: Biomedicine, Medicine, Preprint, Preprint platform, Preprint server, Review, Scholarly Publishing

For peer review

Strengths and limitations of this study

- We developed robust methodology for systematically identifying relevant preprint platforms and involved platform owners/representatives wherever possible to verify data.
- We undertook an internal pilot of developing and testing out the data collection form in collaboration with a preprint platform owner and funders.
- For platforms that had a partner journal and without verification, it was sometimes unclear if the policy information related to the journal, preprint platform or both.
- We provide a searchable database as a valuable resource for researchers, funders and policymakers in the biomedical and medical science field to determine which preprint platforms are relevant to their research scope and which have the functionality and policies that they value most.
- We plan to update this searchable database periodically to include any new relevant preprint platforms and to amend any changes in policy.

For peer review only

Introduction

A preprint is an non-peer reviewed scientific manuscript that authors can upload to a public preprint platform and make available almost immediately without formal external peer review. Posting a preprint enables researchers to 'claim' priority of discovery of a research finding; this can be particularly useful for early-career researchers in a highly competitive research environment. Some preprint platforms provide digital object identifier (DOIs) for each included manuscript. This information can be included in grant applications. Indeed, progressive granting agencies are recommending applicants include preprints in their applications (e.g., National Institutes of Health (NIH, USA) [1] and in the UK, preprints are becoming recognised as eligible outputs in the Research Excellence Framework (REF) exercise which assesses institutional research performance [2].

Preprints have been widely used in the physical sciences since the early 1990s, and with the creation of the repository of electronic articles, arXiv, over 1.6 million preprints or accepted/published manuscripts have been deposited on this platform alone [3]. Since September 2003, arXiv has supported the sharing of quantitative biology preprints under the q-bio category. The use of preprints in biomedical sciences is increasing, leading to the formation of the scientist-driven initiative ASAPbio (Accelerating Science and Publication in biology) to promote their use [4]. A preprint platform dedicated to life-science-related research (bioRxiv) founded in 2013 has already attracted nearly 80,000 preprints [5]. This platform was set up to capture science manuscripts from all areas of biology, however, medRxiv was launched in June 2019 to provide a dedicated platform and processes for preprints in medicine and health related sciences [6] and it already hosts over 3400 preprints, becoming particularly popular with COVID-19. The Center for Open Science [7] has also developed web infrastructure for these new 'Rxiv' (pronounced "archive") services [8], while F1000 Research Ltd has provided instances of its post-publication peer review and publishing platform for use by several funders (e.g. Wellcome Trust) and research institutions to encourage preprint-first research publishing [9]. Recently, several large publishers (Springer Nature, Wiley, Elsevier) have developed, co-developed or acquired preprint platforms or services, and in April 2020, SciELO launched a preprint platform that works with Open Journal Systems [10]. Many other preprint platforms also support dissemination of biomedical and medical sciences within their broader multi-disciplinary platforms.

For peer review only

Given the increase in the use and profile of preprint platforms, it is increasingly important to identify how many such platforms exist and to understand how they operate in relation to policies and practices important for scientific publishing. With this aim in mind, we conduct a review to identify all preprint platforms that have biomedical and medical science scope and contrast them in terms of their unique characteristics and policies. We also provide a searchable repository of the platforms identified so that researchers, funders and policymakers have access to a structured approach for identifying preprint platforms that are relevant to their research area.

Terminology

We define a preprint according to the COPE (Committee of Publication Ethics) definition:

"*A preprint is a scholarly manuscript posted by the author(s) in an openly accessible platform, usually before or in parallel with the peer review process*." [11].

Any platform or server that hosts a collection of preprints will be referred to as a *preprint platform*. We use 'platform' instead of 'server' because, within this definition, we include both servers with no dedicated formal peer-review service and platforms where a manuscript has been submitted for peer review and is openly available to view *before* the peer review is formally complete.

Methods

Preprint platform identification

A preliminary list of potentially relevant preprint platforms was identified using Martyn Rittman's original list [12] and extended using a basic *Google* web-search using the search term '*preprint*' and the knowledge of the Steering Group (study authors) and Amy Riegelman, University of Minnesota, containing academic experts in publication science and innovators in the promotion of preprints. Additional preprint platforms that were launched up to 25th June 2019 were included if we became aware of them during assessment via our network or contact with platform owners.

Preprint platform selection

We included any preprint platform that has biomedical or medical scope according to MEDLINE's journal selection criteria [13]. Generally this covers: "[manuscripts] *predominantly devoted to reporting original investigations in the biomedical and health sciences, including*

For peer review only - http://bmjopen.bmj.com/site/about/guidelines.xhtml

research in the basic sciences; clinical trials of therapeutic agents; effectiveness of diagnostic or therapeutic techniques; or studies relating to the behavioural, epidemiological, or educational aspects of medicine."

We aimed to be over-inclusive such that preprint platforms that hosted work within the above MEDLINE definition of scope amongst a broader scope (such as "all physical, chemical and life sciences") were included. Eligibility of preprint platforms was arrived at in discussion with two authors (JJK and NP) and independently approved by the Steering Group. Preprint platforms were excluded for any of the following reasons: they were no longer active (as of June 25, 2019); they were print only or had no web-presence ('offline'); their primary function was classed as a general purpose repository with no specific preprint functionality.

Data extraction items

A data collection form was developed by the Steering Group which aimed to capture both preprint platform characteristics and policies. The form was pilot-tested with bioRxiv and revised accordingly following discussion with the platform owner. The final agreed data collection form is available online in Zenodo repository [14]. In brief, we extracted information on the preprints scope, ownership, governance, indexing and citation identifiers, submission formatting, visibility/versioning, article processing charges, publication timings, editorial board membership and for-profit or not-for-profit status. We also collected data on any checking/screening before preprint posting, open access/copyright and licensing options, sustainability and long-term preservation strategies, usage metrics and the simultaneous deposition policy relating to a manuscript submitted to a journal and the manuscript on the platform, and, if appropriate, policies about the deposition of accepted and published papers onto the platform.

Data extraction process

A single researcher (JJK, NP, FM) completed the data extraction for each platform using information found on the platform's website where content was directly accessible or found on associated webpages provided by the platform (for example, the 'About' pages for many OSF platforms linked to external websites provided by the platform operators). Verbatim text from the online search was recorded alongside any relevant web links. The completed data extraction form was then sent to the platform contacts (usually the platform owner), who were asked to check the data for completeness, fill in any missing fields and respond to any queries. On receiving the responses from the platforms, the researcher updated the data form, in some cases simplifying the text records into categorised information. These data were then returned to the platform to confirm

For peer review only

the data were accurate and as complete as possible, and these records were then recorded as 'verified by the platform representative/owner'.

If no contact with the platform was established, a second researcher (JJK, NP, FM) independently completed the data extraction using information found on the platform's website and consensus was reached on the final agreed responses following discussion. The completed data form was sent to the platforms informing them that the included information would be presented about their platform as 'unverified' data. The deadline for preprint platforms to approve any information and to confirm that all data could be shared publicly was 19th January 2020. Further datasets and records were updated with information provided up to 27th January 2020, and are available on the Zenodo repository [14].

Reporting of results

The preprint platform characteristics and policies were summarised descriptively and divided into preprint platforms (i) hosted on the Open Science Framework (OSF) Preprints infrastructure, (ii) provided by the Open Research Central infrastructure, and (iii) all other eligible platforms. Characteristics are presented as: (a) the scope and ownership of each platform; (b) content-specific characteristics and information relating to submission, journal transfer options, and external discoverability; (c) screening, moderation, and permanence of content; (d) usage metrics and other features; and (e) metadata.

Ethical approval: Not required. This is a descriptive study of publicly available information made available on websites. Data was confirmed by preprint platform owners/representatives using only email contacts available on those public websites.

Patient and public involvement: No patients were involved in setting the research question nor were they involved in the design, implementation and reporting of the study. There are no plans to involve patients in the dissemination of results.

Results

From all sources, ninety potentially eligible preprint platforms were identified for this review, although 46 were excluded based on scope (n=23), inactivity (n=13), no online presence (n=5) or were general repositories (n=5) (Figure 1). A list of excluded preprint platforms can be found in Supplementary Table 1. Of the 44 included preprint platforms, 17 were hosted by the OSF preprint infrastructure (although MarXiv is no longer part of the OSF family), six were provided

For peer review only

by the Open Research Central infrastructure and 21 were other independent preprint platforms (Figure 1). Of the 21 independent preprints platforms, four were First Look platforms (Cell Press Sneak Peek, Preprints with the Lancet, NeuroImage: Clinical, and Surgery Open Science). Whilst meeting the criteria for inclusion in this review, PeerJ Preprints decided to accept no new preprints after 30th September 2019. Thirty-eight (86%) of the 44 preprint platforms verified their own data. We present the data tables in this manuscript, though all tables and raw data are available in the Zenodo repository [14]. A searchable database of all the preprint platform information is also available (https://asapbio.org/preprint-servers).

Scope and ownership of preprint platforms (Table 1)

Twenty-eight platforms (64%) are owned by non-profit academic groups, scientific societies or funding organisations while two platforms are owned by individuals or small communities (Frenxiv and ViXra). Fourteen preprint platforms (32%) are affiliated or partly owned by for-profit publishers or companies; however, the preprint service part of their operation was declared as non-profit for three of these (Preprints.org, ESSOAr and MitoFit Preprint Archives). Of the preprint platforms associated with 'for-profit' status, only F1000 Research requires authors to pay an article processing charge.

Twenty-four (55%) preprint platforms accepted articles that covered multidisciplinary scope while 20 (45%) were discipline specific (e.g. PsyArXiv for psychological research). Despite the multidisciplinary scope, there were some further restrictions for some of the platforms, for example, there are five regional platforms (AfricArxiv, Arabixiv, Frenxiv, INA-Rxiv, ChinaXiv) aimed mostly at research being conducted in a specific geographical region. The Open Research Central platforms also only accept articles that are funded by certain funders (e.g. Wellcome Open Research platform only accepts research funded by the Wellcome Trust). Some preprint platforms also only allow articles that fit the remit of their affiliated journals (e.g. Cell Press Sneak Peek). Across all platforms, the median time that they have been active is 32 months (range 10 months, medRxiv to 28 years 8 months, arXiv). In that time, over 2.72 million preprints have been posted and in 2020, two platforms (Research Square and bioRxiv) have averaged more than 2,500 biomedical postings per month.

Submission, journal transfer options and external discoverability (Table 2)

Where the information is known, all preprint platforms support the English language, and all accept research articles (with the exception of Thesis Commons which accepts only theses).

For peer review only

Some platforms also accept other languages and other article types including research presentation slides and posters. Readers can access the full content of articles from all platforms with the exception of JMIR Preprints and some of the First Look platforms (Cell Press Sneak Peek, Preprints with the Lancet and Surgery Open Science) where reader registration is required. All platforms support PDF as the main viewing option, for some platforms this can be viewed in the browser while for others it requires a download. For all platforms, authors can submit articles using either a Word doc or as a PDF, with many platforms offering authors a choice of licensing, although where authors do not get a choice, the license required is commonly the CC-BY license.

In general, the Open Science Framework (OSF) and many of the other platforms allow authors to submit their articles to any journal although in some cases there is facilitated submission to certain journals, for example, for bioRxiv there is a host of direct transfer journal options. Authors submitting to F1000 Research, the Open Research platforms and all First Look platforms can only submit articles to journals associated with the platform. Where the information is available, all platforms with the exception of Therapoid and ViXra are externally indexed and most are commonly indexed on *Google Scholar*.

Screening, moderation, and permanence of content (Table 3)

Thirty-three (75%) preprint platforms provided some detail about article screening, while two (FocUS Archive and SocArxiv) do mention checks although the details of such checks are unknown. Therapoid does not perform any screening checks but relies on a moderation process by site users following article posting and ViXra does not perform screening checks but will retract articles in response to issues. Fourteen (32%) preprint platforms that perform screening checks actively involved researchers with content expertise in this process. The three most common screening checks performed related to scope of the article (e.g. scientific content, not spam, relevant material, language), plagiarism and legal/ethical/societal issues and compliance. Only three preprint platforms (Research Square, bioRxiv and medRxiv) check whether the content is dangerous to human health.

All F1000 platforms (inclusive of Open Research ones), MitoFit Preprint Archives, PeerJ Preprints and Preprints.org describe policies online in relation to NIH guidance for reporting preprints [15] with regards to plagiarism, competing interests, misconduct and all other hallmarks of reputable scholarly publishing. Some preprint platforms do have policies but fall

For peer review only

short of transparently making these policies visible online while some platforms have no policies. If content is withdrawn, some platforms ensure that the article retains a web presence (e.g. basic information on a tombstone page) although this was not standard across all platforms. Almost all platforms have a preservation plan (or are about to implement) for read access. Most commonly, platforms have set up an archiving agreement with Portico. Others have made their own arrangements: as a notable example, the OSF platforms are associated with a preservation fund provided by the Center for Open Science (COS) to maintain read access for over 50 years. In addition, most platforms have details on the sustainability of the service, for the OSF platforms this come from an external source (e.g. grants to support the COS framework), while for the Open Research Central infrastructure platforms this comes from article processing charges covered by the respective funding agencies. For some of the other platforms, funding is received from either internal or external sources or from other business model services (e.g. from associated journal publishing).

Usage metrics and other features (Table 4)

With the exception of arXiv and MitoFit Preprint Archives (Therapoid metrics arriving soon), all preprint platforms have some form of usage metrics, and apart from JMIR Preprints and ViXra all provide the number of article downloads on the abstract page. The OSF preprints are limited to downloads but the Open Research Central platforms also include the number of views, number of citations and altmetrics, whilst some of the independent platforms also include details of social media interactions direct from the platform (as opposed to the altmetric attention score). Most platforms (n=33; 75%) have some form of commenting and onsite search options (35; 80%), and some (mostly but not exclusively to the independent platforms) have alerts such as RSS feeds or email alerts.

Metadata (Table 5)

Forty (91%) of platforms provided information on metadata and all provide the manuscript title, publication date, abstract, and author names in the metadata. Nearly all of these with the exception of SciELO Preprints provide a DOI or other manuscript identifier as well. The majority also offer subject categories (n=34) and license information (n=26) but less than half include author affiliations (n=17) and funder acknowledgements (n=13). Eleven platforms (all six platforms under the Open Research Central infrastructure, Authorea, bioRxiv, ChemRxiv, F1000 Research, Research Square) offer full-text content, but only five include references in the

For peer review only

metadata. Half of the platforms (n=22) offer a relational link to the journal publication (if it exists) in the metadata.

Discussion

Forty-four preprint platforms were identified that considered biomedical and medical scope. This review characterises each of these preprint platforms such that authors can make a more informed choice about which ones might be relevant to their research field. Moreover, funders can use the data from this review to compare platforms if they wish to explicitly support and/or encourage their researchers to use certain platforms.

Preprint platforms are fast evolving and despite our cutoff of 25th June 2019, we are aware of new eligible preprint platforms that have been or are about to be launched after this date, for example Open Anthropology Research Repository (OARR) [16] and Cambridge Open Engage [17]. However, the recent advancements in the number of preprint platforms in this field has meant that one platform in this review (PeerJ Preprints) ceased to accept new preprints from the end of September 2019 to focus on their peer-reviewed journal activities [18]. Through our searchable database (https://asapbio.org/preprint-servers), we will endeavour to keep this information up-to-date.

Due to the lack of formal external peer review, preprint platforms that include medical content have been criticised as they may lack quality which can lead to errors in methods, results and interpretation, which subsequently has the potential to harm patients [19, 20]. This review has demonstrated the reality that many preprints do undergo some sensible checks before going online, in contrast to the perception that preprints are not reviewed at all. Research Square, bioRxiv and medRxiv check specifically if there is potential harm to the preprints' dissemination before peer review. Research Square also offers a transparent checklist to indicate the status of various quality assurance checks (not equivalent to scientific peer review) for each preprint.

Empirical evidence to support the use of editors and peer reviewers as a mechanism to ensure the quality of biomedical research is relatively weak [21, 22] although other studies have rendered peer review as being potentially useful [23, 24]. This review provides some justification that preprint platforms might be a reasonable option for researchers, especially given the time spent and associated cost of peer review [25]. In a recent survey of authors that have published with F1000 Research, 70% of respondents found the speed of publication to be important or very important [26]. In some scenarios, the time to deliver research findings may be as equally as

For peer review only

important as research quality, and may be critical to health care provision. A good example of this is the current outbreak of novel coronavirus, where much of the preliminary evidence has been made available through preprints at the time of the World Health Organisation declaring the epidemic a public health emergency [27]. The issue of preprints being available before peer review, and also the level of screening before a preprint is posted, has been particularly pertinent in this case. As an example, bioRxiv has rapidly adapted to ensure users appreciate there has not been any peer review of the COVID-19-related work presented on this platform. In light of COVID-19, people including the patients and the public might be interested in a quick and easy way to search across platforms. As a start at improving discoverability, Europe PMC aggregates preprints from several repositories and already nearly 3000 preprint articles with 'COVID-19' in the title are listed [28].

Strengths and limitations of the study

The strength of this study is that we developed robust methodology for systematically identifying relevant preprint platforms and involved platform owners/representatives wherever possible to verify data that was either unclear or not available on platform websites, and when this was not possible, a second researcher was involved in the data acquisition process. Systematically identifying web-based data that is not indexed in an academic bibliographic database is challenging [29], though the methods employed here are compatible with the principles of a systematic search: the methods are transparent and reproducible. This approach builds on the work by Martyn Rittman to produce an earlier list of preprint servers [12], the process behind which did not use systematic methods or involve platform owners as far as we are aware.

We undertook an internal pilot of developing and testing out the data collection form in collaboration with a preprint platform owner (John Inglis, bioRxiv, medRxiv) and ASAPbio staff and funders (promoters of preprint use) in order to ensure that the list of characteristics collected was both complete and relevant to different stakeholder groups including academics and funders. Many of the general policy information for some platforms was not well-reported or easy to find online and therefore an unexpected but positive by-product of this research is that several of these platforms have updated their webpages to improve the visibility and transparency of their policies in response to this research. Similarly, some platforms became aware of policy attributes that they had not previously considered and are now in the process of considering these for future implementation.

For peer review only

One limitation is that we focussed our attention on the 'main' preprint article although in some cases different policies existed for the supplementary material, e.g. acceptable formats and licensing options. This level of detail will be included in our searchable database. Another potential shortcoming was that some preprint platforms had a partner journal and without verification it was sometimes unclear if the policy information related to the journal, preprint platform or both. Finally, we defined preprint platforms as hosting work before peer review is formally complete and we acknowledge that some platforms included here also host content that has already been peer-reviewed and/or published in a journal (e.g. post-prints) [30]; this is unlikely to affect the interpretation of policies for pre-printed works discussed herein.

Implications for authors of biomedical and medical research

With the increase in the number of preprint platforms available in the biomedical and medical research field, authors have the option to make publicly available and gain some early ownership of their research findings with little or no cost to themselves. Moreover, with many preprints platforms there is little restriction with regards to authors later publishing their preprints in peer-reviewed journals of their choice. While we did not tabulate information on this specifically, it was noted that some platforms (notably OSF platforms) did recommend that authors check the SHERPA/RoMEO service for details of a journal's sharing policy. There is also some evidence that pre-printing an article first may even boost citation rates [31] due to increased attention from tweets, blogs and news articles than those articles published without a preprint. With many platforms carrying out suitable quality-control checks and having long-term preservation strategies, preprint platforms offer authors direct control of the dissemination of their research in a rapid, free and open environment. As well as primary research, preprints are also vital to users of research (systematic reviewers and decision makers). As an example, a living mapping systematic review of ongoing research into COVID-19 is currently being undertaken, and almost all included studies to date have been identified through preprint platforms [32].

Implications for preprint platforms

There has been a sharp rise in the number of preprints being published each month and it has been estimated (as of June 2019), preprints in biology represents approximately 2.4% of all biomedical publications [33]; and as of April 2020 there are already over 2.72 million preprints in the platforms that we evaluated. This review has summarised the key characteristics and policies of preprint platforms posting both medical and biomedical content although there is a

For peer review only

need for some of these platforms to update their policies and to make them more transparent online. As preprints are not formally validated through peer review, it is important to make it clear that their validity is less certain than for peer-reviewed articles (although even the latter may still not be valid). There is perhaps a growing need to standardise the checking process across platforms; such a process should not diminish the speed of publication (what authors value most about a preprint [22]). There is the temptation of making the checking process more rigorous, e.g. by including relevant researchers within the field as gatekeepers. However, this may slow down the process of making scientific work rapidly available and may promote groupthink, blocking innovative contrarian ideas to be circulated for public open review in the preprint platforms. Based on current checks, our review shows that most preprint platforms manage to post preprints within 48 hours and all within a week on average. Further challenges may arise on resources if the number of preprints continue to rise at a similar rate and the number of new platforms begins to plateau. And now, as several initiatives progress with work to build scientific review directly onto preprints (e.g. Peer Community in [34], Review Commons [35], PREreview [36]), it may become even more important to provide clarity about the level of checks a manuscript has already received and would need to receive to be considered "certified" by the scientific community. If anything, the wide public availability of preprints allows for far more extensive review by many reviewers, as opposed to the typical journal peer-review where only a couple of reviewers are involved. Our review identified 14 platforms linked to for-profit publishers and companies but only F1000 Research currently charges a small article processing charge to authors. With the increase in demand and resources needed to maintain preprint platforms, we should be mindful that article processing charges may change downstream meaning that platforms may have to charge authors; this would be unfortunate in light of the open science movement.

Conclusion

One outcome of this review has been to understand the various drivers behind the proliferation of preprint platforms for the life and biomedical sciences. While arXiv, bioRxiv, chemRxiv and medRxiv aim to provide dedicated servers for academics within each field they are dedicated to, several academic groups have offered alternative subject-specific or regional services in line with their own community's needs, such as sharing work in languages other than English, using the OSF infrastructure. A third provider of preprint platforms is industry stakeholders: as academic publishers providing or acquiring preprint services to support the content they receive as submissions to their journals, and as biotechnology or pharmaceutical companies looking to

For peer review only

support the sharing of relevant research content. Whether any platform becomes dominant may be influenced by the communities who adopt them, the influencers who promote them (funders and researchers who influence hiring and promotion decisions) and the financial sustainability underpinning them. We hope that enabling transparency into the processes and policies at each platform empowers the research community (including researchers, funders and others involved in the enterprise) to identify and support the platform(s) that help them to share research results most effectively.

Acknowledgments

We thank John Inglis (co-founder of bioR$_X$iv, medRxiv) for his advice on developing the data collection form and helpful comments on the manuscript. We also thank Robert Kiley, Geraldine Clement-Stoneham, Michael Parkin, Amy Riegelman, and Claire Yang for helpful feedback and conversations. We also would like to thank collectively the preprint platform owners and representatives who provided both data and verified information.

Contributors

JJK and DM jointly conceived the study and are the guarantors. JJK, DM and NP designed the study methods and developed the data collection form. All authors were involved in identifying eligible platforms. JJK, NP and FM were all involved in data extraction and JJK and NP did the analysis and prepared the data tables. JP developed the online searchable database. JJK prepared the initial manuscript. All authors were involved in the revision of this manuscript. All authors read and approved the final manuscript and are accountable for all aspects of the work, including the accuracy and integrity.

Funding

NP, FM and JKP received funding for ASAPbio preprint research from The Wellcome Trust, Chan Zuckerberg Initiative, Howard Hughes Medical Institute, Simons Foundation, Medical Research Council, and Canadian Institutes of Health Research.

Competing Interests

JKP is Executive Director of ASAPbio.

Data sharing statement

The data from this study are available in Zenodo (https://zenodo.org/record/3700874), which we will update periodically with a new version number as new platforms come online and policies of platforms currently identified change.

For peer review only

References

[1] *NIH enables investigators to include draft preprints in grant proposals* [ONLINE]. Available at: http://www.sciencemag.org/news/2017/03/nih-enables-investigators-include-draft-preprints-grant-proposals. [Accessed 8 April 2020].

[2] *REF 2021: Guidance on submissions* [ONLINE]. Available at: https://www.ref.ac.uk/media/1092/ref-2019_01-guidance-on-submissions.pdf. [Accessed 8 April 2020].

[3] *arXiv* [ONLINE]. Available at: https://arxiv.org/. [Accessed 8 April 2020].

[4] *ASAPbio* [ONLINE]. Available at: http://asapbio.org/preprint-info. [Accessed 8 April 2020].

[5] *bioRxiv* [ONLINE]. Available at: https://www.biorxiv.org/. [Accessed 20 April 2020].

[6] *medRxiv* [ONLINE]. Available at: https://www.medrxiv.org/. [Accessed 21 April 2020].

[7] *Center for Open Science* [ONLINE]. Available at: https://cos.io/. [Accessed 8 April 2020].

[8] *INLEXIO: The rising tide of preprint servers* [ONLINE]. Available at: https://www.inlexio.com/rising-tide-preprint-servers/. [Accessed 8 April 2020].

[9] *F1000 Research Ltd* [ONLINE]. Available at https://f1000research.com/. [Accessed 8 April 2020].

[10] *SciELO Preprints* [ONLINE]. Available at: https://preprints.scielo.org/index.php/scielo. [Accessed 8 April 2020].

[11] *Committee on Publication Ethics. Discussion document on preprints* [ONLINE]. Available at: https://publicationethics.org/files/u7140/COPE_Preprints_Mar18.pdf [Accessed 8 April 2020].

[12] *Martyn Rittman Research Preprints: Server List* [ONLINE]. Available at: https://docs.google.com/spreadsheets/d/17RgfuQcGJHKSsSJwZZn0oiXAnimZu2sZsWp8Z6ZaYYo/edit#gid=0 [Accessed 8 April 2020].

[13] *U.S National Library of Medicine: Factsheet Medline® Journal Selection* [ONLINE]. Available at: https://www.nlm.nih.gov/lstrc/jsel.html [Accessed 8 April 2020].

[14] *Zenodo: Practices and policies of preprint platforms for life and biomedical sciences* [ONLINE]. Available at: https://zenodo.org/record/3700874. [Accessed 8 April 2020].

[15] *Reporting Preprints and Other Interim Research Products* [ONLINE]. https://grants.nih.gov/grants/guide/notice-files/not-od-17-050.html [Accessed 8 April 2020].

[16] *Open Anthropology Research Repository* [ONLINE]. Available at: https://www.openanthroresearch.org/. [Accessed 8 April 2020].

For peer review only

[17] *Cambridge Open Engage* [ONLINE]. Available at: https://www.cambridge.org/engage/coe/public-dashboard. [Accessed 8 April 2020].

[18] *PeerJ blog: PeerJ Preprints to stop accepting new preprints Sep 30th 2019* [ONLINE]. Available at: https://peerj.com/blog/post/115284881747/peerj-preprints-to-stop-accepting-new-preprints-sep-30-2019/. [Accessed 8 April 2020].

[19] Annesley T, Scott M, Bastian H, Fonseca V, Ioannidis JPA, Keller MA et al. Biomedical journals and preprint services: friends or foes? *Clinical Chemistry* 2017; **63**(2): 453-458.

[20] Chalmers I and Glasziou P. Should there be greater use of preprint servers for publishing reports of biomedical science? *F1000Research* 2016; 5:272.

[21] Jefferson T, Rudin M, Brodney Folse S, Davidoff F. Editorial peer review for improving the quality of reports of biomedical studies. *Cochrane Database of Systematic Reviews* 2007, Issue 2. Art. No.: MR000016.

[22] Bruce R, Chauvin A, Trinquart L, Ravaud P, Boutron I. Impact of interventions to improve the quality of peer review of biomedical journals: a systematic review and meta-analysis. *BMC Medicine* 2016; **14**:85.

[23] Hopewell S, Collins GS, Boutron I, Yu L-M, Cook J, Shanyinde M et al. Impact of peer review on reports of randomised trials published in open peer review journals: retrospective before and after study. *BMJ* 2014; 349:g4145.

[24] Carneiro CFD, Queiroz VGS, Moulin TC, Carvalho CAM, Haas CB, Rayêe D, Henshall DE et al. Comparing quality of reporting between preprints and peer-reviewed articles in the biomedical literature. *BioRxiv* 2020 doi: https://doi.org/10.1101/581892.

[25] Kovanis M, Porcher R, Ravaud P, Trinquart L. The global burden of journal peer review in the Biomedical Literature: Strong Imbalance in the Collective Enterprise. *PLOS ONE* 2016; 11(11): e0166387.

[26] Kirkham J, Moher D. Who and why do researchers opt to publish in post-publication peer review platforms? - findings from a review and survey of F1000 Research. *F1000 Research* 2018, 7:920.

[27] *World Economic Forum: Coronavirus and the risks of 'speed science'* [ONLINE]. Available at: https://www.weforum.org/agenda/2020/03/speed-science-coronavirus-covid19-research-academic. [Accessed 14 April 2020].

[28] *Preprints in Europe PMC: reducing friction for discoverability* [ONLINE]. Available at: http://blog.europepmc.org/2018/07/preprints.html. [Accessed 14 April 2020].

[29] Stansfield C, Dickson K, Bangpan M. Exploring issues in the conduct of website searching and other online sources for systematic reviews: how can we be systematic? *Systematic Reviews* 2016; **5**:191.

[30] scholcommlab: Analyzing preprints: The challenges of working with OSF metadata. Available at: https://www.scholcommlab.ca/2019/09/11/preprints-challenges-part-two/ [Accessed 14 April 2020].

[31] *Nature Index: Preprints boost article citations and mentions* [ONLINE]. Available at: https://www.natureindex.com/news-blog/preprints-boost-article-citations-and-mentions [Accessed 14 April 2020].

[32] Living mapping and living systematic review of Covid-19 studies [ONLINE]. Available at: https://covid-nma.com/ [Accessed 14 April 2020].

[33] *ASAPbio: Biology preprints over time* [ONLINE]. Available at: https://asapbio.org/preprint-info/biology-preprints-over-time [Accessed 13 February 2020].

[34] *Peer Community in* [ONLINE]. Available at: https://peercommunityin.org/ [Accessed 14 April 2020].

[35] *Review COMMONS* [ONLINE]. Available at: https://www.reviewcommons.org/ [Accessed 14 April 2020].

[36] *PREREVIEW* [ONLINE]. Available at: https://content.prereview.org/ [Accessed 14 April 2020].

For peer review only

Figure 1: Flow diagram of included preprint platforms covering biomedical and medical scope

Table 1: Scope and ownership of preprint platforms

Table 2: Submission, journal transfer options and external discoverability

Table 3: Screening, moderation, and permanence of content

Table 4: Usage metrics and other features

Table 5: Metadata

S1_Table.docx: Reasons for excluded preprint platforms

Figure 1: Flow diagram of included preprint platforms covering biomedical and medical scope

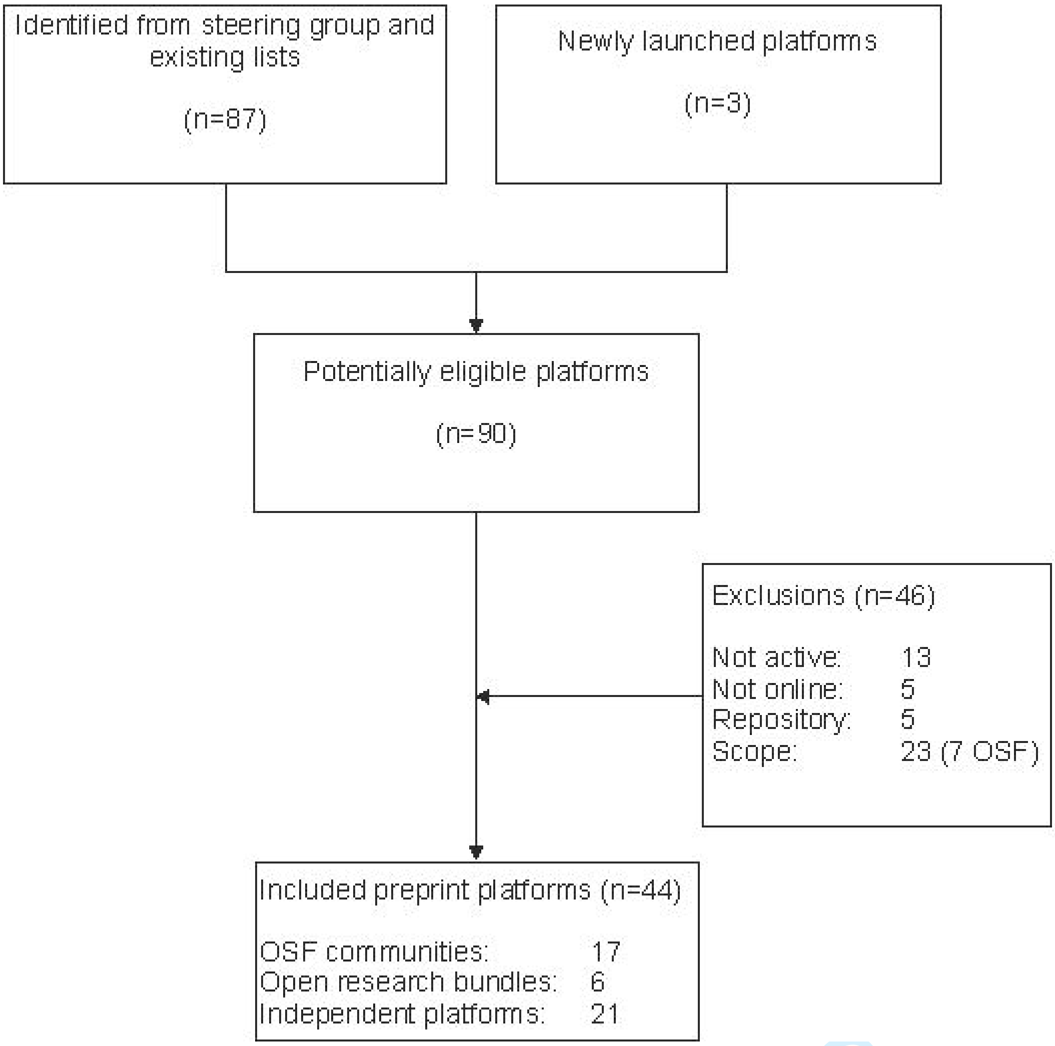

OSF: Open Science Framework

For peer review only - http://bmjopen.bmj.com/site/about/guidelines.xhtml

Table 1: General platform details

Preprint Server (date launched/content first posted; number of articles[a])	Platform Description	Scope	Ownership (O)/Ownership Type (OT)/For Profit status (P)/Technology (T; name & openness of source code)	Time to publication (T) / Processing Charges (C)
**OSF Communities**				
AfricArxiv [1]<br>(22 June 2018; 103)<br>*Verified*	"..is a free, open source and community-led digital archive for African research"	All scientific fields	O[b]: Small group of enthusiasts<br>OT: Academic community group; charity<br>P: Non-profit or not-for-profit<br>T: Open Science Framework (open source)	T: A few days<br>C: No fee to author
AgriXiv [2]<br>(15 February 2017; 418)<br>*Verified*	"Preprints for Agriculture and Allied Sciences"	Relating to agriculture and allied sciences, including life sciences, medicine and health sciences, social and behavioural sciences	O[b]: Open Access India and small group of enthusiasts<br>OT: Academic community group<br>P: Non-profit or not-for-profit<br>T: Open Science Framework (open source)	T: Unknown<br>C: No fee to author
Arabixiv [3]<br>(2 July 2018; 424)<br>*Verified*	"The Arabic Preprint Server"	All scientific fields	O[b]: Small group of enthusiasts<br>OT: Academic community group<br>P: Non-profit or not-for-profit<br>T: Open Science Framework (open source)	T: Up to 48 hours<br>C: No fee to author
EcoEvoRxiv [4]<br>(21 March 2018; 191)<br>*Verified*	"A free preprint service for ecology, evolution and conservation"	Subject-specific, including ecology, evolution and conservation	O[b]: Transparency in Ecology and Evolution<br>OT: Academic community group<br>P: Non-profit or not-for-profit<br>T: Open Science Framework (open source)	T: Up to 48 hours<br>C: No fee to author
FocUS Archive [5]<br>(20 September 2017; 42)<br>*Verified*	"A free preprint service for the focused ultrasound research community"	Relating to ultrasound, including life sciences, medicine and health sciences	O[b]: Focused Ultrasound Foundation<br>OT: Funding organisation (funder); medical research organisation<br>P: Non-profit or not-for-profit<br>T: Open Science Framework (open source)	T: Unknown<br>C: No fee to author
Frenxiv [6]<br>(06 July 2018; 94)<br>*Verified*	"The French server for Preprints in all the scientific fields"	All scientific fields	O[b]: Small group of enthusiasts<br>OT: Individual or community<br>P: Non-profit or not-for-profit<br>T: Open Science Framework (open source)	T: A few days<br>C: No fee to author
INA-Rxiv [7]<br>(17 August 2017; 16,637)<br>*Verified*	"A preprint server for Indonesian academia to provide an open, free and sustainable scientific repository"	All scientific fields	O[b]: Indonesia open science team<br>OT: Academic community group<br>P: Non-profit or not-for-profit<br>T: Open Science Framework (open source)	T: Unknown<br>C: No fee to author
MarXiv [8]<br>(09 November 2017; 449)<br>*note: no longer on the OSF*<br>*Verified*	"The free research repository for the ocean and marine-climate sciences"	Relating to ocean and marine climate sciences, including life sciences, social and behavioural sciences	O[b]: Open Communications for the Ocean (OCTO group)<br>OT: Publishing organisation<br>P: For-profit<br>T: Open Science Framework (open source)	T: Up to 48 hours<br>C: No fee to author
MetaArXiv [9]<br>(17 March 2017; 121)<br>*Verified*	"An interdisciplinary archive of articles focused on improving research transparency and reproducibility"	Relating to meta-science	O[b]: The Berkeley Initiative for Transparency in the Social Sciences (BITSS), Centre for Effective Global Action, University of California, Berkeley<br>OT: Academic institution<br>P: Non-profit or not-for-profit	T: Unknown<br>C: No fee to author

For peer review only

			T: Open Science Framework (open source)	
MindRxiv [10]<br>(17 July 2017; 192)<br>*Verified*	"Open archive for research on mind and contemplative practices"	Relating to mind and contemplative practices, including medicine and health sciences, neuroscience and neurobiology, psychology, social and behavioural sciences	O[b]: Mind and Life Institute<br>OT: Academic institution<br>P: Non-profit or not-for-profit<br>T: Open Science Framework (open source)	T: Same day<br>C: No fee to author
NutriXiv [11]<br>(11 September 2017; 63)<br>*Verified*	"A free preprint service for the nutritional sciences"	Relating to nutritional sciences	O[b]: Small group of enthusiasts<br>OT: Academic Institution<br>P: Non-profit or not-for-profit<br>T: Open Science Framework (open source)	T: Unknown<br>C: No fee to author
OSF Preprints [12]<br>(23 May 2007; 15,174)<br>*Verified*	"OSF Preprints is a preprints discovery site, where you can search not only the preprints in all subjects hosted at OSF Preprints, but also preprints on partner sites such as arXiv, AgriXiv, engrXiv, PsyArXiv and SocArXiv"	All scientific fields	O[b]: Center for Open Science<br>OT: Charity<br>P: Non-profit or not-for-profit<br>T: Open Science Framework (open source)	T: Unknown<br>C: No fee to author
PaleorXiv [13]<br>(18 August 2017; 162)<br>*Verified*	"…is a free, open source and community-led digital archive for Paleontology research"	Relating to palaeontology and paleobiology research	O[b]: Small group of enthusiasts<br>OT: Academic community group<br>P: Non-profit or not-for-profit<br>T: Open Science Framework (open source)	T: A few days<br>C: No fee to author
PsyArXiv [14]<br>(09 December 2016; 8,509)<br>*Verified*	"…is designed to facilitate rapid dissemination of psychological research"	Relating to psychology and psychological sciences	O[b]: Society for the Improvement of Psychological Science (SIPS)<br>OT: Scientific society<br>P: Non-profit or not-for-profit<br>T: Open Science Framework (open source)	T: A few days<br>C: No fee to author
SocArxiv [15]<br>(16 June 2017; 5,149)<br>*Verified*	"…open archive of the social sciences"	Relating to social sciences including social and behavioural sciences	O[b]: University of Maryland, College Park (UMD)<br>OT: Academic institution<br>P: Non-profit or not-for-profit<br>T: Open Science Framework (open source)	T: Unknown<br>C: No fee to author
SportRxiv [16]<br>(01 April 2017; 173)<br>*Verified*	"… is the first community-led and open access subject repository dedicated to sport, exercise, performance, and health research"	Relating to sports and exercise science, including rehabilitation and therapy, theatre, dance, physiology, physiotherapy, psychology, sociology	O[b]: Society for Transparency, Openness, and Replication in Kinesiology (STORK)<br>OT: Scientific society<br>P: Non-profit or not-for-profit<br>T: Open Science Framework (open source)	T: A few days<br>C: No fee to author
Thesis Commons [17]<br>(21 April 2017; 583)<br>*Verified*	"An open archive of theses"	All scientific fields	O[b]: Center for Open Science and small group of enthusiasts<br>OT: Academic community group; charity<br>P: Non-profit or not-for-profit<br>T: Open Science Framework (open source)	T: Unknown<br>C: No fee to author
**Open Research Central infrastructure**				
AAS Open Research [18]<br>(18 April 2018; 61)<br>*Verified*	"…is a platform for rapid publication and open peer review for researchers supported by AAS (African Academy of Sciences) and programs supported through its funding platform, AESA (Accelerating Excellence in Science in Africa's)"	Multiple scientific fields, including health and wellbeing[c]	O: African Academy of Sciences<br>OT: Funding organisation (funder)<br>P: Non-profit or not-for-profit<br>T: Custom F1000 software (closed source)	T: Up to 1 week<br>C: No fee to author (APC between £120-£800 typically paid by AAS)

For peer review only - http://bmjopen.bmj.com/site/about/guidelines.xhtml

AMRC Open Research [19]<br>(19 February 2019; 7)<br>*Verified*	"A platform for rapid author-led publication and open peer review of research funded by AMRC (Association of Medical Research Charities) member charities"	Broad life & biomedical research, including basic scientific, translational, applied and clinical research	O: Association of Medical Research Charities<br>OT: Funding organisation (funder), membership organisation<br>P: Non-profit or not-for-profit<br>T: Custom F1000 software (closed source)	T: Up to 1 week<br>C: Author fee applies (min £120; max £800[d]; £20 per poster, slide or document)
Gates Open Research [20]<br>(06 November 2017; 218)<br>*Verified*	"…is a platform for rapid author-led publication and open peer review of research funded by the Bill & Melinda Gates Foundation"	Global health, global development, global growth and opportunity	O: Bill and Melinda Gates Foundation<br>OT: Funding organisation (funder)<br>P: Non-profit or not-for-profit<br>T: Custom F1000 software (closed source)	T: Up to 1 week<br>C: No fee to author (APC between $150-$1500[d] paid by the Gates Foundation)
HRB Open Research [21]<br>(28 February 2018; 80)<br>*Verified*	"…is a platform for HRB-funded (Health Research Board) researchers to rapidly publish their research outputs in an open and accessible way"	Broad life & biomedical research, including exploratory, translational, biomedical, clinical, epidemiological, health services research	O: Health Research Board Ireland<br>OT: Funding organisation (funder)<br>P: Non-profit or not-for-profit<br>T: Custom F1000 software (closed source)	T: Up to 1 week<br>C: No fee to author (APC between €123-€818[d] paid by HRB)
MNI Open Research [22]<br>(05 December 2017; 11)<br>*Verified*	"…is a platform for rapid publication and open peer review of research conducted by MNI (Montreal Neurological Institute) and Hospital researchers"	Subject-specific, including brain imaging, cellular and molecular neuroscience, cognitive neuroscience, neurological diseases/disorders	O: Montreal Neurological Institute and Hospital, McGill University<br>OT: Academic institution<br>P: Non-profit or not-for-profit<br>T: Custom F1000 software (closed source)	T: Up to 1 week<br>C: No fee to author (APC between £116-£775[e] paid by MNI)
Wellcome Open Research [23]<br>(15 November 2016; 602)<br>*Verified*	"A new way for Wellcome-funded researchers to rapidly publish any results they think are worth sharing"	Broad life & biomedical research, including basic scientific, translational and clinical research	O: Wellcome Trust<br>OT: Funding organisation (funder)<br>P: Non-profit or not-for-profit<br>T: Custom F1000 software (closed source)	T: Up to 1 week<br>C: No fee to author (APC between £116-£775[d] paid by Wellcome)
**Others**				
arXiv [24]<br>(14 August 1991; 1,688,374)<br>*Verified*	"Open access to…e-prints…"	Multiple scientific fields, including quantitative biology[c]	O: Cornell University<br>OT: Academic institution<br>P: Non-profit or not-for-profit<br>T: SWORD (open source)	T: A few days<br>C: No fee to author
Authorea [25]<br>(06 August 2013; 2,579)<br>*Verified*	"…is the leading collaborative platform to read, write, and publish research"	All scientific fields	O: Atypon (Wiley)<br>OT: Publishing organisation (publisher)<br>P: For-profit<br>T: Custom Authorea software (proprietary software with some open source components)	T: Same day<br>C: No fee to author
bioRxiv [26]<br>(7 November 2013; 79,370)<br>*Verified*	"…is a free online archive and distribution service for unpublished preprints in the life sciences…"	Broad life & biomedical research (from animal behaviour and cognition to zoology)	O: Cold Spring Harbor Laboratory (CSHL)<br>OT: Academic institution<br>P: Non-profit or not-for-profit<br>T: Highwire Preprint & BenchPress (proprietary software with some open source components)	T: Up to 48 hours<br>C: No fee to author
Cell Press Sneak Peek [27]<br>(02 April 2018; 2,152)<br>*Verified*	"…is an author opt-in preview of the papers under review in [Cell Press] primary research journals"	Subject-specific, including cancer; stem cell; neuron; cell development; biology; immunity	O: Elsevier<br>OT: Publishing organisation (publisher)<br>P: For-profit<br>T: Unknown	T: A few days<br>C: No fee to author
ChemRxiv [28]<br>(15 August 2017; 4,332)	"…is a free submission, distribution and archive service for unpublished preprints in chemistry and related areas"	Subject-specific, including biological and medicinal chemistry	O: American Chemical Society, German Chemical Society, and Royal Society of Chemistry<br>OT: Scientific society<br>P: Non-profit or not-for-profit	T: Up to 48 hours<br>C: No fee to author

For peer review only

			T: Figshare (closed source)	
ChinaXiv [29]<br>(25 January 2016; 13,561)	"...is an open repository of Chinese scientific research papers in the field of natural sciences"	All scientific fields	O: Chinese Academy of Sciences<br>OT: Academic institution<br>P: Non-profit or not-for-profit<br>T: Java, SQL server (closed source)	T: Unknown<br>C: No fee to author
Earth and Space Science Open Archive (ESSOAr) [30]<br>(11 February 2018; 671)<br>*Verified*	"...is a community server established to accelerate the open discovery and dissemination of Earth and space science"	Relating to earth, environmental, and space sciences (subject-specific topics include biology, microbiology and public health)	O: American Geophysical Union (AGU), Wiley<br>OT: Scientific society; publishing organisation (publisher)<br>P: Non-profit or not-for-profit<br>T: Atypon/Literatum (closed source)	T: A few days<br>C: No fee to author
F1000 Research [31]<br>(13 July 2012; 3,226) – Verified	"...is an Open Research publishing platform for life scientists, offering immediate publication of articles and other research outputs without editorial bias"	Broad life & biomedical research, including basic scientific, translational and clinical research within the life sciences and medicine	O: F1000 Research Ltd.<br>OT: Publishing organisation (publisher)<br>P: For-profit<br>T: Custom F1000 software (closed source)	T: Up to 1 week<br>C: Author fee applies (min $150; max $2000 US)[d]
JMIR Preprints [32]<br>(11 November 2015; Not available)	"A preprint server for pre-publication/pre-peer-review preprints intended for community review as well as ahead-of-print (accepted) manuscripts"	Broad life & biomedical research	O: JMIR Publications Inc.<br>OT: Publishing organisation (publisher)<br>P: Unknown<br>T: Unknown	T: Unknown<br>C: No fee to author
medRxiv [33]<br>(25 June 2019; 3,412)<br>*Verified*	"...is a free online archive and distribution server for complete but unpublished manuscripts (preprints) in the medical, clinical, and related health sciences"	Broad medical, clinical and related health sciences	O: Cold Spring Harbor Laboratory (CSHL), Yale University and BMJ<br>OT: Academic institution<br>P: Non-profit or not-for-profit<br>T: Highwire Preprint & BenchPress (proprietary software with some open source components)	T: A few days<br>C: No fee to author
MitoFit Preprint Archives [34]<br>(12 February 2019; 15)	"Open Access preprint server for mitochondrial physiology and bioenergetics"	Subject-specific, including mitochondrial physiology, bioenergetics, ergodynamics	O: Oroborus Instruments<br>OT: Life sciences tech company<br>P: Non-profit or not-for-profit<br>T: MediaWiki (open source)	T: Up to 1 week<br>C: No fee to author
NeuroImage: Clinical - *First Look* [35]<br>(01 August 2018; 12)<br>*Verified*	"...is an author opt-in preview of the papers currently under consideration in [NeuroImage: Clinical]"	Subject-specific, including diseases, disorders and syndromes involving the nervous system, neuroimaging	O: Elsevier<br>OT: Publishing organisation (publisher)<br>P: For-profit<br>T: Unknown	T: A few days<br>C: No fee to author
PeerJ Preprints [36]<br>(04 April 2013, 5,068; *note: no new preprints accepted after 30 September 2019*)<br>*Verified*	"...is the 'pre-print' area of PeerJ"	Biological, medical and environmental sciences, matching scope to *PeerJ* journal (excludes Clinical Trials, diagnostic, therapeutic or health implications)	O: PeerJ<br>OT: Publishing organisation (publisher)<br>P: For-profit<br>T: Custom PeerJ Preprints software (closed source)	T: Up to 24 hours<br>C: No fee to author
Preprints with The Lancet [37]<br>(30 June 2018; 5,073)<br>*Verified*	"...a place where journals and other research experts identify content of interest prior to publication...Authors have either opted in at submission to The Lancet family of journals to post their preprints on Preprints with The Lancet, or submitted directly via SSRN"	Medical sciences	O: Elsevier<br>OT: Publishing organisation (publisher)<br>P: For-profit<br>T: Unknown	T: Up to 2 weeks<br>C: No fee to author

For peer review only

Preprints.org [38]<br>(02 July 2016; 14,837)	"…is a multidisciplinary preprint platform that makes scientific manuscripts from all fields of research immediately available"	All scientific fields	O: Multidisciplinary Digital Publishing Institute (MDPI)<br>OT: Publishing organisation (publisher)<br>P: Non-profit or not-for-profit<br>T: Custom software (closed source)	T: Up to 24 hours<br>C: No fee to author
Research Square [39]<br>(15 October 2018; 20,917)<br>*Verified*	"...lets you share your work early, gain feedback from the community, and start making changes to your paper prior to peer review in a journal"	All scientific fields	O: Research Square<br>OT: Publishing services organisation<br>P: For-profit<br>T: Custom software (closed source)	T: A few days<br>C: No fee to author
SciELO Preprints [40]<br>(06 July 2018; 31)<br>*Verified*	"…focus on manuscripts related to the main topics of the SciELO (Scientific Electronic Library Online) 20 Years Week agenda"	Multiple scientific fields including biological, health, human, applied social sciences[c]	O: SciELO and FAPESP Program<br>OT: Research infrastructure program<br>P: Non-profit or not-for-profit<br>T: Open Journals System (open source)	T: A few days<br>C: No fee to author
SSRN [41]<br>(1994; 791,714)<br>*Verified*	"…is an open-access online preprint community providing valuable services to leading academic schools and government institutions…"	Multiple scientific fields including applied sciences, health sciences, life sciences, social sciences[c]	O: Elsevier<br>OT: Publishing organisation (publisher)<br>P: For-profit<br>T: Unknown	T: Up to 48 hours<br>C: No fee to author
Surgery Open Science - *First Look* [42]<br>(23 April 2019; 42)<br>*Verified*	"…open access journal affiliated with the journal *Surgery* and striving to incorporate the strongest tenets of the open science movement including allowing author preprint"	Relating to surgery, matching scope to *Surgery* journal	O: Elsevier<br>OT: Publishing organisation (publisher)<br>P: Unknown<br>T: Unknown	T: A few days<br>C: No fee to author
Therapoid [43]<br>(08 February 2017; 9)<br>*Verified*	"an open web platform for scientific collaboration by Open Therapeutics"	Relating to pharmaceutical, medical device, and medical/health diagnostic technologies	O: Open Therapeutics<br>OT: Life sciences tech company<br>P: For-profit<br>T: Custom software (proprietary software with some open source components)	T: Same day<br>C: No fee to author
ViXra [44]<br>(25 February 2007; 35,405)	"An alternative archive… in Science and Mathematics serving the whole scientific community"	Multiple scientific fields, including biology[c]	O: Small group of enthusiasts<br>OT: Individual or community<br>P: Non-profit or not-for-profit<br>T: Custom viXra software with services from formsite.com and disqus.com (closed source)	T: Up to 48 hours<br>C: No fee to author

APC: Article Processing Charge

[a] As of 21st April 2020 (accept for SSRN, last verified 11th September 2019); [b] OSF communities are still working out the balance of ownership between service leads and the Centre for Open Science; [c] Disciplinary scope of server is broader – we list only those relevant to biomedical and health sciences; [d] Based on article length, surcharges may apply for articles with unusually large word counts; [e] MNI will pay for one publication per lab only.

For peer review only

Table 2: Scope & publishing practices: submission, viewing, discoverability & reuse

Preprint Server	Major content accepted (level of research[a], where known)	Content Language(s)	Permitted submission formats (S)<br>Viewing options (V)<br>Unique identifier (I; if DOI, prefix in brackets)<br>Versioning policy / version identifiers (VP)<br>Licensing options (L)<br>Reader registration required to view or download full content (R)	Journal submission options<br>Preprint displays link to journal publication (PL)	External content indexing (EI) / Metadata availability (MA) / Machine-readable full-text content (MR)
**OSF Communities**					
AfricArxiv [1] – *Verified*	Any scholarly content	Afrikaans, Akan, English, French, Igbo, Swahili, Zulu, other unspecified native African languages – summary must be provided in English and French, non-English languages held in moderation queue awaiting verification	S: PDF, Word doc, other text formats<br>V: By download in file format submitted by author(s), PDF viewable in browser without download<br>I: DOI (10.31730/osf.io)<br>VP: Accepts any new versions / single DOI retained for all versions<br>L: Authors are provided with a choice of licenses: GNU Lesser General Public License (LGPL) 3.0, BSD 3-Clause "New"/"Revised" License, BSD 2-Clause "Simplified" License, GNU Lesser General Public License (LGPL) 2.1, CC-By Attribution 4.0 International, Artistic License 2.0, CC0 1.0 Universal, Apache License 2.0, Mozilla Public License 2.0, Academic Free License (AFL) 3.0, Eclipse Public License 1.0, MIT License, GNU General Public License (GPL) 3.0, GNU General Public License (GPL) 2.0<br>R: No reader registration required	*Journal submission:* Unrestricted - manuscript can be published in any destination<br><br>*Journals directly associated with platform:* None<br><br>*Facilitated submission to other journals:* None<br><br>PL: Can be added by author	EI: Google Scholar, SHARE, Microsoft Academic, Unpaywall<br><br>MA: Openly available via API (application programming interface)<br><br>MR: No (planned in development roadmap)
AgriXiv [2] – *Verified*	Research manuscripts only	Cyrillic, English, Indonesian, unknown if others	S: Any format supported by OSF platform<br>V: By download in file format submitted by author(s), PDF viewable in browser without download<br>I: DOI (10.31220/osf.io)<br>VP: Accepts any new versions / single DOI retained for all versions<br>L: Authors are provided with a choice of licenses: CC0 1.0 Universal, CC-By Attribution 4.0 International<br>R: No reader registration required	*Journal submission:* Unrestricted - manuscript can be published in any destination<br><br>*Journals directly associated with platform:* None<br><br>*Facilitated submission to other journals:* None<br><br>PL: Can be added by author	EI: Google Scholar, SHARE, Microsoft Academic, Unpaywall<br><br>MA: Openly available via API<br><br>MR: No (planned in development roadmap)
Arabixiv [3] – *Verified*	Any scholarly content	Arabic, English	S: PDF<br>V: By download in file format submitted by author(s), PDF viewable in browser without download<br>I: DOI (10.31221/osf.io)<br>VP: Accepts any new versions / single DOI retained for all versions<br>L: Authors are provided with a choice of licenses: No license, GNU Lesser General Public License (LGPL) 3.0, BSD 3-Clause "New"/"Revised" License, BSD 2-Clause "Simplified" License, GNU Lesser General	*Journal submission:* Unrestricted - manuscript can be published in any destination<br><br>*Journals directly associated with platform:* None<br><br>*Facilitated submission to other journals:* None<br><br>PL: Can be added by author	EI: Google Scholar, SHARE, Microsoft Academic, Unpaywall<br><br>MA: Openly available via API<br><br>MR: No (planned in development roadmap)

For peer review only

			Public License (LGPL) 2.1, CC-By Attribution 4.0 International, Artistic License 2.0, CC0 1.0 Universal, Apache License 2.0, Mozilla Public License 2.0, Academic Free License (AFL) 3.0, Eclipse Public License 1.0, MIT License, GNU General Public License (GPL) 3.0, GNU General Public License (GPL) 2.0<br>R: No reader registration required		
EcoEvoRxiv [4] – *Verified*	All article types, datasets (primary and secondary research)	English	S: PDF<br>V: By download in file format submitted by author(s), PDF viewable in browser without download<br>I: DOI (10.32942/osf.io)<br>VP: Accepts any new versions / single DOI retained for all versions<br>L: Authors are provided with a choice of licenses: CC-By Attribution-ShareAlike 4.0 International, CC-By Attribution-NonCommercial-NoDerivatives 4.0 International, CC-By Attribution 4.0 International, CC0 1.0 Universal, CC-By Attribution-ShareAlike 4.0 International, CC-By Attribution-NonCommercial-NoDerivatives 4.0 International, with none preferred<br>R: No reader registration required	*Journal submission:* Unrestricted - manuscript can be published in any destination<br><br>*Journals directly associated with platform:* None<br><br>*Facilitated submission to other journals:* None<br><br>PL: Can be added by author	EI: Google Scholar, SHARE, Microsoft Academic, Unpaywall<br><br>MA: Openly available via API<br><br>MR: No (planned in development roadmap)
FocUS Archive [5] – *Verified*	Unknown	Unknown	S: Any format supported by OSF platform<br>V: By download in file format submitted by author(s), PDF viewable in browser without download<br>I: DOI (10.31225/osf.io)<br>VP: Accepts any new versions / single DOI retained for all versions<br>L: Authors must use CC-BY license<br>R: No reader registration required	*Journal submission:* Unrestricted - manuscript can be published in any destination<br><br>*Journals directly associated with platform:* None<br><br>*Facilitated submission to other journals:* None<br><br>PL: Can be added by author	EI: Google Scholar, SHARE, Microsoft Academic, Unpaywall<br><br>MA: Openly available via API<br><br>MR: No (planned in development roadmap)
Frenxiv [6] – *Verified*	All article types, datasets, posters (primary and secondary research)	Any language (including Arabic, English, French, Indonesian)	S: PDF, Word doc, LaTeX<br>V: By download in file format submitted by author(s), PDF viewable in browser without download<br>I: DOI (10.31226/osf.io)<br>VP: Accepts any new versions / single DOI retained for all versions<br>L: Authors are provided with a choice of licenses: GNU Lesser General Public License (LGPL) 3.0, BSD 3-Clause "New"/"Revised" License, BSD 2-Clause "Simplified" License, GNU Lesser General Public License (LGPL) 2.1, CC-By Attribution 4.0 International, Artistic License 2.0, CC0 1.0 Universal, Apache License 2.0, Mozilla Public License 2.0, Academic Free License (AFL) 3.0, Eclipse Public	*Journal submission:* Unrestricted - manuscript can be published in any destination<br><br>*Journals directly associated with platform:* None<br><br>*Facilitated submission to other journals:* None<br><br>PL: Can be added by author	EI: Google Scholar, SHARE, Microsoft Academic, Unpaywall<br><br>MA: Openly available via API<br><br>MR: No (planned in development roadmap)

For peer review only

			License 1.0, MIT License, GNU General Public License (GPL) 3.0, GNU General Public License (GPL) 2.0, No license<br>R: No reader registration required		
INA-Rxiv [7] – *Verified*	Multiple types (including research manuscripts, essays, datasets, multimedia files; types not accepted are: thesis, report, draft book)	English, Indonesian	S: PDF, Word doc, LaTeX, MarkDown<br>V: By download in file format submitted by author(s), PDF viewable in browser without download<br>I: DOI (10.31227/osf.io)<br>VP: Accepts any new versions / single DOI retained for all versions<br>L: Authors are provided with a choice of licenses: GNU Lesser General Public License (LGPL) 3.0, BSD 3-Clause "New"/"Revised" License, BSD 2-Clause "Simplified" License, GNU Lesser General Public License (LGPL) 2.1, CC-By Attribution 4.0 International, Artistic License 2.0, CC0 1.0 Universal, Apache License 2.0, Mozilla Public License 2.0, Academic Free License (AFL) 3.0, Eclipse Public License 1.0, MIT License, GNU General Public License (GPL) 3.0, GNU General Public License (GPL) 2.0, No license, with CC-BY preferred<br>R: No reader registration required	*Journal submission:* Unrestricted - manuscript can be published in any destination<br><br>*Journals directly associated with platform:* None<br><br>*Facilitated submission to other journals:* None<br><br>PL: Can be added by author	EI: Google Scholar, SHARE, Microsoft Academic, Unpaywall<br><br>MA: Openly available via API<br><br>MR: No (planned in development roadmap)
MarXiv [8] – *note: no longer on the OSF – Verified*	Any scholarly content	Unknown	S: PDF, LaTeX<br>V: By download in file format submitted by author(s), PDF viewable in browser without download<br>I: DOI (10.31230/osf.io)<br>VP: Accepts any new versions / single DOI retained for all versions<br>L: Authors are provided with a choice of licenses: CC-BY, CC-BY-NC-ND, No License, GNU Lesser General Public License (LGPL) 3.0, Eclipse Public License 1.0, CC-BY-SA; with CC-BY preferred<br>R: No reader registration required	*Journal submission:* Unrestricted - manuscript can be published in any destination<br><br>*Journals directly associated with platform:* None<br><br>*Facilitated submission to other journals:* None<br><br>PL: Can be added by author	EI: Google Scholar, SHARE, Microsoft Academic, Unpaywall, Altmetric, OpenChannels.org<br><br>MA: Openly available via API<br><br>MR: No (planned in development roadmap)
MetaArXiv [9] – *Verified*	Multiple types (including manuscripts, theses, conference proceedings, tutorials)	English	S: PDF, Word doc, WordPerfect, LaTeX<br>V: By download in file format submitted by author(s), PDF viewable in browser without download<br>I: DOI (10.31222/osf.io)<br>VP: Accepts any new versions / single DOI retained for all versions<br>L: Authors are provided with a choice of licenses: CC0 1.0 Universal, CC-By Attribution 4.0 International<br>R: No reader registration required	*Journal submission:* Unrestricted - manuscript can be published in any destination<br><br>*Journals directly associated with platform:* None<br><br>*Facilitated submission to other journals:* None<br><br>PL: Can be added by author	EI: Google Scholar, SHARE, Microsoft Academic, Unpaywall<br><br>MA: Openly available via API<br><br>MR: No (planned in development roadmap)
MindRxiv [10] – *Verified*	Research manuscripts only	English	S: Any format supported by OSF platform (PDF preferred)	*Journal submission:* Unrestricted - manuscript can be published in any destination	EI: Google Scholar, SHARE, Microsoft Academic, Unpaywall

For peer review only

			V: By download in file format submitted by author(s), PDF viewable in browser without download<br>I: DOI (10.31231/osf.io)<br>VP: Accepts any new versions / single DOI retained for all versions<br>L: Authors are provided with a choice of licenses: CC0, CC-BY, no license required; with CC0 preferred<br>R: No reader registration required	*Journals directly associated with platform:* None<br><br>*Facilitated submission to other journals:* None<br><br>PL: Can be added by author	MA: Openly available via API<br><br>MR: No (planned in development roadmap)
NutriXiv [11] – *Verified*	Unknown	Unknown	S: Any format supported by OSF platform<br>V: By download in file format submitted by author(s), PDF viewable in browser without download<br>I: DOI (10.31232/osf.io)<br>VP: Accepts any new versions / single DOI retained for all versions<br>L: Authors must use CC0 license<br>R: No reader registration required	*Journal submission:* Unrestricted - manuscript can be published in any destination<br><br>*Journals directly associated with platform:* None<br><br>*Facilitated submission to other journals:* None<br><br>PL: Can be added by author	EI: Google Scholar, SHARE, Microsoft Academic, Unpaywall<br><br>MA: Openly available via API<br><br>MR: No (planned in development roadmap)
OSF Preprints [12] – *Verified*	Multiple types (including research articles, posters, new data)	Any language	S: PDF, Word doc<br>V: By download in file format submitted by author(s), PDF viewable in browser without download<br>I: DOI (10.31219/osf.io)<br>VP: Accepts any new versions / single DOI retained for all versions<br>L: Authors are provided with a choice of licenses: CC0, CC-BY, no license; with no preference for which license chosen<br>R: No reader registration required	*Journal submission:* Unrestricted - manuscript can be published in any destination<br><br>*Journals directly associated with platform:* None<br><br>*Facilitated submission to other journals:* None<br><br>PL: Can be added by author	EI: Google Scholar, SHARE<br><br>MA: Openly available via API<br><br>MR: No (planned in development roadmap)
PaleorXiv [13] – *Verified*	Multiple article types (including systematic reviews, data papers, methods papers)	Any language – non-English languages held in moderation queue awaiting verification	S: PDF, LaTeX, text<br>V: By download in file format submitted by author(s), PDF viewable in browser without download<br>I: DOI (10.31233/osf.io)<br>VP: Accepts any new versions / single DOI retained for all versions<br>L: Authors must use CC-BY license<br>R: No reader registration required	*Journal submission:* Unrestricted - manuscript can be published in any destination<br><br>*Journals directly associated with platform:* None<br><br>*Facilitated submission to other journals:* None<br><br>PL: Can be added by author	EI: Google Scholar, SHARE, Microsoft Academic, Unpaywall<br><br>MA: Openly available via API<br><br>MR: No (planned in development roadmap)
PsyArXiv [14] – *Verified*	Any scholarly content	Unknown	S: PDF, Word doc<br>V: By download in file format submitted by author(s), PDF viewable in browser without download<br>I: DOI (10.31234/osf.io)<br>VP: Accepts any new versions / single DOI retained for all versions<br>L: Authors are provided with a choice of licenses: CC0, CC-BY, no license; with no preference stated<br>R: No reader registration required	*Journal submission:* Unrestricted - manuscript can be published in any destination<br><br>*Journals directly associated with platform:* None<br><br>*Facilitated submission to other journals:* None<br><br>PL: Can be added by author	EI: Google Scholar, SHARE, Microsoft Academic, Unpaywall<br><br>MA: Openly available via API<br><br>MR: No (planned in development roadmap)

For peer review only

SocArxiv [15] – *Verified*	Research manuscripts only	Unknown	S: Any format supported by OSF platform<br>V: By download in file format submitted by author(s), PDF viewable in browser without download<br>I: DOI (10.31235/osf.io)<br>VP: Accepts any new versions / single DOI retained for all versions<br>L: Authors are provided with a choice of licenses: CC0, CC-BY, no license; with CC0 or CC-BY preferred<br>R: No reader registration required	*Journal submission:* Unrestricted - manuscript can be published in any destination<br><br>*Journals directly associated with platform:* None<br><br>*Facilitated submission to other journals:* None<br><br>PL: Can be added by author	EI: Google Scholar, SHARE, Microsoft Academic, Unpaywall<br><br>MA: Openly available via API<br><br>MR: No (planned in development roadmap)
SportRxiv [16] – *Verified*	All article types (including research manuscripts, commentaries, review articles, current opinions, case studies, technical reports, short communications)	Any language – non-English languages held in moderation queue awaiting verification	S: PDF<br>V: By download in file format submitted by author(s), PDF viewable in browser without download<br>I: DOI (10.31236/osf.io)<br>VP: Accepts any new versions / single DOI retained for all versions<br>L: Authors are provided with a choice of licenses: CC0, CC-BY; with CC-BY preferred<br>R: No reader registration required	*Journal submission:* Unrestricted - manuscript can be published in any destination<br><br>*Journals directly associated with platform:* None<br><br>*Facilitated submission to other journals:* None<br><br>PL: Can be added by author	EI Google Scholar, SHARE, Microsoft Academic, Unpaywall<br><br>MA: Openly available via API<br><br>MR: No (planned in development roadmap)
Thesis Commons [17] – *Verified*	Theses only	Any language	S: PDF, Word doc<br>V: By download in file format submitted by author(s), PDF viewable in browser without download<br>I: DOI (10.31237/osf.io)<br>VP: Accepts any new versions / single DOI retained for all versions<br>L: Authors are provided with a choice of licenses: CC0, CC-BY; with no preference stated<br>R: No reader registration required	*Journal submission:* Unrestricted - manuscript can be published in any destination<br><br>*Journals directly associated with platform:* None<br><br>*Facilitated submission to other journals:* None<br><br>PL: Can be added by author	EI: Google Scholar, SHARE, Microsoft Academic, Unpaywall<br><br>MA: Openly available via API<br><br>MR: No (planned in development roadmap)
**Open Research Central infrastructure**					
AAS Open Research [18] – *Verified*<br><br>AMRC Open Research [19] – *Verified*<br><br>Gates Open Research [20] – *Verified*<br><br>HRB Open Research [21] – *Verified*<br><br>MNI Open Research [22] – *Verified*	All article types (primary and secondary research)<br><br>Editorials (Wellcome Open Research only)<br><br>Posters and slides (AAS, AMRC, Gates Open Research only)	English (articles)<br><br>Accepts posters and slides in all languages – metadata and description must be in English	S: Word doc, RTF (Wellcome Open Research also supports LaTeX via Overleaf template)<br>V: By PDF download, full-text viewable in browser without PDF (e.g. as HTML), by XML download<br>I: DOI (10.12688/aasopenres; …/amrcopenres; …/gatesopenres; …/hrbopenres; …/mniopenres; …/wellcomeopenres)<br>VP: Accepts any new versions / each version receives its own DOI and own non-DOI citable identifier<br>L: Authors must use CC-BY license<br>R: No reader registration required	*Journal submission:* Restricted - manuscript can only be published in journal linked to server (AAS/AMRC/Gates/HRB/MNI/Wellcome Open Research, respectively)<br><br>PL: Yes, added by platform within one month of publication	EI: Google Scholar, PrePubMed, Europe PMC, SciLit<br><br>MA: Available for free upon request (AAS/AMRC/Gates/HRB/MNI); Openly available via API (Wellcome Open Research)<br><br>MR: Yes (XML and HTML)

For peer review only

Wellcome Open Research [23] – *Verified*					
**Others**					
arXiv [24] – *Verified*	Research manuscripts only	Any language – abstract must be in English	S: PDF, LaTeX, HTML<br>V: PDF (and other format(s) as supplied by author) viewable in browser without download<br>I: Platform-specific ID (arXiv ID)<br>VP: Accepts any new versions / each version receives its own non-DOI citable identifier<br>L: Authors are provided with a choice of licenses: CC0, CC-BY, CC-BY-SA 4.0, CC-BY-NC-SA 4.0, non-exclusive license to distribute, any other CC license as specified in manuscript text; with no preference for which license chosen<br>R: No reader registration required	*Journal submission:* Unrestricted - manuscript can be published in any destination<br><br>*Journals directly associated with platform:* None<br><br>*Facilitated submission to other journals:* Not available<br><br>PL: Yes, added by platform after one month from publication	EI: Google Scholar, PrePubMed (q-bio only), Europe PMC, SciLit, SHARE, INSPIRE-HEP, The NASA Astrophysics Data System (ADS), The arXiv Search Interface from the National Science Library, Chinese Academy of Sciences (also in Chinese)<br><br>MA: Openly available via API<br><br>MR: Require full-text to be submitted in machine-readable format
Authorea [25] – *Verified*	Any scholarly content (including articles, datasets, code, figures, tables, slides, micropublications, and Jupyter Notebooks)	Any language	S: PDF, Word doc, LaTeX<br>V: Full-text viewable in browser without PDF (e.g. as HTML), by LaTeX download<br>I: DOI (10.22541)<br>VP: Accepts any new versions / each version receives its own DOI<br>L: Authors must use CC-BY license<br>R: No reader registration required	*Journal submission:* Unrestricted - manuscript can be published in any destination<br><br>*Journals directly associated with platform*: None<br><br>*Facilitated submission to other journals:* Yes, listed at https://support.authorea.com/en-us/article/how-do-i-directly-submit-to-a-publisher-atxzf7/<br><br>PL: Yes, added by platform within one month of publication	EI: SciLit, Crossref<br><br>MA: Unknown<br><br>MR: Yes (HTML)
bioRxiv [26] – *Verified*	Research manuscripts only (primary research only; white papers only; white papers accepted in Scientific Communication and Education category only; types not accepted are: datasets, theses, figures, protocols, correspondence	English – non-English language translations can be included as supplementary files	S: PDF, Word doc, WordPerfect<br>V: By PDF download, PDF viewable in browser without download, full-text viewable in browser without PDF (e.g. as HTML)<br>I: DOI (10.1101)<br>VP: Accepts new versions until acceptance at journal / single DOI retained but new versions have distinct URLs<br>L: Authors are provided with a choice of licenses: CC0, CC-BY, CC-BY-NC, CC-BY-ND, CC-BY-NC-ND, or no reuse; with no preference for which license chosen<br>R: No reader registration required	*Journal submission:* Unrestricted - manuscript can be published in any destination<br><br>*Journals directly associated with platform:* None<br><br>*Facilitated submission to other journals:* Yes, listed at https://www.biorxiv.org/about-biorxiv<br><br>PL: Yes, added by platform within one month of publication	EI: Google Scholar, Microsoft Academic, Meta, EuropePMC, SHARE, Crossref<br><br>MA: Openly available via API<br><br>MR: Yes (HTML)

For peer review only

	without new data or analyses)				
Cell Press Sneak Peek[b] [27] – *Verified*	Content criteria as directed by associated journal(s) (Cell Press portfolio)	Unknown	S: PDF<br>V: By PDF download, PDF viewable in browser without download<br>I: URL only<br>VP: Unknown / Unknown<br>L: Unknown<br>R: Reader can view basic metadata without account but must register to view and download full content	*Journal submission:* Manuscripts must be submitted to a Cell Press Journal first, authors are then provided with the option of displaying their manuscript while under review using SSRN's 'First Look' (aka 'Sneak Peek')<br><br>PL: Unknown	EI: Unknown<br><br>MA: Unknown<br><br>MR: No
ChemRxiv [28]	Research manuscripts only	English	S: Any (PDF)<br>V: By PDF download, PDF viewable in browser without download<br>I: DOI (10.26434)<br>VP: Accepts new versions until acceptance at journal / each version receives its own DOI suffix<br>L: Authors are provided with a choice of licenses: CC-BY, CC-BY-NC, CC-BY-NC-ND; with CC-BY-NC-ND preferred<br>R: No reader registration required	*Journal submission:* Unrestricted - manuscript can be published in any destination<br><br>*Journals directly associated with platform:* None<br><br>*Facilitated submission to other journals:* Yes, journals owned by American Chemical Society, Royal Society of Chemistry, German Chemical Society (other publishers coming soon)<br><br>PL: Yes, added by platform within one month of publication	EI: Google Scholar, EuropePMC, Chemical Abstracts Services, Crossref<br><br>MA: Openly available via API<br><br>MR: If provided by author in machine-readable format
ChinaXiv [29]	Research manuscripts only	Standard Chinese, English, unknown if other	S: PDF, Word doc, LaTeX<br>V: By PDF download<br>I: DOI (10.12074)<br>VP: Accepts any new versions / single DOI retained for all versions<br>L: Authors are provided with a choice of licenses: CC0, CC-BY, CC-BY-SA, CC-BY-NC-SA; with no preference for which license chosen<br>R: No reader registration required	*Journal submission:* Unrestricted - manuscript can be published in any destination<br><br>*Journals directly associated with platform:* Multiple, listed at http://chinaxiv.org/journal/browse.htm<br><br>*Facilitated submission to other journals:* Not available<br><br>PL: Can be added by author or journal	EI: ROAR (Registry of Open Access Repositories)<br><br>MA: Openly available via API<br><br>MR: No
ESSOAr [30] – *Verified*	Scholarly research content (including manuscripts and posters)	English	S: PDF (Word and LaTeX in development)<br>V: By PDF download<br>I: DOI (10.1002)<br>VP: Accepts new versions until acceptance at journal / each version receives its own DOI suffix<br>L: Authors are provided with a choice of licenses: CC-BY, CC-BY-NC, CC-BY-NC-ND, non-exclusive license; with no preference stated<br>R: No reader registration required	*Journal submission:* Unrestricted - manuscript can be published in any destination<br><br>*Journals directly associated with platform:* 21 AGU publications (others in roadmap)<br><br>*Facilitated submission to other journals:* Not yet (on roadmap)<br><br>PL: Yes, added by platform within one month of publication; can be added by author or journal	EI: Google Scholar, SHARE, Crossref<br><br>MA: Openly available via Crossref<br><br>MR: Yes

For peer review only

F1000 Research [31] – *Verified*	Any scholarly content (including articles, posters and slides; primary and secondary research)	English	S: Word doc, RTF, LaTeX via Overleaf only<br>V: By PDF download, full-text viewable in browser without PDF, by XML download<br>I: DOI (10.12688/f1000research)<br>VP: Accepts any new versions / each version receives its own DOI and own non-DOI citable identifier<br>L: Authors must use CC-BY license<br>R: No reader registration required	*Journal submission:* Restricted - manuscript can only be published in journal linked to server (F1000 Research)<br><br>PL: Yes, added by platform within one month of publication	EI: Google Scholar, Europe PMC, SHARE, SciLit, PrePubMed<br><br>MA: Openly available via API<br><br>MR: Yes (XML and HTML)
JMIR Preprints [32]	Research manuscripts, blog posts, "grey" online reports	Unknown	S: Word doc<br>V: By PDF download, PDF viewable in browser without download<br>I: DOI (10.2196/preprints.)<br>VP: Accepts first submission only / not applicable<br>L: Authors must use no license (all rights reserved)<br>R: Author choice: No reader registration required, or reader can view basic content (title, abstract) without account but must register to view and download full content	*Journal submission:* Unrestricted - manuscript can be published in any destination<br><br>*Journals directly associated with platform:* JMIR Publications journals<br><br>*Facilitated submission to other journals:* Unknown<br><br>PL: Unknown	EI: Unknown<br><br>MA: Unknown<br><br>MR: No
medRxiv [33] – *Verified*	Multiple article types (including research articles, systematic reviews and meta-analysis, clinical research design protocols, data articles; types not accepted are: theses, opinion pieces, editorials, narrative reviews, datasets)	English	S: PDF, Word doc, WordPerfect<br>V: By PDF download, PDF viewable in browser without download<br>I: DOI (10.1101)<br>VP: Accepts new versions until acceptance at journal / single DOI retained for all versions<br>L: Authors are provided with a choice of licenses: CC0, CC-BY, CC-BY-NC, CC-BY-ND, CC-BY-NC-ND, or no reuse; with no preference for which license chosen<br>R: No reader registration required	*Journal submission:* Unrestricted - manuscript can be published in any destination<br><br>*Journals directly associated with platform:* None<br><br>*Facilitated submission to other journals:* Yes, listed at https://www.medrxiv.org/content/about-medrxiv<br><br>PL: Yes, added by platform within one month of publication	EI: Google Scholar, Microsoft Academic Search, Crossref<br><br>MA: Openly available via Crossref<br><br>MR: No
MitoFit Preprint Archives [34]	Any scholarly content (primary and secondary research)	English	S: PDF (abstract to be submitted as Word doc)<br>V: By PDF download<br>I: DOI (10.26124)<br>VP: Accepts new versions until acceptance at journal / each version receives its own DOI suffix<br>L: Authors must use CC-BY-NC-ND license<br>R: No reader registration required	*Journal submission:* Unrestricted - manuscript can be published in any destination<br><br>*Journals directly associated with platform:* None<br><br>*Facilitated submission to other journals:* Unknown<br><br>PL: Yes, added by platform within one month of publication	EI: Google Scholar<br><br>MA: Openly available via DataCite<br><br>MR: No
NeuroImage: Clinical - *First Look*[b] [35]	Content criteria as directed by associated journal	Unknown	S: PDF, Word doc<br>V: By PDF download, PDF viewable in browser without download<br>I: URL only	*Journal submission:* Manuscripts must be submitted to the NeuroImage: Clinical first, authors are then provided with the option of	EI: Unknown<br><br>MA: Unknown

For peer review only

	(NeuroImage: Clinical)		VP: Unknown / Unknown<br>L: Unknown<br>R: No reader registration required	displaying their manuscript while under review using SSRN's 'First Look'<br><br>PL: Unknown	MR: No
PeerJ Preprints [36] – *Verified*	Any scholarly content (primary and secondary research)	English	S: PDF<br>V: By PDF download, PDF viewable in browser without download, by XML download<br>I: DOI (10.7287)<br>VP: Accepts any new versions until publication in a journal / each version receives its own non-DOI citable identifier<br>L: Authors are provided with a choice of licenses: CC0, CC-BY; with CC-BY preferred<br>R: No reader registration required	*Journal submission:* Unrestricted - manuscript can be published in any destination<br><br>*Journals directly associated with platform:* PeerJ journals<br><br>*Facilitated submission to other journals:* Unknown<br><br>PL: Can be added by author or journal	EI: Google Scholar, Europe PMC<br><br>MA: Openly available via Crossref<br><br>MR: No
Preprints with The Lancet[b] [37] – *Verified*	Content criteria as directed by associated journal(s) (Lancet family of journals)	Unknown	S: Unknown<br>V: By PDF download, PDF viewable in browser without download<br>I: URL only<br>VP: Accepts first submission only / not applicable<br>L: Unknown<br>R: Reader can view basic content (title, abstract) without account but must register to view and download full content	*Journal submission:* Manuscripts must be submitted to the Lancet family of journals (including EBioMedicine and EClinicalMedicine) first, authors are then provided with the option of displaying their manuscript while under review using SSRN's 'First Look'<br><br>PL: Can be added by author or journal	EI: Unknown<br><br>MA: Unknown<br><br>MR: No
Preprints.org [38]	Multiple article types (including research manuscripts, comprehensive reviews, data articles, case reports; types not accepted are: editorials, discussion papers, commentaries, coursework)	English	S: Word doc, LaTeX<br>V: By PDF download<br>I: DOI (10.20944)<br>VP: Accepts any new versions / each version receives its own DOI and own non-DOI citable identifier<br>L: Authors must use CC-BY license<br>R: No reader registration required	*Journal submission:* Unrestricted - manuscript can be published in any destination<br><br>*Journals directly associated with platform:* None<br><br>*Facilitated submission to other journals:* Not available<br><br>PL: Unknown	EI: Google Scholar, PrePubMed, Europe PMC, SHARE, SciLit<br><br>MA: Openly available via Crossref<br><br>MR: No
Research Square [39] – *Verified*	Multiple article types (including research articles, systematic reviews, method articles, data articles; types not accepted are:	English	S: Word doc, LaTeX<br>V: By PDF download, full-text viewable in browser without PDF (e.g. as HTML)<br>I: DOI (10.21203)<br>VP: Accepts any new versions / each version receives its own DOI<br>L: Authors must use CC-BY license	*Journal submission:* Unrestricted - manuscript can be published in any destination<br><br>*Journals directly associated with platform:* Submit directly or submit from BMC journals and more from Springer Nature portfolio listed at https://www.researchsquare.com/journals	EI: Google Scholar, Crossref, Researcher-app<br><br>MA: Openly available via Crossref<br><br>MR: Yes (HTML)

For peer review only

	literature reviews, hypotheses, opinions, theories or commentaries)		R: No reader registration required	*Facilitated submission to other journals:* Not available<br><br>PL: Yes, added by platform within one month of publication	
SciELO Preprints [40] – *Verified*	Research manuscripts only	English, Portuguese, Spanish	S: PDF or any PDF-convertible format<br>V: By PDF download, PDF viewable in browser without download<br>I: URL only<br>VP: Accepts new versions until acceptance at journal / single DOI retained for all versions<br>L: Authors must use CC-BY license<br>R: No reader registration required	*Journal submission:* Unrestricted - manuscript can be published in any destination<br><br>*Journals directly associated with platform:* None (SciELO journals when full operation launched)<br><br>*Facilitated submission to other journals:* Available for any journal using a SWORD (Simple Web-service Offering Repository Deposit) protocol-based manuscript submission platform<br><br>PL: Can be added by author or journal	EI: Google Scholar<br><br>MA: Openly available via API<br><br>MR: No
SSRN [41] – *Verified*	Multiple types (including research manuscripts, abstracts, editorials, opinion papers)	Any language – English preferred, title and abstract must be provided in English, non-English translations can be submitted and linked to English version	S: PDF<br>V: By PDF download, PDF viewable in browser without download<br>I: DOI (10.2139)<br>VP: Accepts any new versions / unknown<br>L: Unknown<br>R: No reader registration required	*Journal submission:* Unrestricted - manuscript can be published in any destination<br><br>*Journals directly associated with platform:* None<br><br>*Facilitated submission to other journals:* Not available<br><br>PL: Unknown	EI: Unknown<br><br>MA: Unknown<br><br>MR: No
Surgery Open Science – *First Look*[b] [42] – *Verified*	Content criteria as directed by associated journal (Surgery Open Science)	Unknown	S: Unknown<br>V: By PDF download, PDF viewable in browser without download<br>I: URL only<br>VP: Unknown<br>L: Unknown<br>R: Reader can view basic content (title, abstract) without account but must register to view and download full content	*Journal submission:* Manuscripts must be submitted to Surgery Open Science first, authors are then provided with the option of displaying their manuscript while under review using SSRN's 'First Look'<br><br>PL: Unknown	EI: Unknown<br><br>MA: Unknown<br><br>MR: No
Therapoid [43] – *Verified*	Research manuscripts only (multimedia content accepted in associated discussion forum)	English – Chinese also to be supported in future	S: PDF<br>V: By PDF download, PDF viewable in browser without download<br>I: URL only<br>VP: Accepts any new versions / each version receives its own DOI<br>L: Authors must use CC-BY license<br>R: No reader registration required	*Journal submission:* Unrestricted – manuscript can be published in any destination<br><br>*Journals directly associated with platform:* None<br><br>*Facilitated submission to other journals:* Not available yet (to be provided for any and only open access journals)	EI: Not indexed<br><br>MA: Openly available via Crossref<br><br>MR: No (plan to require HTML in future)

For peer review only

				PL: Yes, added by platform within one month of publication	
ViXra [44]	Multiple types (including research articles, books, theses)	Any language – English preferred, non-English translations can be provided with English version, as new versions or submitted separately	S: PDF<br>V: By PDF download, PDF viewable in browser without download<br>I: URL, Platform-specific ID<br>VP: Accepts any new versions / each version retains ID with a version number<br>L: Authors can use any license<br>R: No reader registration required	*Journal submission:* Unrestricted - manuscript can be published in any destination<br><br>*Journals directly associated with platform:* None<br><br>*Facilitated submission to other journals:* Not available<br><br>PL: Can be added by author or journal	EI: Not indexed<br><br>MA: Unknown<br><br>MR: No

[a] Where primary research is research based on empirical data and secondary research is derived from primary research, such as literature reviews and meta-reviews; [b] See SSRN for general platform information

For peer review only

Table 3: Publishing practices: screening, moderation, review, preservation & data practices

Preprint Server	Stated not peer reviewed on manuscript record webpage (M) /general server webpages (S)	Advisory Board (A) / Includes Researchers (R)	Commitment to FAIR (Findable, Accessible, Interoperable and Reusable) principles / Data availability statement (DAS)	Screening checks performed between submitting and posting (stating where this process involves active researchers)	Permanence of content (P) / Reasons for retraction (R) / Web presence of removed content (W)	Mechanisms to report concerns about posted content	Sustainability of the service (S) / Preservation of content (P)
**OSF Communities**							
AfricArxiv [1] – *Verified*	M: No<br>S: Yes	A: Yes<br>R: Yes	Yes, endorses, stated on website<br><br>DAS: Not requested or required	'moderated submission check for quality standard, good scientific practices, open science principles', including data/code availability	P: Permanent with some removal options in extraneous circumstances<br>R: Plagiarism, false or inaccurate content<br>W: Basic information remains on a tombstone page	Email administrator; helpdesk support from COS (Centre for Open Science)	S: COS receive external financial support (e.g. grant, individual); operators report no sources of income, staffed on voluntary basis<br>P: COS Preservation Fund to maintain read access for 50+ years
AgriXiv [2] – *Verified*	M: No<br>S: No	A: Yes<br>R: Yes	Yes<br><br>DAS: Unknown	No information	P: Permanent with some removal options in extraneous circumstances<br>R: Plagiarism, false or inaccurate content<br>W: Basic information remains on a tombstone page	Email administrator; helpdesk support from COS	S: COS receive external financial support (e.g. grant, individual); operators report Open Access India plans to raise funds<br>P: COS Preservation Fund to maintain read access for 50+ years
Arabixiv [3] – *Verified*	M: No<br>S: No	A: Yes<br>A: Unknown	Yes<br><br>DAS: Unknown	Arabixiv editors check for: ensuring it is academic research	P: Permanent with some removal options in extraneous circumstances<br>R: Plagiarism, false or inaccurate content<br>W: Basic information remains on a tombstone page	Email administrator; helpdesk support from COS; Readers encouraged to post concerns publicly as new article	S: COS receive external financial support (e.g. grant, individual); no further information to report from operators<br>P: COS Preservation Fund to maintain read access for 50+ years
EcoEvoRxiv [4] – *Verified*	M: No<br>S: Yes	A: Yes<br>R: Yes	Yes<br><br>DAS: Unknown	Steering committee member (*including active researchers*) checks for: competing interests, plagiarism, misconduct or integrity checks, ethical and legal compliance, funder acknowledgment	P: Permanent with some removal options in extraneous circumstances<br>R: Plagiarism, false or inaccurate content<br>W: Basic information remains on a tombstone page	Email administrator; helpdesk support from COS	S: COS receive external financial support (e.g. grant, individual); no further information to report from operators<br>P: COS Preservation Fund to maintain read access for 50+ years

For peer review only

FocUS Archive [5] – *Verified*	M: No<br>S: No	A: Yes<br>R: Yes	Yes<br><br>DAS: Unknown	There is a moderation process, no further information known	P: Permanent with some removal options in extraneous circumstances<br>R: Plagiarism, false or inaccurate content<br>W: Basic information remains on a tombstone page	Email administrator; helpdesk support from COS	S: COS receive external financial support (e.g. grant, individual); nothing to report from operators<br>P: COS Preservation Fund to maintain read access for 50+ years
Frenxiv [6] – *Verified*	M: No<br>S: No	A: Yes<br>R: Yes	Yes<br><br>DAS: Unknown	Editorial-advisory members (*including active researchers*) check for: competing interests, plagiarism, misconduct or integrity checks, authors are genuine, ethical and legal compliance, data availability	P: Permanent with some removal options in extraneous circumstances<br>R: Plagiarism, false or inaccurate content<br>W: Basic information remains on a tombstone page	Email administrator; helpdesk support from COS	S: COS receive external financial support (e.g. grant, individual); nothing to report from operators<br>P: COS Preservation Fund to maintain read access for 50+ years
INA-Rxiv [7] – *Verified*	M: No<br>S: Yes	A: Yes<br>R: Yes	Yes<br><br>DAS: Unknown	Unknown	P: Permanent with some removal options in extraneous circumstances<br>R: Plagiarism, false or inaccurate content<br>W: Basic information remains on a tombstone page	Email administrator; helpdesk support from COS	S: COS receive external financial support (e.g. grant, individual); operators endorse users to donate to COS<br>P: COS Preservation Fund to maintain read access for 50+ years
MarXiv [8] – *note: no longer on the OSF – Verified*	M: No<br>S: Yes	A: Yes<br>R: Yes	Yes<br><br>DAS: Unknown	The MarXiv team check for: plagiarism, misconduct or integrity checks, ethical and legal compliance	P: Permanent with some removal options in extraneous circumstances<br>R: Plagiarism, false or inaccurate content<br>W: Basic information remains on a tombstone page	Email administrator; helpdesk support from COS	S: COS receive external financial support (e.g. grant, individual); service moving to another host<br>P: COS Preservation Fund to maintain read access for 50+ years
MetaArXiv [9] – *Verified*	M: No<br>S: No	A: Yes<br>R: Yes	Yes<br><br>DAS: Not requested or required	BITSS staff check for: relevance of material, author name matches submitter	P: Permanent with some removal options in extraneous circumstances<br>R: Plagiarism, false or inaccurate content<br>W: Basic information remains on a tombstone page	Email administrator; helpdesk support from COS; Community moderation via commenting	S: COS receive external financial support (e.g. grant, individual); operation supported by BITSS funders and revenue<br>P: COS Preservation Fund to maintain read access for 50+ years
MindRxiv [10] – *Verified*	M: No<br>S: Yes	A: Yes<br>R: Yes	Yes<br><br>DAS: Not requested or required	Science Director at Mind & Life (*including active researchers*) checks for: relevance of material, ensuring it is academic research	P: Permanent with some removal options in extraneous circumstances<br>R: Plagiarism, false or inaccurate content<br>W: Basic information remains on a tombstone page	Email administrator; helpdesk support from COS	S: COS receive external financial support (e.g. grant, individual); nothing to report from operators<br>P: COS Preservation Fund to maintain read access for 50+ years

For peer review only

NutriXiv [11] – *Verified*	M: No<br>S: No	A: Unknown<br>R: Unknown	Yes<br><br>DAS: Unknown	No information	P: Permanent with some removal options in extraneous circumstances<br>R: Plagiarism, false or inaccurate content<br>W: Basic information remains on a tombstone page	Email administrator; helpdesk support from COS	S: COS receive external financial support (e.g. grant, individual); nothing to report from operators<br>P: COS Preservation Fund to maintain read access for 50+ years
OSF Preprints [12] – *Verified*	M: No<br>S: Yes	A: Yes<br>R: Yes	Yes<br><br>DAS: Not requested or required	OSF staff members run automated checks for spam	P: Permanent with some removal options in extraneous circumstances<br>R: Plagiarism, false or inaccurate content<br>W: Basic information remains on a tombstone page	Email administrator; helpdesk support from COS	S: COS receive external financial support (e.g. grant, individual)<br>P: COS Preservation Fund to maintain read access for 50+ years
PaleorXiv [13] – *Verified*	M: No<br>S: Yes	A: Yes<br>R: Yes	Yes<br><br>DAS: Not requested or required	A moderator from the PaleoRxiv steering committee (*including active researchers*) checks for: plagiarism, relevance of material, ensuring it is academic research	P: Permanent with some removal options in extraneous circumstances<br>R: Plagiarism, false or inaccurate content<br>W: Basic information remains on a tombstone page	Email administrator; helpdesk support from COS	S: COS receive external financial support (e.g. grant, individual); nothing to report from operators<br>P: COS Preservation Fund to maintain read access for 50+ years
PsyArXiv [14] – *Verified*	M: No<br>S: Yes	A: Yes<br>R: Yes	Yes<br><br>DAS: Not requested or required	Moderation team checks for: ethical and legal compliance, relevance of material, copyright violations, spam	P: Permanent with some removal options in extraneous circumstances<br>R: Plagiarism, false or inaccurate content<br>W: Basic information remains on a tombstone page	Email administrator; helpdesk support from COS	S: COS receive external financial support (e.g. grant, individual); nothing to report from operators<br>P: COS Preservation Fund to maintain read access for 50+ years
SocArxiv [15] – *Verified*	M: No<br>S: Yes	A: Yes<br>R: Yes	Yes<br><br>DAS: Unknown	There is a moderation process, no further information known	P: Permanent with some removal options in extraneous circumstances<br>R: Plagiarism, false or inaccurate content<br>W: Basic information remains on a tombstone page	Email administrator; helpdesk support from COS	S: COS receive external financial support (e.g. grant, individual); nothing to report from operators<br>P: COS Preservation Fund to maintain read access for 50+ years
SportRxiv [16] – *Verified*	M: No<br>S: Yes	A: Yes<br>R: Yes	Yes<br><br>DAS: Not requested or required	SportRxiv editors (*including active researchers*) check for: basic scientific standards, authors are genuine, all authors provide permission to post	P: Permanent with some removal options in extraneous circumstances<br>R: W: Basic information remains on a tombstone page	Email administrator; helpdesk support from COS	S: COS receive external financial support (e.g. grant, individual); operators report membership fees help offset costs<br>P: COS Preservation Fund to maintain read access for 50+ years
Thesis Commons [17] – *Verified*	M: No<br>S: No	A: Yes<br>R: Yes	Yes	There is no moderation process beyond that for OSF Preprints (OSF staff check for spam)	P: Permanent with some removal options in extraneous circumstances	Email administrator; helpdesk support from COS	S: COS receive external financial support (e.g. grant,

For peer review only - http://bmjopen.bmj.com/site/about/guidelines.xhtml

			DAS: Not requested or required		R: Plagiarism, false or inaccurate content<br>W: Basic information remains on a tombstone page		individual); nothing to report from operators<br>P: COS Preservation Fund to maintain read access for 50+ years
**Open Research Central infrastructure**							
AAS Open Research [18] – *Verified*	M: Yes<br>S: Yes	A: Yes<br>R: Yes	Yes, compliant, stated on website<br><br>DAS: Required	F1000's in-house editorial team check for: competing interest declaration, plagiarism, authors are genuine and at least one eligible for platform, authors provide permission to post, ethical and legal compliance, funder acknowledgment, clinical trial registration, data/code availability, minimum reporting standards	P: Permanent with some removal options in extraneous circumstances<br>R: Contravention of copyright, plagiarism, false or inaccurate content, ethical and legal issues<br>W: Basic information remains on a tombstone page	Community moderation via commenting	S: Article processing charges, covered by funding agency for AAS, Gates, HRB, MNI and Wellcome (MNI - limited to one article per lab per year)<br>P: Preprints permanently archived in Portico
AMRC Open Research [19] – *Verified*	M: Yes<br>S: Yes	A: Not yet<br>R: Still finalising advisory board					
Gates Open Research [20] – *Verified*	M: Yes<br>S: Yes	A: Yes<br>R: Yes					
HRB Open Research [21] – *Verified*	M: Yes<br>S: Yes	A: Yes<br>R: Yes					
MNI Open Research [22] – *Verified*	M: Yes<br>S: Yes	A: Not yet<br>R: Not applicable					
Wellcome Open Research [23] – *Verified*	M: Yes<br>S: Yes	A: Yes<br>R: Yes					
**Others**							
arXiv [24] – *Verified*	M: No<br>S: Yes	A: Yes<br>R: Yes	None as yet<br><br>DAS: Not requested or required	arXiv moderators (*including active researchers*) check for: plagiarism, misconduct or integrity checks, authors are genuine, ethical and legal compliance, relevance of material	P: Permanent with no removal options (as long as archive exists)<br>R: None<br>W: Not applicable	Social media or direct with authors (except copyright violations or significant issues)	S: External financial support (grants and membership program: libraries, research labs, philanthropy, government funding)<br>P: Persistent access through mirror sites, no external preservation services used as yet
Authorea [25] – *Verified*	M: No<br>S: Yes	A: No<br>R: Not applicable (no advisory board)	Yes, in process of becoming compliant, without stating on website<br><br>DAS: Not requested or required	Submissions are checked for spam	P: Content maybe removed by operator/owner only<br>R: Policy not yet in place<br>W: Not applicable (at this time)	Email administrator	S: Other business model not based on direct preprinting charges or associated journal (acquisition and investment from Atypon, part of Wiley)<br>P: Preprints permanently archived in Portico

For peer review only

bioRxiv [26] – *Verified*	M: Yes<br>S: Yes	A: Yes<br>R: Yes	None as yet<br><br>DAS: Not requested or required	bioRxiv staff and scientific affiliates (*including active researchers*) check for: plagiarism, misconduct or integrity checks, ethical and legal compliance, clinical trial registration, submission is within scope and no danger to human health	P: Permanent with withdrawal options and (rare) removal in extraneous circumstances<br>R: Removal: Legal or biohazard reasons; Withdrawal: Plagiarism, false or inaccurate content, ethical and legal issues, co-author(s) did not consent to posting<br>W: Withdrawn articles remain visible; For removed articles, basic information remains on a tombstone page	Email administrator	S: Internal (Cold Spring Harbor Laboratory) and external financial support (multiyear grant funding from Chan Zuckerberg Initiative)<br>P: Preprints permanently archived in Portico
Cell Press Sneak Peek [27] – *Verified*	M: Yes<br>S: Yes	A: Yes<br>R: Yes	Unknown	No information	P: Unknown<br>R: Unknown<br>W: Unknown	Unknown	S: Unknown<br>P: Unknown
ChemRxiv [28]	M: Yes<br>S: Yes	A: Yes<br>R: Yes	Yes, compliant, without stating on website<br><br>DAS: Not requested or required	PhD-level chemists (*including active researchers*) check for: competing interests declared, plagiarism, misconduct or integrity checks, authors are genuine, all authors provide permission to post, ethical and legal compliance, funder acknowledgment, external data/code availability	P: Permanent with some removal options in extraneous circumstances<br>R: Contravention of copyright, plagiarism, ethical and legal issues<br>W: Basic information remains on a tombstone page	Email administrator, or contact ethics departments of governing partners	S: Nothing to report from server<br>P: Agreement to dark archive content with TIB (Leibniz Information Centre for Science and Technology University Library)
ChinaXiv [29]	M: No<br>S: No	A: Yes<br>R: Yes	Unknown<br><br>DAS: Not requested or required	Project team and scientists (*including active researchers*) check for: plagiarism, misconduct or integrity checks, authors are genuine	P: Permanent with no removal options<br>R: Contravention of copyright, plagiarism, false or inaccurate content, ethical and legal issues<br>W: Basic information remains on a tombstone page	None	S: Nothing to report from server<br>P: Long term preservation in local
ESSOAr [30] – *Verified*	M: Yes<br>S: Yes	A: Yes<br>R: Yes	Yes, without stating on website<br><br>DAS: Not requested or required	Editorial board members (*including active researchers*) check for: plagiarism, misconduct or integrity checks, authors are genuine, all authors provide permission to post, submission is scholarly and within scope	P: Permanent with some removal options in extraneous circumstances<br>R: Copyright infringement or other ethical or legal issues the Advisory Board deems critical	Email administrator	S: Internal financial support (investment from Wiley and AGU)<br>P: On roadmap: preprints to be permanently archived in Portico

For peer review only

					W: Unknown		
F1000 Research [31] – *Verified*	M: Yes<br>S: Yes	A: Yes<br>R: Yes	Yes, compliant, stated on website<br><br>DAS: Required	F1000's in-house editorial team check for: competing interest declaration, plagiarism, authors are genuine and at least one eligible for platform, authors provide permission to post, ethical and legal compliance, funder acknowledgment, clinical trial registration, data/code availability, minimum reporting standards, relevance of material	P: Permanent with some removal options in extraneous circumstances<br>R: Contravention of copyright, false or inaccurate content, ethical and legal issues<br>W: Basic information remains on a tombstone page	Email administrator	S: Nothing to report from server<br>P: Preprints permanently archived in Portico
JMIR Preprints [32]	M: No<br>S: Yes	A: Unknown<br>R: Unknown	Unknown	Unknown	P: Unknown<br>R: Unknown<br>W: Unknown	Unknown	S: Unknown<br>P: Unknown
medRxiv [33] – *Verified*	M: Yes<br>S: Yes	A: In formation (January 2020)<br>R: Yes	None as yet<br><br>DAS: Required	Submission is checked for: competing interests declared, plagiarism, misconduct or integrity checks, minimal reporting standards, ethical and legal compliance, clinical trial registration, funder acknowledgment, submission is within scope and no danger to human health	P: Permanent with withdrawal options and (rare) removal in extraneous circumstances<br>R: Removal: Legal or biohazard reasons; Withdrawal: Plagiarism, false or inaccurate content, ethical and legal issues, co-author(s) did not consent to posting<br>W: Withdrawn articles remain visible; For removed articles, basic information remains on a tombstone page	Unknown	S: Supported by CSHL<br>P: Preprints permanently archived in Portico
MitoFit Preprint Archives [34]	M: Yes<br>S: Yes	A: Yes<br>R: Yes	Yes, endorses, stated on website<br><br>DAS: Encouraged	Scientific Advisory Board members (*including active researchers*) check for: competing interests declared, misconduct or integrity checks, authors are genuine, funder acknowledgment	P: Permanent with some removal options in extraneous circumstances<br>R: Plagiarism, ethical and legal issues, no appropriate declaration of conflicts of interest<br>W: Basic information remains on a tombstone page	Community moderation via commenting	S: Other business model not based on direct preprinting charges or associated journal (maintained by MitoFit DOI Data Center)<br>P: Backup kept in secondary server
NeuroImage: Clinical - *First Look* [35] – *Verified*	M: Yes<br>S: Yes	A: Unknown<br>R: Unknown	Unknown	No information	P: Unknown<br>R: Unknown<br>W: Unknown	Unknown	S: Unknown<br>P: Unknown

For peer review only

PeerJ Preprints [36] – *Verified*	M: Yes<br>S: Yes	A: Yes<br>R: Yes	None<br><br>DAS: Encouraged	PeerJ staff check for: competing interests declared, misconduct or integrity checks, ethical and legal compliance, funder acknowledgment	P: Permanent with some removal options in extraneous circumstances<br>R: False or inaccurate content, additional reasons not stated<br>W: Basic information remains on a tombstone page	Email administrator	S: Supported by associated journal publishing (PeerJ)<br>P: Preprints permanently archived in Portico and EuropePMC
Preprints with The Lancet [37] – *Verified*	M: Yes<br>S: Yes	A: Unknown<br>R: Unknown	Unknown	SSRN staff and a Lancet editor check for: competing interests declared, ethical and legal compliance, funder acknowledgment, clinical trial registration, relevance of material	P: Unknown<br>R: Unknown<br>W: Unknown	Unknown	S: Unknown<br>P: Unknown
Preprints.org [38]	M: Yes<br>S: Yes	A: Yes<br>R: Yes	None as yet<br><br>DAS: Encouraged	Preprints staff and advisory board members (*including active researchers*) check for: plagiarism (as needed), authors are genuine, ethical and legal compliance, basic scientific content, data/code availability	P: Permanent with some removal options in extraneous circumstances<br>R: Contravention of copyright, plagiarism, false or inaccurate content, ethical and legal issues, misconduct<br>W: Basic information remains on a tombstone page if DOI has been issued, otherwise no web presence retained	Email administrator	S: Supported by associated journal publishing (MDPI)<br>P: Currently no long-term preservation strategy
Research Square [39] – *Verified*	M: Yes<br>S: Yes	A: Not yet<br>R: Not applicable	None as yet<br><br>DAS: Not requested or required	In-house editorial team checks for: competing interests declared, plagiarism (only for submissions to BMC journals), ethical and legal compliance, funder acknowledgment, clinical trial registration, relevance of material, basic scientific content, language, potential for harm	P: Permanent with some removal options in extraneous circumstances<br>R: Contravention of copyright, ethical and legal issues<br>W: Basic information remains on a tombstone page	Email administrator	S: Other business model not based on direct preprinting charges or associated journal publishing (publishing services by Research Square)<br>P: Preprints permanently archived in Portico
SciELO Preprints [40] – *Verified*	M: No<br>S: No	A: Yes<br>R: Yes	Yes, endorses, without stating on website<br><br>DAS: Not requested or required	SciELO editors (*including active researchers*) will check for: competing interests declared, plagiarism, misconduct and integrity checks, ethical and legal compliance, authors are genuine, all authors provide permission to post, clinical trial registration, funder acknowledgment, data/code availability	P: Permanent with some removal options in extraneous circumstances<br>R: Contravention of copyright, plagiarism, false or inaccurate content, ethical and legal issues<br>W: No web presence retained	Email administrator	S: Internal financial support (SciELO program)<br>P: Plan to use LOCKSS-compatible preservation strategy [once full operation launched]

SSRN [41] – *Verified*	M: No<br>S: No	A: Yes<br>R: Yes	Unknown	SSRN staff check for relevance of material	P: Content maybe removed by author (or operator/owner)<br>R: Contravention of copyright, plagiarism, false or inaccurate content, ethical and legal issues<br>W: Unknown	Email administrator	S: Unknown<br>P: Unknown
Surgery Open Science - *First Look* [42] – *Verified*	M: No<br>S: No	A: Unknown<br>R: Unknown	Unknown	Unknown	P: Unknown<br>R: Unknown<br>W: Unknown	Unknown	S: Unknown<br>P: Unknown
Therapoid [43] – *Verified*	M: No<br>S: No	A: Yes<br>R: Yes	None as yet<br><br>DAS: Not requested or required	There is no pre-moderation of submissions; any checks are performed by site users (*including active researchers*) after posting	P: Permanent with some removal options in extraneous circumstances<br>R: Contravention of copyright, plagiarism, false or inaccurate content, ethical and legal issues<br>W: No web presence retained	Not yet in place: expect community moderation via forum	S: Unknown<br>P: All content is preserved – no further information reported by server
ViXra [44]	M: Yes<br>S: Yes	A: Yes<br>R: Yes	Mostly compliant, without endorsing, without stating on website<br><br>DAS: Not requested or required	Administrators do not perform formal screening checks but reject submissions in response to issues relating to: plagiarism, misconduct and integrity checks, ethical and legal compliance, format (PDF)	P: Content maybe removed by author (or operator/owner)<br>R: Contravention of copyright, plagiarism, false or inaccurate content, ethical and legal issues<br>W: No web presence retained	Email administrator	S: Sustainable low-cost web hosting service secured, previously run on donations<br>P: Long-term archiving provided by mirror site and cloud storage of database

For peer review only

Table 4: Usage metrics & features

Preprint Server	Usage metrics on abstract page						Other features			
**OSF Communities**	Number of views	Number of downloads	Number of citations (e.g. Google Scholar)	Number of comments	Altmetrics	Social media interactions (from platform not Altmetric)	Commenting (including annotation plug-ins)	Onsite search	Alerts (e.g. RSS (Really Simple Syndication) feeds, email alerts)	Other
AfricArxiv [1] – *Verified*		⊗					⊗	⊗		
AgriXiv [2] – *Verified*		⊗					⊗	⊗		
Arabixiv [3] – *Verified*		⊗					⊗	⊗		
EcoEvoRxiv [4] – *Verified*		⊗						⊗		
FocUS Archive [5] – *Verified*		⊗						⊗		
Frenxiv [6]] – *Verified*		⊗					⊗	⊗		
INA-Rxiv [7]] – *Verified*		⊗					⊗	⊗		
MarXiv [8] *– note: no longer on the OSF – Verified*		⊗					⊗	⊗		
MetaArXiv [9] – *Verified*		⊗					⊗	⊗		
MindRxiv [10] – *Verified*		⊗					⊗	⊗		
NutriXiv [11] – *Verified*		⊗					⊗	⊗		
OSF Preprints [12] – *Verified*		⊗					⊗	⊗		
PaleorXiv [13] – *Verified*		⊗					⊗	⊗		
PsyArXiv [14] – *Verified*		⊗					⊗	⊗		
SocArxiv [15] – *Verified*		⊗					⊗	⊗	⊗	
SportRxiv [16] – *Verified*		⊗					⊗	⊗		
Thesis Commons [17] – *Verified*		⊗					⊗	⊗		
**Open Research Central infrastructure**										
AAS Open Research [18] – *Verified*	⊗	⊗	⊗		⊗		⊗	⊗		'Link to Google Scholar citations, Blog and gateways'

For peer review only

AMRC Open Research [19] – *Verified*	⊗	⊗	⊗		⊗		⊗	⊗		'Link to Google Scholar citations'
Gates Open Research [20] – *Verified*	⊗	⊗	⊗		⊗		⊗	⊗		'Link to Google Scholar citations, Gateways'
HRB Open Research [21] – *Verified*	⊗	⊗	⊗		⊗		⊗	⊗		'Link to Google Scholar citations, Blog'
MNI Open Research [22] – *Verified*	⊗	⊗	⊗		⊗		⊗	⊗		'Link to Google Scholar citations'
Wellcome Open Research [23] – *Verified*	⊗	⊗	⊗		⊗		⊗	⊗		'Link to Google Scholar citations, Blog and gateways'
**Others**										
arXiv [24] – *Verified*								⊗	⊗	
Authorea [25] – *Verified*	⊗	⊗				⊗	⊗			'Forking (copying an article that may or may not be authored by you), follow the article'
bioRxiv [26] – *Verified*	⊗	⊗		⊗	⊗	⊗	⊗	⊗	⊗	'Channels, PREreview reviews linked, PreLights blog linked, Pingbacks to blog, PCI, biOverlay and Twitter thread embedded'
Cell Press Sneak Peek [27] – *Verified*	⊗	⊗								Unknown 'other'
ChemRxiv [28]	⊗	⊗	⊗		⊗					Unknown 'other'
ChinaXiv [29]	⊗	⊗					⊗		⊗	'News, Author blacklist'
ESSOAr [30] – *Verified*		⊗			⊗			⊗	⊗	'Plaudit, QR code, bookmarks, reference management tools'
F1000 Research [31] – *Verified*	⊗	⊗	⊗		⊗		⊗	⊗	⊗	'Link to Google Scholar citations'
JMIR Preprints [32]						⊗	⊗	⊗		
medRxiv [33] – *Verified*	⊗	⊗		⊗	⊗	⊗	⊗	⊗	⊗	
MitoFit Preprint Archives [34]										None
NeuroImage: Clinical - *First Look* [35] – *Verified*	⊗	⊗								Unknown 'other'

For peer review only

PeerJ Preprints [36] – *Verified*	⊗	⊗		⊗		⊗	⊗	⊗	⊗	
Preprints with The Lancet [37] – *Verified*	⊗	⊗								Unknown 'other'
Preprints.org [38]	⊗	⊗		⊗	⊗		⊗	⊗	⊗	Embedded bookmark tabs for reading and reference management tools
Research Square [39] – *Verified*	⊗	⊗		⊗	⊗		⊗	⊗		Email alerts with comments
SciELO Preprints [40] – *Verified*	⊗	⊗			⊗	⊗ (when regular operation launches in 2020)	⊗ (when regular operation launches in 2020)	⊗ (when regular operation launches in 2020)		
SSRN [41] – *Verified*	⊗	⊗								Plum metrics
Surgery Open Science - *First Look* [42] – *Verified*	⊗	⊗						⊗	⊗ (SSRN subscription)	
Therapoid [43] – *Verified*	'metrics coming soon'						⊗	⊗	⊗	'Blockchain technology being implemented'
ViXra [44]	⊗						⊗		⊗	

For peer review only

Table 5: Metadata contents

Preprint Server	Title	DOI or identifier	Publication or deposition date	Author name(s)	Author affiliation(s)	Funder acknowledge-ment(s)	Subject category	License	Abstract	Full-text content	References	Relational link to journal publication version (where it exists)	Other
**OSF Communities**													
AfricArxiv [1] – *Verified*	⊗	⊗	⊗	⊗	⊗		⊗	⊗	⊗			⊗	
AgriXiv [2] – *Verified*	⊗	⊗	⊗	⊗			⊗	⊗	⊗			⊗	
Arabixiv [3] – *Verified*	⊗	⊗	⊗	⊗			⊗	⊗	⊗			⊗	
EcoEvoRxiv [4] – *Verified*	⊗	⊗	⊗	⊗			⊗	⊗	⊗				
FocUS Archive [5] – *Verified*	⊗	⊗	⊗	⊗			⊗	⊗	⊗			⊗	
Frenxiv [6] – *Verified*	⊗	⊗	⊗	⊗			⊗	⊗	⊗			⊗	
INA-Rxiv [7] – *Verified*	⊗	⊗	⊗	⊗			⊗	⊗	⊗			⊗	
MarXiv [8] – *Verified*	⊗	⊗	⊗	⊗			⊗	⊗	⊗			⊗	
MetaArXiv [9]– *Verified*	⊗	⊗	⊗	⊗			⊗	⊗	⊗			⊗	
MindRxiv [10] – *Verified*	⊗	⊗	⊗	⊗			⊗	⊗	⊗			⊗	
NutriXiv [11] – *Verified*	⊗	⊗	⊗	⊗					⊗				
OSF Preprints [12] – *Verified*	⊗	⊗	⊗	⊗			⊗	⊗	⊗			⊗	
PaleorXiv [13] – *Verified*	⊗	⊗	⊗	⊗			⊗	⊗	⊗			⊗	
PsyArXiv [14] – *Verified*	⊗	⊗	⊗	⊗			⊗	⊗	⊗			⊗	
SocArxiv [15] – *Verified*	⊗	⊗	⊗	⊗			⊗	⊗	⊗			⊗	
SportRxiv [16] – *Verified*	⊗	⊗	⊗	⊗			⊗	⊗	⊗			⊗	
Thesis Commons [17] – *Verified*	⊗	⊗	⊗	⊗			⊗	⊗	⊗			⊗	
**Open Research Central infrastructure**													

For peer review only

For peer review only - http://bmjopen.bmj.com/site/about/guidelines.xhtml

AAS Open Research [18] – *Verified*	⊗	⊗	⊗	⊗	⊗	⊗	⊗		⊗	⊗			Competing interests, Referee (name, affiliation, report), Commenter (name, affiliation, comment text), Study type, Article type
AMRC Open Research [19] – *Verified*													
Gates Open Research [20] – *Verified*													
HRB Open Research [21] – *Verified*													
MNI Open Research [22] – *Verified*													
Wellcome Open Research [23] – *Verified*													
**Others**													
arXiv [24] – *Verified*	⊗	⊗	⊗	⊗					⊗			⊗	
Authorea [25] – *Verified*	⊗	⊗	⊗	⊗	⊗		⊗		⊗	⊗			Keywords
bioRxiv [26] – *Verified*	⊗	⊗	⊗	⊗	⊗		⊗	⊗	⊗	⊗	⊗	⊗	
Cell Press Sneak Peek[b] [27] – *Verified*													No information
ChemRxiv [28]	⊗	⊗	⊗	⊗	⊗	⊗	⊗	⊗	⊗	⊗	⊗		
ChinaXiv [29]	⊗	⊗	⊗	⊗			⊗		⊗				
ESSOAr [30] – *Verified*	⊗	⊗	⊗	⊗	⊗	⊗	⊗	⊗	⊗			⊗	ORCID, keywords
F1000 Research [31] – *Verified*	⊗	⊗	⊗	⊗	⊗	⊗	⊗		⊗	⊗			Competing interests, Article is a Faculty Review (yes/no), Referee (name, affiliation, report), Commenter (name, affiliation,

For peer review only

													comment text), Study type, Article type
JMIR Preprints [32]	⊗	⊗	⊗	⊗				⊗	⊗				Journal currently submitted to
medRxiv [33] – *Verified*	⊗	⊗	⊗	⊗	⊗	⊗	⊗	⊗	⊗		⊗	⊗	
MitoFit Preprint Archives [34]	⊗	⊗	⊗	⊗	⊗	⊗	⊗	⊗	⊗				ORCID if provided, Language, Version
NeuroImage: Clinical - *First Look*[b] [35] – *Verified*													No information
PeerJ Preprints [36] – *Verified*	⊗	⊗	⊗	⊗	⊗	⊗	⊗	⊗	⊗			⊗	
Preprints with The Lancet[b] [37] – *Verified*													No information
Preprints.org [38]	⊗	⊗	⊗	⊗				⊗	⊗			⊗	
Research Square [39] – *Verified*	⊗	⊗	⊗	⊗	⊗	⊗	⊗	⊗	⊗	⊗	⊗	⊗	
SciELO Preprints [40] – *Verified*	⊗		⊗	⊗	⊗		⊗	⊗	⊗				Language
SSRN [41] – *Verified*	⊗	⊗	⊗	⊗					⊗		⊗		
Surgery Open Science – *First Look*[b] [42] – *Verified*													No information
Therapoid [43] – *Verified*	⊗	⊗	⊗	⊗			⊗		⊗				"Anything else the author provides"
ViXra [44]	⊗	⊗	⊗	⊗					⊗				

For peer review only

Preprint platform websites

1. AfricaArxiv https://info.africarxiv.org/
2. AgriXiv https://agrixiv.org
3. Arabixiv https://arabixiv.org/
4. EcoEvoRxiv https://ecoevorxiv.org
5. FocUS Archive https://osf.io/preprints/focusarchive/
6. Frenxiv https://frenxiv.org
7. INA-Rxiv https://osf.io/preprints/inarxiv
8. MarXiv https://marxiv.org
9. MetaArXiv https://osf.io/preprints/metaarxiv/
10. MindRxiv https://mindrxiv.org
11. NutriXiv https://osf.io/preprints/nutrixiv
12. OSF Preprints https://osf.io/preprints/
13. PaleorXiv https://paleorxiv.org
14. PsyArXiv https://psyarxiv.com
15. SocArXiv https://osf.io/preprints/socarxiv
16. SportRxiv https://osf.io/preprints/sportrxiv
17. Thesis Commons https://thesiscommons.org
18. AAS Open Research https://aasopenresearch.org/
19. AMRC Open Research https://amrcopenresearch.org/
20. Gates Open Research https://gatesopenresearch.org/
21. HRB Open Research https://hrbopenresearch.org/
22. MNI Open Research https://mniopenresearch.org/
23. Wellcome Open Research https://wellcomeopenresearch.org/
24. arXiv https://arxiv.org
25. Authorea https://www.authorea.com
26. bioRxiv https://www.biorxiv.org/
27. Cell Press Sneak Peek https://papers.ssrn.com/sol3/Jeljour_results.cfm?form_name=journalBrowse&journal_id=3184889
28. ChemRxiv https://chemrxiv.org
29. ChinaXiv http://chinaxiv.org
30. ESSOAr https://www.essoar.org
31. F1000 Research https://www.essoar.org
32. JMIR Preprints https://preprints.jmir.org/
33. medRxiv https://www.medrxiv.org
34. MitoFit Preprint Archives https://www.mitofit.org/index.php/MitoFit_Preprint_Archives
35. NeuroImage: Clinical – *First Look*
https://papers.ssrn.com/sol3/JELJOUR_Results.cfm?form_name=journalBrowse&journal_id=3178959
36. PeerJ Preprints https://peerj.com/preprints/
37. Preprints with The Lancet https://papers.ssrn.com/sol3/JELJOUR_Results.cfm?form_name=journalBrowse&journal_id=3184962
38. Preprints.org https://www.preprints.org/
39. Research Square https://www.researchsquare.com
40. SciELO Preprints https://preprints.scielo.org/index.php/scielo
41. SSRN https://www.ssrn.com
42. Surgery Open Science – *First Look*
https://papers.ssrn.com/sol3/Jeljour_results.cfm?form_name=journalBrowse&journal_id=3303309
43. Therapoid https://therapoid.net
44. ViXra http://vixra.org

For peer review only

Supplementary Table 1: Reasons for excluded preprint platforms

Name of Site	Reason for Exclusion
Chemweb	Inactive
Centre for Health Economics and Policy Analysis (CHEPA)	Inactive
ClinMed NetPrints	Inactive
Cogprints	Inactive
CSTC	Inactive
K-Theory Preprint Archives	Inactive
Mathematics Preprint Search System (MPRESS)	Inactive
National Advisory Committee for Aeronautics (NACA)	Inactive
Nature Precedings	Inactive
The Winnower	Inactive
Geometry Center's Preprints	Inactive
World Health Organisation Zika Open repository	Inactive
Instant Math Preprints (Yale Mathematics Preprint Bulletin Board)	Inactive
Organisation Européenne pour la Recherche Nucléaire document platform	Repository
Hyper Articles en Ligne (HAL)	Repository
ResearchGate	Repository
White Rose Consortium e-Prints Repository	Repository
Zenodo	Repository
Organisation Européenne pour la Recherche Nucléaire Print	Offline
Fermilab	Offline
Physics Information Exchange (PIE)	Offline
Radio Astronomy Preprints – RAPsheet	Offline
Space Telescope Preprints – STEPsheet	Offline
Advance: a SAGE preprints community	Scope: Humanities and Social Sciences
BodoArXiv	Scope: Medieval Studies
Cryptology ePrint Archive	Scope: Cryptology
CORE repository	Scope: Humanities
EarthArXiv	Scope: Earth Sciences
EconStor	Scope: Economics and Business Studies
ECSarXiv	Scope: Electrochemistry and solid state science
engrXiv	Scope: Engineering
E-LIS	Scope: Library and Information Science
Electronic Colloquium on Computational Complexity	Scope: Computer Science
Institute for Fiscal Studies (IFS) Working Papers	Scope: Economics
LawArxiv	Scope: Law
LIS Scholarship Archive	Scope: Library and Information Science
LingBuzz	Scope: Linguistics
MediArXiv	Scope: Media, Film and Communication Studies
Mathematical Physics Preprint Archive (mp_arc)	Scope: Mathematical Physics and Related Areas
National Bureau of Economic Research (NBER) Working Papers	Scope: Economics
Networked Computer Science Technical Reference Library (NCSTRL)	Scope: Computer Science
Philsci Archive	Scope: Philosophy of Science
Munich Personal RePEc Archive (MPRA)	Scope: Economics
Social Science Open Access Repository (SSOAR)	Scope: Social Sciences
Stanford Physics Information Retrieval System (SPIRES)	Scope: Physics
WorldBank's Policy Research Working Paper Series (PRWPs)	Scope: Economics

For peer review only

BMJ Open

A systematic examination of preprint platforms for use in the medical and biomedical sciences setting

Journal:	*BMJ Open*
Manuscript ID	bmjopen-2020-041849.R1
Article Type:	Original research
Date Submitted by the Author:	27-Aug-2020
Complete List of Authors:	Kirkham, Jamie J.; Manchester University, Biostatistics<br>Penfold, Naomi; ASAPbio<br>Murphy, Fiona; Murphy Mitchell Consulting Ltd<br>Boutron, Isabelle; Center of Research in Epidemilogy and Statistics Sorbone Paris, CRESS UMR 1153, METHODS team,<br>Ioannidis, John; Stanford University, Stanford Prevention Research Center, Department of Medicine and Department of Health Research and Policy<br>Polka, Jessica; ASAPbio<br>Moher, David; Ottawa Hospital Research Institute, Ottawa Methods Centre
**Primary Subject Heading**:	Medical publishing and peer review
Secondary Subject Heading:	Communication, Research methods
Keywords:	STATISTICS & RESEARCH METHODS, MEDICAL JOURNALISM, MEDICAL ETHICS

SCHOLARONE™
Manuscripts

I, the Submitting Author has the right to grant and does grant on behalf of all authors of the Work (as defined in the below author licence), an exclusive licence and/or a non-exclusive licence for contributions from authors who are: i) UK Crown employees; ii) where BMJ has agreed a CC-BY licence shall apply, and/or iii) in accordance with the terms applicable for US Federal Government officers or employees acting as part of their official duties; on a worldwide, perpetual, irrevocable, royalty-free basis to BMJ Publishing Group Ltd ("BMJ") its licensees and where the relevant Journal is co-owned by BMJ to the co-owners of the Journal, to publish the Work in this journal and any other BMJ products and to exploit all rights, as set out in our licence.

The Submitting Author accepts and understands that any supply made under these terms is made by BMJ to the Submitting Author unless you are acting as an employee on behalf of your employer or a postgraduate student of an affiliated institution which is paying any applicable article publishing charge ("APC") for Open Access articles. Where the Submitting Author wishes to make the Work available on an Open Access basis (and intends to pay the relevant APC), the terms of reuse of such Open Access shall be governed by a Creative Commons licence – details of these licences and which Creative Commons licence will apply to this Work are set out in our licence referred to above.

Other than as permitted in any relevant BMJ Author's Self Archiving Policies, I confirm this Work has not been accepted for publication elsewhere, is not being considered for publication elsewhere and does not duplicate material already published. I confirm all authors consent to publication of this Work and authorise the granting of this licence.

For peer review only

A systematic examination of preprint platforms for use in the medical and biomedical sciences setting

Jamie J Kirkham[1*], Naomi Penfold[2], Fiona Murphy[3], Isabelle Boutron[4], John PA Ioannidis[5], Jessica K Polka[2], David Moher[6,7]

[1]Centre for Biostatistics, Manchester Academic Health Science Centre, University of Manchester, Manchester, United Kingdom.

[2]ASAPbio, San Francisco, CA, USA.

[3]Murphy Mitchell Consulting Ltd.

[4]Université de Paris, Centre of Research in Epidemiology and Statistics (CRESS), Inserm, Paris, F-75004 France.

[5]Meta-Research Innovation Center at Stanford (METRICS) and Departments of Medicine, of Epidemiology and Population Health, of Biomedical Data Science, and of Statistics, Stanford University, Stanford, CA, USA.

[6]Centre for Journalology, Clinical Epidemiology Program, Ottawa Hospital Research Institute, Ottawa, Canada.

[7]School of Epidemiology and Public Health, Faculty of Medicine, University of Ottawa, Ottawa, Canada.

[*]Corresponding Author:

Professor Jamie Kirkham

Centre for Biostatistics
Faculty of Biology, Medicine and Health
The University of Manchester
Jean McFarlane Building
Oxford Road
Manchester, M13 9PL, UK

Email: jamie.kirkham@manchester.ac.uk
Tel: +44 (0)161 275 1135

For peer review only

ABSTRACT

Objectives

The objective of this review is to identify all preprint platforms with biomedical and medical scope and to compare and contrast the key characteristics and policies of these platforms.

Study Design and Setting

Preprint platforms that were launched up to 25th June 2019 and have a biomedical and medical scope according to MEDLINE's journal selection criteria were identified using existing lists, web-based searches and the expertise of both academic and non-academic publication scientists. A data extraction form was developed, pilot-tested and used to collect data from each preprint platform's webpage(s).

Results

A total of 44 preprint platforms were identified as having biomedical and medical scope, 17 (39%) were hosted by the Open Science Framework preprint infrastructure, six (14%) were provided by F1000 Research Ltd (the Open Research Central infrastructure) and 21 (48%) were other independent preprint platforms. Preprint platforms were either owned by non-profit academic groups, scientific societies or funding organisations (n=28; 64%), owned/partly owned by for-profit publishers or companies (n=14; 32%) or owned by individuals/small communities (n=2; 5%). Twenty-four (55%) preprint platforms accepted content from all scientific fields although some of these had restrictions relating to funding source, geographical region or an affiliated journal's remit. Thirty-three (75%) preprint platforms provided details about article screening (basic checks) and 14 (32%) of these actively involved researchers with context expertise in the screening process. Almost all preprint platforms allow submission to any peer-reviewed journal following publication, have a preservation plan for read-access, and most have a policy regarding reasons for retraction and the sustainability of the service.

Conclusion

A large number of preprint platforms exist for use in biomedical and medical sciences, all of which offer researchers an opportunity to rapidly disseminate their research findings onto an open-access public server, subject to scope and eligibility.

Keywords: Biomedicine, Medicine, Preprint, Preprint platform, Preprint server, Review, Scholarly Publishing

For peer review only

Strengths and limitations of this study

- We developed robust methodology for systematically identifying relevant preprint platforms and involved platform owners/representatives wherever possible to verify data.
- We undertook an internal pilot of developing and testing out the data collection form in collaboration with a preprint platform owner and funders.
- For platforms that had a partner journal and without verification, it was sometimes unclear if the policy information related to the journal, preprint platform or both.
- We provide a searchable database as a valuable resource for researchers, funders and policymakers in the biomedical and medical science field to determine which preprint platforms are relevant to their research scope and which have the functionality and policies that they value most.
- We plan to update this searchable database periodically to include any new relevant preprint platforms and to amend any changes in policy.

For peer review only

Introduction

A preprint is an non-peer reviewed scientific manuscript that authors can upload to a public preprint platform and make available almost immediately without formal external peer review. Posting a preprint enables researchers to 'claim' priority of discovery of a research finding; this can be particularly useful for early-career researchers in a highly competitive research environment. Some preprint platforms provide digital object identifier (DOIs) for each included manuscript. This information can be included in grant applications. Indeed, progressive granting agencies are recommending applicants include preprints in their applications (e.g., National Institutes of Health (NIH, USA) [1] and in the UK, preprints are becoming recognised as eligible outputs in the Research Excellence Framework (REF) exercise which assesses institutional research performance [2].

Preprints have been widely used in the physical sciences since the early 1990s, and with the creation of the repository of electronic articles, arXiv, over 1.6 million preprints or accepted/published manuscripts have been deposited on this platform alone [3]. Since September 2003, arXiv has supported the sharing of quantitative biology preprints under the q-bio category. The use of preprints in biomedical sciences is increasing, leading to the formation of the scientist-driven initiative ASAPbio (Accelerating Science and Publication in biology) to promote their use [4]. A preprint platform dedicated to life-science-related research (bioRxiv) founded in 2013 has already attracted nearly 80,000 preprints [5]. This platform was set up to capture science manuscripts from all areas of biology, however, medRxiv was launched in June 2019 to provide a dedicated platform and processes for preprints in medicine and health related sciences [6] and it already hosts over 3400 preprints, becoming particularly popular with COVID-19. The Center for Open Science [7] has also developed web infrastructure for these new 'Rxiv' (pronounced "archive") services [8], while F1000 Research Ltd has provided instances of its post-publication peer review and publishing platform for use by several funders (e.g. Wellcome Trust) and research institutions to encourage preprint-first research publishing [9]. Recently, several large publishers (Springer Nature, Wiley, Elsevier) have developed, co-developed or acquired preprint platforms or services, and in April 2020, SciELO launched a preprint platform that works with Open Journal Systems [10]. Many other preprint platforms also support dissemination of biomedical and medical sciences within their broader multi-disciplinary platforms.

For peer review only

Given the increase in the use and profile of preprint platforms, it is increasingly important to identify how many such platforms exist and to understand how they operate in relation to policies and practices important for dissemination. With this aim in mind, we conduct a review to identify all preprint platforms that have biomedical and medical science scope and contrast them in terms of their unique characteristics (e.g. scope of the preprint, preprint ownership) and policies (e.g. administrative checking, copyright and licensing). We also provide a searchable repository of the platforms identified so that researchers, funders and policymakers have access to a structured approach for identifying preprint platforms that are relevant to their research area.

Terminology

We define a preprint according to the COPE (Committee of Publication Ethics) definition:

"*A preprint is a scholarly manuscript posted by the author(s) in an openly accessible platform, usually before or in parallel with the peer review process*." [11].

Any platform or server that hosts a collection of preprints will be referred to as a *preprint platform*. We use 'platform' instead of 'server' because, within this definition, we include both servers with no dedicated formal peer-review service and platforms where a manuscript has been submitted for peer review and is openly available to view *before* the peer review is formally complete.

Methods

Preprint platform identification

A preliminary list of potentially relevant preprint platforms was identified using Martyn Rittman's original list [12] and extended using a basic *Google* web-search using the search term '*preprint*' and the knowledge of the Steering Group (study authors). Additional preprint platforms that were launched up to 25th June 2019 were included.

Preprint platform selection

We included any preprint platform that has biomedical or medical scope according to MEDLINE's journal selection criteria [13]. Generally this covers: "[manuscripts] *predominantly devoted to reporting original investigations in the biomedical and health sciences, including research in the basic sciences; clinical trials of therapeutic agents; effectiveness of diagnostic or*

For peer review only

therapeutic techniques; or studies relating to the behavioural, epidemiological, or educational aspects of medicine."

We aimed to be over-inclusive such that preprint platforms that hosted work within the above MEDLINE definition of scope amongst a broader scope (such as "all physical, chemical and life sciences") were included. For inclusion, the platforms primary focus needed to act as a preprint platform rather than a more general repository where preprints might be incidentally deposited. Platforms were included without any language restrictions on the content accepted for posting. Eligibility of preprint platforms was arrived at in discussion with two authors (JJK and NP) and independently approved by the Steering Group. Preprint platforms were excluded for any of the following reasons: they were no longer active (as of June 25, 2019); they were print only or had no web-presence ('offline'); their primary function was classed as a general purpose repository with no exclusive preprint functionality. We also excluded service platforms that only host postprints (after peer review), such as Science magazine's 'first release'.

Data extraction items

A data collection form was developed by the Steering Group which aimed to capture both preprint platform characteristics and policies. The form was pilot-tested with bioRxiv and revised accordingly following discussion with the platform owner. The final agreed data collection form is available online [14]. In brief, we extracted information on the preprints scope, ownership, governance, indexing and citation identifiers, submission formatting, visibility/versioning, article processing charges, publication timings, editorial board membership and for-profit or not-for-profit status. We also collected data on any checking/screening before preprint posting, open access/copyright and licensing options, sustainability and long-term preservation strategies, usage metrics and the simultaneous deposition policy relating to a manuscript submitted to a journal and the manuscript on the platform, and, if appropriate, policies about the deposition of accepted and published papers onto the platform.

Data extraction process

Manual extraction was completed for each platform using information found on the platform's website where content was directly accessible or found on associated webpages provided by the platform (for example, the 'About' pages for many OSF platforms linked to external websites provided by the platform operators). Verbatim text from the online search was recorded alongside any relevant web links. The completed data extraction form was then sent to the platform contacts (usually the platform owner), who were asked to check the data for completeness, fill in any

For peer review only

missing fields and respond to any queries. Where an independent review could not be undertaken due to language barriers on the platforms website, the platform owner/representative provided the data. On receiving the responses from the platforms, the researcher updated the data form, in some cases simplifying the text records into categorised information. These data were then returned to the platform to confirm the data were accurate and as complete as possible, and these records were then recorded as 'verified by the platform representative/owner'.

If no contact with the platform was established, a second researcher independently completed the data extraction using information found on the platform's website and consensus was reached. The completed data form was sent to the platforms informing them that the included information would be presented about their platform as 'unverified' data. The deadline for preprint platforms to approve any information and to confirm that all data could be shared publicly was 19th January 2020. Further datasets and records were updated with information provided up to 27th January 2020, and are available on the Zenodo repository [14].

Reporting of results

The preprint platform characteristics and policies were summarised descriptively and divided into preprint platforms (i) hosted on the Open Science Framework (OSF) Preprints infrastructure, (ii) provided by the Open Research Central infrastructure, and (iii) all other eligible platforms. Characteristics are presented as: (a) the scope and ownership of each platform; (b) content-specific characteristics and information relating to submission, journal transfer options, and external discoverability; (c) screening, moderation, and permanence of content; (d) usage metrics and other features; and (e) metadata.

Ethical approval: Not required. This is a descriptive study of publicly available information made available on websites. Data was confirmed by preprint platform owners/representatives using only email contacts available on those public websites.

Patient and public involvement: No patients were involved in setting the research question nor were they involved in the design, implementation and reporting of the study. There are no plans to involve patients in the dissemination of results.

Results

From all sources, ninety potentially eligible preprint platforms were identified for this review, although 46 were excluded based on scope (n=23), inactivity (n=13), no online presence (n=5) or

For peer review only

were general repositories (n=5) (Figure 1). A list of excluded preprint platforms can be found in Supplementary Table 1. Of the 44 included preprint platforms, 17 were hosted by the OSF preprint infrastructure (although MarXiv is no longer part of the OSF family), six were provided by the Open Research Central infrastructure and 21 were other independent preprint platforms (Figure 1). Of the 21 independent preprints platforms, four were First Look platforms (Cell Press Sneak Peek, Preprints with the Lancet, NeuroImage: Clinical, and Surgery Open Science). Whilst meeting the criteria for inclusion in this review, PeerJ Preprints decided to accept no new preprints after 30th September 2019. Thirty-eight (86%) of the 44 preprint platforms verified their own data. We present the data tables in this manuscript, though all tables and raw data are available in the Zenodo repository [14]. A searchable database of all the preprint platform information is also available (https://asapbio.org/preprint-servers).

Scope and ownership of preprint platforms

Twenty-eight platforms (64%) are owned by non-profit academic groups, scientific societies or funding organisations while two platforms are owned by individuals or small communities (Frenxiv and ViXra) (Table 1). Fourteen preprint platforms (32%) are affiliated or partly owned by for-profit publishers or companies; however, the preprint service part of their operation was declared as non-profit for three of these (Preprints.org, ESSOAr and MitoFit Preprint Archives). Of the preprint platforms associated with 'for-profit' status, only F1000 Research requires authors to pay an article processing charge.

Twenty-four (55%) preprint platforms accepted articles that covered multidisciplinary scope while 20 (45%) were discipline specific (e.g. PsyArXiv for psychological research) (Table 1). Despite the multidisciplinary scope, there were some further restrictions for some of the platforms, for example, there are five regional platforms (AfricArxiv, Arabixiv, Frenxiv, INA-Rxiv, ChinaXiv) aimed mostly at research being conducted in a specific geographical region, however, the content of these articles are globally accessible. The Open Research Central platforms also only accept articles that are funded by certain funders (e.g. Wellcome Open Research platform only accepts research funded by the Wellcome Trust). Some preprint platforms also only allow articles that fit the remit of their affiliated journals (e.g. Cell Press Sneak Peek). Across all platforms, the median time that they have been active is 32 months (range 10 months, medRxiv to 28 years 8 months, arXiv). In that time, over 2.72 million preprints have been posted and in 2020, two platforms (Research Square and bioRxiv) have averaged more than 2,500 biomedical postings per month.

For peer review only

Submission, journal transfer options and external discoverability

Where the information is known, all preprint platforms support the English language, and all accept research articles (with the exception of Thesis Commons which accepts only theses) (Table 2). Some platforms also accept other languages and other article types including research presentation slides and posters. Readers can access the full content of articles from all platforms with the exception of JMIR Preprints and some of the First Look platforms (Cell Press Sneak Peek, Preprints with the Lancet and Surgery Open Science) where reader registration is required. All platforms support PDF as the main viewing option, for some platforms this can be viewed in the browser while for others it requires a download. For all platforms, authors can submit articles using either a Word doc or as a PDF, with many platforms offering authors a choice of licensing, although where authors do not get a choice, the license required is commonly the CC-BY license.

In general, the Open Science Framework (OSF) and many of the other platforms allow authors to submit their articles to any journal although in some cases there is facilitated submission to certain journals, for example, for bioRxiv there is a host of direct transfer journal options (Table 2). Authors submitting to F1000 Research, the Open Research platforms and all First Look platforms can only submit articles to journals associated with the platform. Where the information is available, all platforms with the exception of Therapoid and ViXra are externally indexed and most are indexed on *Google Scholar*.

Screening, moderation, and permanence of content

Thirty-three (75%) preprint platforms provided some detail about article screening, while two (FocUS Archive and SocArxiv) do mention checks although the details of such checks are unknown (Table 3). Therapoid does not perform any screening checks but relies on a moderation process by site users following article posting and ViXra does not perform screening checks but will retract articles in response to issues. Fourteen (32%) preprint platforms that perform screening checks actively involved researchers with content expertise in this process. The three most common screening checks performed related to scope of the article (e.g. scientific content, not spam, relevant material, language), plagiarism and legal/ethical/societal issues and compliance. Only three preprint platforms (Research Square, bioRxiv and medRxiv) check whether the content contains unfounded medical claims.

For peer review only

All F1000 platforms (inclusive of Open Research ones), MitoFit Preprint Archives, PeerJ Preprints and Preprints.org describe policies online in relation to NIH guidance for reporting preprints [15] with regards to plagiarism, competing interests, misconduct and all other hallmarks of reputable scholarly publishing (Table 3). Some preprint platforms do have policies but fall short of transparently making these policies visible online while some platforms have no policies. If content is withdrawn, some platforms ensure that the article retains a web presence (e.g. basic information on a tombstone page) although this was not standard across all platforms. Almost all platforms have a preservation plan (or are about to implement) for read access. Most commonly, platforms have set up an archiving agreement with Portico. Others have made their own arrangements: as a notable example, the OSF platforms are associated with a preservation fund provided by the Center for Open Science (COS) to maintain read access for over 50 years. In addition, most platforms have details on the sustainability of the service, for the OSF platforms this come from an external source (e.g. grants to support the COS framework), while for the Open Research Central infrastructure platforms this comes from article processing charges covered by the respective funding agencies. For some of the other platforms, funding is received from either internal or external sources or from other business model services (e.g. from associated journal publishing).

Usage metrics and other features

With the exception of arXiv and MitoFit Preprint Archives (Therapoid metrics arriving soon), all preprint platforms have some form of usage metrics, and apart from JMIR Preprints and ViXra all provide the number of article downloads on the abstract page (Table 4). The OSF preprints are limited to downloads but the Open Research Central platforms also include the number of views, number of citations and altmetrics, whilst some of the independent platforms also include details of social media interactions direct from the platform (as opposed to the altmetric attention score). Most platforms (n=33; 75%) have some form of commenting and onsite search options (35; 80%), and some (mostly but not exclusively to the independent platforms) have alerts such as RSS feeds or email alerts.

Metadata

Forty (91%) of platforms provided information on metadata and all provide the manuscript title, publication date, abstract, and author names in the metadata (Table 5). Nearly all of these with the exception of SciELO Preprints provide a DOI or other manuscript identifier as well. The

For peer review only - http://bmjopen.bmj.com/site/about/guidelines.xhtml

majority also offer subject categories (n=34) and license information (n=26) but less than half include author affiliations (n=17) and funder acknowledgements (n=13). Eleven platforms (all six platforms under the Open Research Central infrastructure, Authorea, bioRxiv, ChemRxiv, F1000 Research, Research Square) offer full-text content, but only five include references in the metadata. Half of the platforms (n=22) offer a relational link to the journal publication (if it exists) in the metadata.

Discussion

Forty-four preprint platforms were identified that considered biomedical and medical scope. This review characterises each of these preprint platforms such that authors can make a more informed choice about which ones might be relevant to their research field. Moreover, funders can use the data from this review to compare platforms if they wish to explicitly support and/or encourage their researchers to use certain platforms.

Preprint platforms are fast evolving and despite our cutoff of 25th June 2019, we are aware of new eligible preprint platforms that have been or are about to be launched after this date, for example Open Anthropology Research Repository (OARR) [16] and Cambridge Open Engage [17]. However, the recent advancements in the number of preprint platforms in this field has meant that one platform in this review (PeerJ Preprints) ceased to accept new preprints from the end of September 2019 to focus on their peer-reviewed journal activities [18]. Through our searchable database (https://asapbio.org/preprint-servers), we will endeavour to keep this information up-to-date. More specifically the database will be maintained by ASAPbio for at least the next two years, and longer pending additional funding, but will be available as a CC BY resource. Our plan for maintenance is to enable preprint platforms to update their listings on demand, pending verification of publically accessible information by ASAPbio staff. We will periodically archive the database in Zenodo to preserve prior versions.

Due to the lack of formal external peer review for many platforms (with the exception of those platforms that follow the F1000 Research Ltd model), preprint platforms that include medical content have been criticised as they may lack quality which can lead to errors in methods, results and interpretation, which subsequently has the potential to harm patients [19, 20]. This review has demonstrated the reality that many preprints do undergo some checks before going online, in contrast to the perception that preprints are not reviewed at all. Research Square, bioRxiv and medRxiv check specifically if there is potential harm to the preprints' dissemination before peer

For peer review only

review. Research Square also offers a transparent checklist to indicate the status of various quality assurance checks (not equivalent to scientific peer review) for each preprint.

Empirical evidence to support the use of editors and peer reviewers as a mechanism to ensure the quality of biomedical research is relatively weak [21, 22] although other studies have rendered peer review as being potentially useful [23, 24]. This review provides some justification that preprint platforms might be a reasonable option for researchers, especially given the time spent and associated cost of peer review [25]. In a recent survey of authors that have published with F1000 Research, 70% of respondents found the speed of publication to be important or very important [26]. In some scenarios, the time to deliver research findings may be as equally as important as research quality, and may be critical to health care provision. A good example of this is the current outbreak of novel coronavirus, where much of the preliminary evidence has been made available through preprints at the time of the World Health Organisation declaring the epidemic a public health emergency [27]. The issue of preprints being available before peer review, and also the level of screening before a preprint is posted, has been particularly pertinent in this case. As an example, bioRxiv has rapidly adapted to ensure users appreciate there has not been any peer review of the COVID-19-related work presented on this platform. In light of COVID-19, people including the patients and the public might be interested in a quick and easy way to search across platforms. As a start at improving discoverability, Europe PMC aggregates preprints from several repositories and already nearly 3000 preprint articles with ‘COVID-19’ in the title are listed [28].

Strengths and limitations of the study

The strength of this study is that we developed robust methodology for systematically identifying relevant preprint platforms and involved platform owners/representatives wherever possible to verify data that was either unclear or not available on platform websites, and when this was not possible, a second researcher was involved in the data acquisition process. Systematically identifying web-based data that is not indexed in an academic bibliographic database is challenging [29], though the methods employed here are compatible with the principles of a systematic search: the methods are transparent and reproducible. This approach builds on an earlier list of preprint servers [12], the process behind which did not use systematic methods or involve platform owners as far as we are aware.

For peer review only - http://bmjopen.bmj.com/site/about/guidelines.xhtml

We undertook an internal pilot of developing and testing out the data collection form in collaboration with a preprint platform owner and ASAPbio staff and funders (promoters of preprint use) in order to ensure that the list of characteristics collected was both complete and relevant to different stakeholder groups including academics and funders. Many of the general policy information for some platforms was not well-reported or easy to find online and therefore an unexpected but positive by-product of this research is that several of these platforms have updated their webpages to improve the visibility and transparency of their policies in response to this research. Similarly, some platforms became aware of policy attributes that they had not previously considered and are now in the process of considering these for future implementation.

One limitation is that we focussed our attention on the 'main' preprint article although in some cases different policies existed for the supplementary material, e.g. acceptable formats and licensing options. This level of detail will be included in our searchable database. Another potential shortcoming was that some preprint platforms had a partner journal and without verification it was sometimes unclear if the policy information related to the journal, preprint platform or both. Finally, we defined preprint platforms as hosting work before peer review is formally complete and we acknowledge that some platforms included here also host content that has already been peer-reviewed and/or published in a journal (e.g. post-prints) [30]; this is unlikely to affect the interpretation of policies for pre-printed works discussed herein.

Implications for authors of biomedical and medical research

With the increase in the number of preprint platforms available in the biomedical and medical research field, authors have the option to make publicly available and gain some early ownership of their research findings with little or no cost to themselves. Moreover, with many preprints platforms there is little restriction with regards to authors later publishing their preprints in peer-reviewed journals of their choice. While we did not tabulate information on this specifically, it was noted that some platforms (notably OSF platforms) did recommend that authors check the SHERPA/RoMEO service for details of a journal's sharing policy. There is also some evidence that pre-printing an article first may even boost citation rates [31] due to increased attention from tweets, blogs and news articles than those articles published without a preprint. With many platforms carrying out suitable quality-control checks and having long-term preservation strategies, preprint platforms offer authors direct control of the dissemination of their research in a rapid, free and open environment. As well as primary research, preprints are also vital to users of research (systematic reviewers and decision makers). As an example, a living mapping

For peer review only

systematic review of ongoing research into COVID-19 is currently being undertaken, and almost all included studies to date have been identified through preprint platforms [32].

Implications for preprint platforms

There has been a sharp rise in the number of preprints being published each month and it has been estimated (as of June 2019), preprints in biology represents approximately 2.4% of all biomedical publications [33]; and as of April 2020 there are already over 2.72 million preprints in the platforms that we evaluated. This review has summarised the key characteristics and policies of preprint platforms posting both medical and biomedical content although there is a need for some of these platforms to update their policies and to make them more transparent online. As preprints are not formally reviewed for scientific rigour through peer review, it is important to make it clear that their validity is less certain than for peer-reviewed articles (although even the latter may still not be valid). There is perhaps a growing need to standardise the checking process across platforms; such a process should not diminish the speed of publication (what authors value most about a preprint [22]). There is the temptation of making the checking process more rigorous, e.g. by including relevant researchers within the field as gatekeepers. However, this may slow down the process of making scientific work rapidly available and may promote groupthink, blocking innovative contrarian ideas to be circulated for public open review in the preprint platforms. Based on current checks, our review shows that most preprint platforms manage to post preprints within 48 hours and all within a week on average. Further challenges may arise on resources if the number of preprints continue to rise at a similar rate and the number of new platforms begins to plateau. And now, as several initiatives progress with work to build scientific review directly onto preprints (e.g. Peer Community in [34], Review Commons [35], PREreview [36]), it may become even more important to provide clarity about the level of checks a manuscript has already received and would need to receive to be considered “certified” by the scientific community. If anything, the wide public availability of preprints allows for far more extensive review by many reviewers, as opposed to the typical journal peer-review where only a couple of reviewers are involved. Our review identified 14 platforms linked to for-profit publishers and companies but only F1000 Research currently charges a small article processing charge to authors. With the increase in demand and resources needed to maintain preprint platforms, we should be mindful that article processing charges may change downstream meaning that platforms may have to charge authors.

Conclusion

One outcome of this review has been to understand the various drivers behind the proliferation of preprint platforms for the life and biomedical sciences. While arXiv, bioRxiv, chemRxiv and medRxiv aim to provide dedicated servers for academics within each field they are dedicated to, several academic groups have offered alternative subject-specific or regional services in line with their own community's needs, such as sharing work in languages other than English, using the OSF infrastructure. A third provider of preprint platforms is industry stakeholders: as academic publishers providing or acquiring preprint services to support the content they receive as submissions to their journals, and as biotechnology or pharmaceutical companies looking to support the sharing of relevant research content. Whether any platform becomes dominant may be influenced by the communities who adopt them, the influencers who promote them (funders and researchers who influence hiring and promotion decisions) and the financial sustainability underpinning them. We hope that enabling transparency into the processes and policies at each platform empowers the research community (including researchers, funders and others involved in the enterprise) to identify and support the platform(s) that help them to share research results most effectively.

Acknowledgments

We thank John Inglis for his advice on developing the data collection form and helpful comments on the manuscript. We also thank Robert Kiley, Geraldine Clement-Stoneham, Michael Parkin, Amy Riegelman, and Claire Yang for helpful feedback and conversations. We also would like to thank collectively the preprint platform owners and representatives who provided both data and verified information.

Contributors

JJK and DM jointly conceived the study and are the guarantors. JJK, DM and NP designed the study methods and developed the data collection form. JJK, NP, FM, IB, JPAI, JKP and DM were involved in identifying eligible platforms. JJK, NP and FM were all involved in data extraction and JJK and NP did the analysis and prepared the data tables. JKP developed the online searchable database. JJK prepared the initial manuscript. JJK, NP, FM, IB, JPAI, JKP and DM were involved in the revision of this manuscript. JJK, NP, FM, IB, JPAI, JKP and DM read and approved the final manuscript and are accountable for all aspects of the work, including the accuracy and integrity.

Funding

NP, FM and JKP received funding for ASAPbio preprint research from The Wellcome Trust, Chan Zuckerberg Initiative, Howard Hughes Medical Institute, Simons Foundation, Medical Research Council, and Canadian Institutes of Health Research.

Competing Interests

JKP is Executive Director of ASAPbio.

Data sharing statement

The data from this study are available in Zenodo (https://zenodo.org/record/3700874), which we will update periodically with a new version number as new platforms come online and policies of platforms currently identified change.

For peer review only

References

[1] *NIH enables investigators to include draft preprints in grant proposals* [ONLINE]. Available at: http://www.sciencemag.org/news/2017/03/nih-enables-investigators-include-draft-preprints-grant-proposals. [Accessed 8 April 2020].

[2] *REF 2021: Guidance on submissions* [ONLINE]. Available at: https://www.ref.ac.uk/media/1092/ref-2019_01-guidance-on-submissions.pdf. [Accessed 8 April 2020].

[3] *arXiv* [ONLINE]. Available at: https://arxiv.org/. [Accessed 8 April 2020].

[4] *ASAPbio* [ONLINE]. Available at: http://asapbio.org/preprint-info. [Accessed 8 April 2020].

[5] *bioRxiv* [ONLINE]. Available at: https://www.biorxiv.org/. [Accessed 20 April 2020].

[6] *medRxiv* [ONLINE]. Available at: https://www.medrxiv.org/. [Accessed 21 April 2020].

[7] *Center for Open Science* [ONLINE]. Available at: https://cos.io/. [Accessed 8 April 2020].

[8] *INLEXIO: The rising tide of preprint servers* [ONLINE]. Available at: https://www.inlexio.com/rising-tide-preprint-servers/. [Accessed 8 April 2020].

[9] *F1000 Research Ltd* [ONLINE]. Available at https://f1000research.com/. [Accessed 8 April 2020].

[10] *SciELO Preprints* [ONLINE]. Available at: https://preprints.scielo.org/index.php/scielo. [Accessed 8 April 2020].

[11] *Committee on Publication Ethics. Discussion document on preprints* [ONLINE]. Available at: https://publicationethics.org/files/u7140/COPE_Preprints_Mar18.pdf [Accessed 8 April 2020].

[12] *Martyn Rittman Research Preprints: Server List* [ONLINE]. Available at: https://docs.google.com/spreadsheets/d/17RgfuQcGJHKSsSJwZZn0oiXAnimZu2sZsWp8Z6ZaYYo/edit#gid=0 [Accessed 8 April 2020].

[13] *U.S National Library of Medicine: Factsheet Medline® Journal Selection* [ONLINE]. Available at: https://www.nlm.nih.gov/lstrc/jsel.html [Accessed 8 April 2020].

[14] *Zenodo: Practices and policies of preprint platforms for life and biomedical sciences* [ONLINE]. Available at: https://zenodo.org/record/3700874. [Accessed 8 April 2020].

[15] *Reporting Preprints and Other Interim Research Products* [ONLINE]. https://grants.nih.gov/grants/guide/notice-files/not-od-17-050.html [Accessed 8 April 2020].

[16] *Open Anthropology Research Repository* [ONLINE]. Available at: https://www.openanthroresearch.org/. [Accessed 8 April 2020].

For peer review only

[17] *Cambridge Open Engage* [ONLINE]. Available at: https://www.cambridge.org/engage/coe/public-dashboard. [Accessed 8 April 2020].

[18] *PeerJ blog: PeerJ Preprints to stop accepting new preprints Sep 30th 2019* [ONLINE]. Available at: https://peerj.com/blog/post/115284881747/peerj-preprints-to-stop-accepting-new-preprints-sep-30-2019/. [Accessed 8 April 2020].

[19] Annesley T, Scott M, Bastian H, Fonseca V, Ioannidis JPA, Keller MA et al. Biomedical journals and preprint services: friends or foes? *Clinical Chemistry* 2017; **63**(2): 453-458.

[20] Chalmers I and Glasziou P. Should there be greater use of preprint servers for publishing reports of biomedical science? *F1000Research* 2016; 5:272.

[21] Jefferson T, Rudin M, Brodney Folse S, Davidoff F. Editorial peer review for improving the quality of reports of biomedical studies. *Cochrane Database of Systematic Reviews* 2007, Issue 2. Art. No.: MR000016.

[22] Bruce R, Chauvin A, Trinquart L, Ravaud P, Boutron I. Impact of interventions to improve the quality of peer review of biomedical journals: a systematic review and meta-analysis. *BMC Medicine* 2016; **14**:85.

[23] Hopewell S, Collins GS, Boutron I, Yu L-M, Cook J, Shanyinde M et al. Impact of peer review on reports of randomised trials published in open peer review journals: retrospective before and after study. *BMJ* 2014; 349:g4145.

[24] Carneiro CFD, Queiroz VGS, Moulin TC, Carvalho CAM, Haas CB, Rayêe D, Henshall DE et al. Comparing quality of reporting between preprints and peer-reviewed articles in the biomedical literature. *BioRxiv* 2020 doi: https://doi.org/10.1101/581892.

[25] Kovanis M, Porcher R, Ravaud P, Trinquart L. The global burden of journal peer review in the Biomedical Literature: Strong Imbalance in the Collective Enterprise. *PLOS ONE* 2016; 11(11): e0166387.

[26] Kirkham J, Moher D. Who and why do researchers opt to publish in post-publication peer review platforms? - findings from a review and survey of F1000 Research. *F1000 Research* 2018, 7:920.

[27] *World Economic Forum: Coronavirus and the risks of 'speed science'* [ONLINE]. Available at: https://www.weforum.org/agenda/2020/03/speed-science-coronavirus-covid19-research-academic. [Accessed 14 April 2020].

[28] *Preprints in Europe PMC: reducing friction for discoverability* [ONLINE]. Available at: http://blog.europepmc.org/2018/07/preprints.html. [Accessed 14 April 2020].

[29] Stansfield C, Dickson K, Bangpan M. Exploring issues in the conduct of website searching and other online sources for systematic reviews: how can we be systematic? *Systematic Reviews* 2016; **5**:191.

[30] scholcommlab: Analyzing preprints: The challenges of working with OSF metadata. Available at: https://www.scholcommlab.ca/2019/09/11/preprints-challenges-part-two/ [Accessed 14 April 2020].

For peer review only

[31] *Nature Index: Preprints boost article citations and mentions* [ONLINE]. Available at: https://www.natureindex.com/news-blog/preprints-boost-article-citations-and-mentions [Accessed 14 April 2020].

[32] Living mapping and living systematic review of Covid-19 studies [ONLINE]. Available at: https://covid-nma.com/ [Accessed 14 April 2020].

[33] *ASAPbio: Biology preprints over time* [ONLINE]. Available at: https://asapbio.org/preprint-info/biology-preprints-over-time [Accessed 13 February 2020].

[34] *Peer Community in* [ONLINE]. Available at: https://peercommunityin.org/ [Accessed 14 April 2020].

[35] *Review COMMONS* [ONLINE]. Available at: https://www.reviewcommons.org/ [Accessed 14 April 2020].

[36] *PREREVIEW* [ONLINE]. Available at: https://content.prereview.org/ [Accessed 14 April 2020].

Figure 1: Flow diagram of included preprint platforms covering biomedical and medical scope

Table 1: Scope and ownership of preprint platforms

Table 2: Submission, journal transfer options and external discoverability

Table 3: Screening, moderation, and permanence of content

Table 4: Usage metrics and other features

Table 5: Metadata

S1_Table.docx: Reasons for excluded preprint platforms

For peer review only

Table 1: Scope and ownership of preprint platforms

Preprint Server (date launched/content first posted; number of articles[a])	Platform Description	Scope	Ownership (O)/Ownership Type (OT)/For Profit status (P)/Technology (T; name & openness of source code)	Time to publication (T) / Processing Charges (C)
**OSF Communities**				
AfricArxiv [1] (22 June 2018; 103) *Verified*	"..is a free, open source and community-led digital archive for African research"	All scientific fields	O[b]: Small group of enthusiasts<br>OT: Academic community group; charity<br>P: Non-profit or not-for-profit<br>T: Open Science Framework (open source)	T: A few days<br>C: No fee to author
AgriXiv [2] (15 February 2017; 418) *Verified*	"Preprints for Agriculture and Allied Sciences"	Relating to agriculture and allied sciences, including life sciences, medicine and health sciences, social and behavioural sciences	O[b]: Open Access India and small group of enthusiasts<br>OT: Academic community group<br>P: Non-profit or not-for-profit<br>T: Open Science Framework (open source)	T: Unknown<br>C: No fee to author
Arabixiv [3] (2 July 2018; 424) *Verified*	"The Arabic Preprint Server"	All scientific fields	O[b]: Small group of enthusiasts<br>OT: Academic community group<br>P: Non-profit or not-for-profit<br>T: Open Science Framework (open source)	T: Up to 48 hours<br>C: No fee to author
EcoEvoRxiv [4] (21 March 2018; 191) *Verified*	"A free preprint service for ecology, evolution and conservation"	Subject-specific, including ecology, evolution and conservation	O[b]: Transparency in Ecology and Evolution<br>OT: Academic community group<br>P: Non-profit or not-for-profit<br>T: Open Science Framework (open source)	T: Up to 48 hours<br>C: No fee to author
FocUS Archive [5] (20 September 2017; 42) *Verified*	"A free preprint service for the focused ultrasound research community"	Relating to ultrasound, including life sciences, medicine and health sciences	O[b]: Focused Ultrasound Foundation<br>OT: Funding organisation (funder); medical research organisation<br>P: Non-profit or not-for-profit<br>T: Open Science Framework (open source)	T: Unknown<br>C: No fee to author
Frenxiv [6] (06 July 2018; 94) *Verified*	"The French server for Preprints in all the scientific fields"	All scientific fields	O[b]: Small group of enthusiasts<br>OT: Individual or community<br>P: Non-profit or not-for-profit<br>T: Open Science Framework (open source)	T: A few days<br>C: No fee to author
INA-Rxiv [7] (17 August 2017; 16,637) *Verified*	"A preprint server for Indonesian academia to provide an open, free and sustainable scientific repository"	All scientific fields	O[b]: Indonesia open science team<br>OT: Academic community group<br>P: Non-profit or not-for-profit<br>T: Open Science Framework (open source)	T: Unknown<br>C: No fee to author
MarXiv [8] (09 November 2017; 449) *note: no longer on the OSF* *Verified*	"The free research repository for the ocean and marine-climate sciences"	Relating to ocean and marine climate sciences, including life sciences, social and behavioural sciences	O[b]: Open Communications for the Ocean (OCTO group)<br>OT: Publishing organisation<br>P: For-profit<br>T: Open Science Framework (open source)	T: Up to 48 hours<br>C: No fee to author
MetaArXiv [9] (17 March 2017; 121) *Verified*	"An interdisciplinary archive of articles focused on improving research transparency and reproducibility"	Relating to meta-science	O[b]: The Berkeley Initiative for Transparency in the Social Sciences (BITSS), Centre for Effective Global Action, University of California, Berkeley<br>OT: Academic institution<br>P: Non-profit or not-for-profit	T: Unknown<br>C: No fee to author

			T: Open Science Framework (open source)	
MindRxiv [10]<br>(17 July 2017; 192)<br>*Verified*	"Open archive for research on mind and contemplative practices"	Relating to mind and contemplative practices, including medicine and health sciences, neuroscience and neurobiology, psychology, social and behavioural sciences	O[b]: Mind and Life Institute<br>OT: Academic institution<br>P: Non-profit or not-for-profit<br>T: Open Science Framework (open source)	T: Same day<br>C: No fee to author
NutriXiv [11]<br>(11 September 2017; 63)<br>*Verified*	"A free preprint service for the nutritional sciences"	Relating to nutritional sciences	O[b]: Small group of enthusiasts<br>OT: Academic Institution<br>P: Non-profit or not-for-profit<br>T: Open Science Framework (open source)	T: Unknown<br>C: No fee to author
OSF Preprints [12]<br>(23 May 2007; 15,174)<br>*Verified*	"OSF Preprints is a preprints discovery site, where you can search not only the preprints in all subjects hosted at OSF Preprints, but also preprints on partner sites such as arXiv, AgriXiv, engrXiv, PsyArXiv and SocArXiv"	All scientific fields	O[b]: Center for Open Science<br>OT: Charity<br>P: Non-profit or not-for-profit<br>T: Open Science Framework (open source)	T: Unknown<br>C: No fee to author
PaleorXiv [13]<br>(18 August 2017; 162)<br>*Verified*	"…is a free, open source and community-led digital archive for Paleontology research"	Relating to palaeontology and paleobiology research	O[b]: Small group of enthusiasts<br>OT: Academic community group<br>P: Non-profit or not-for-profit<br>T: Open Science Framework (open source)	T: A few days<br>C: No fee to author
PsyArXiv [14]<br>(09 December 2016; 8,509)<br>*Verified*	"…is designed to facilitate rapid dissemination of psychological research"	Relating to psychology and psychological sciences	O[b]: Society for the Improvement of Psychological Science (SIPS)<br>OT: Scientific society<br>P: Non-profit or not-for-profit<br>T: Open Science Framework (open source)	T: A few days<br>C: No fee to author
SocArxiv [15]<br>(16 June 2017; 5,149)<br>*Verified*	"…open archive of the social sciences"	Relating to social sciences including social and behavioural sciences	O[b]: University of Maryland, College Park (UMD)<br>OT: Academic institution<br>P: Non-profit or not-for-profit<br>T: Open Science Framework (open source)	T: Unknown<br>C: No fee to author
SportRxiv [16]<br>(01 April 2017; 173)<br>*Verified*	"… is the first community-led and open access subject repository dedicated to sport, exercise, performance, and health research"	Relating to sports and exercise science, including rehabilitation and therapy, theatre, dance, physiology, physiotherapy, psychology, sociology	O[b]: Society for Transparency, Openness, and Replication in Kinesiology (STORK)<br>OT: Scientific society<br>P: Non-profit or not-for-profit<br>T: Open Science Framework (open source)	T: A few days<br>C: No fee to author
Thesis Commons [17]<br>(21 April 2017; 583)<br>*Verified*	"An open archive of theses"	All scientific fields	O[b]: Center for Open Science and small group of enthusiasts<br>OT: Academic community group; charity<br>P: Non-profit or not-for-profit<br>T: Open Science Framework (open source)	T: Unknown<br>C: No fee to author
**Open Research Central infrastructure**				
AAS Open Research [18]<br>(18 April 2018; 61)<br>*Verified*	"…is a platform for rapid publication and open peer review for researchers supported by AAS (African Academy of Sciences) and programs supported through its funding platform, AESA (Accelerating Excellence in Science in Africa's)"	Multiple scientific fields, including health and wellbeing[c]	O: African Academy of Sciences<br>OT: Funding organisation (funder)<br>P: Non-profit or not-for-profit<br>T: Custom F1000 software (closed source)	T: Up to 1 week<br>C: No fee to author (APC between £120-£800 typically paid by AAS)

AMRC Open Research [19] (19 February 2019; 7) *Verified*	"A platform for rapid author-led publication and open peer review of research funded by AMRC (Association of Medical Research Charities) member charities"	Broad life & biomedical research, including basic scientific, translational, applied and clinical research	O: Association of Medical Research Charities<br>OT: Funding organisation (funder), membership organisation<br>P: Non-profit or not-for-profit<br>T: Custom F1000 software (closed source)	T: Up to 1 week<br>C: Author fee applies (min £120; max £800[d]; £20 per poster, slide or document)
Gates Open Research [20] (06 November 2017; 218) *Verified*	"…is a platform for rapid author-led publication and open peer review of research funded by the Bill & Melinda Gates Foundation"	Global health, global development, global growth and opportunity	O: Bill and Melinda Gates Foundation<br>OT: Funding organisation (funder)<br>P: Non-profit or not-for-profit<br>T: Custom F1000 software (closed source)	T: Up to 1 week<br>C: No fee to author (APC between \$150-\$1500[d] paid by the Gates Foundation)
HRB Open Research [21] (28 February 2018; 80) *Verified*	"…is a platform for HRB-funded (Health Research Board) researchers to rapidly publish their research outputs in an open and accessible way"	Broad life & biomedical research, including exploratory, translational, biomedical, clinical, epidemiological, health services research	O: Health Research Board Ireland<br>OT: Funding organisation (funder)<br>P: Non-profit or not-for-profit<br>T: Custom F1000 software (closed source)	T: Up to 1 week<br>C: No fee to author (APC between €123-€818[d] paid by HRB)
MNI Open Research [22] (05 December 2017; 11) *Verified*	"…is a platform for rapid publication and open peer review of research conducted by MNI (Montreal Neurological Institute) and Hospital researchers"	Subject-specific, including brain imaging, cellular and molecular neuroscience, cognitive neuroscience, neurological diseases/disorders	O: Montreal Neurological Institute and Hospital, McGill University<br>OT: Academic institution<br>P: Non-profit or not-for-profit<br>T: Custom F1000 software (closed source)	T: Up to 1 week<br>C: No fee to author (APC between £116-£775[e] paid by MNI)
Wellcome Open Research [23] (15 November 2016; 602) *Verified*	"A new way for Wellcome-funded researchers to rapidly publish any results they think are worth sharing"	Broad life & biomedical research, including basic scientific, translational and clinical research	O: Wellcome Trust<br>OT: Funding organisation (funder)<br>P: Non-profit or not-for-profit<br>T: Custom F1000 software (closed source)	T: Up to 1 week<br>C: No fee to author (APC between £116-£775[d] paid by Wellcome)
**Others**				
arXiv [24] (14 August 1991; 1,688,374) *Verified*	"Open access to…e-prints…"	Multiple scientific fields, including quantitative biology[c]	O: Cornell University<br>OT: Academic institution<br>P: Non-profit or not-for-profit<br>T: SWORD (open source)	T: A few days<br>C: No fee to author
Authorea [25] (06 August 2013; 2,579) *Verified*	"…is the leading collaborative platform to read, write, and publish research"	All scientific fields	O: Atypon (Wiley)<br>OT: Publishing organisation (publisher)<br>P: For-profit<br>T: Custom Authorea software (proprietary software with some open source components)	T: Same day<br>C: No fee to author
bioRxiv [26] (7 November 2013; 79,370) *Verified*	"…is a free online archive and distribution service for unpublished preprints in the life sciences…"	Broad life & biomedical research (from animal behaviour and cognition to zoology)	O: Cold Spring Harbor Laboratory (CSHL)<br>OT: Academic institution<br>P: Non-profit or not-for-profit<br>T: Highwire Preprint & BenchPress (proprietary software with some open source components)	T: Up to 48 hours<br>C: No fee to author
Cell Press Sneak Peek [27] (02 April 2018; 2,152) *Verified*	"…is an author opt-in preview of the papers under review in [Cell Press] primary research journals"	Subject-specific, including cancer; stem cell; neuron; cell development; biology; immunity	O: Elsevier<br>OT: Publishing organisation (publisher)<br>P: For-profit<br>T: Unknown	T: A few days<br>C: No fee to author
ChemRxiv [28] (15 August 2017; 4,332)	"…is a free submission, distribution and archive service for unpublished preprints in chemistry and related areas"	Subject-specific, including biological and medicinal chemistry	O: American Chemical Society, German Chemical Society, and Royal Society of Chemistry<br>OT: Scientific society<br>P: Non-profit or not-for-profit	T: Up to 48 hours<br>C: No fee to author

For peer review only

			T: Figshare (closed source)	
ChinaXiv [29]<br>(25 January 2016; 13,561)	"…is an open repository of Chinese scientific research papers in the field of natural sciences"	All scientific fields	O: Chinese Academy of Sciences<br>OT: Academic institution<br>P: Non-profit or not-for-profit<br>T: Java, SQL server (closed source)	T: Unknown<br>C: No fee to author
Earth and Space Science Open Archive (ESSOAr) [30]<br>(11 February 2018; 671)<br>*Verified*	"…is a community server established to accelerate the open discovery and dissemination of Earth and space science"	Relating to earth, environmental, and space sciences (subject-specific topics include biology, microbiology and public health)	O: American Geophysical Union (AGU), Wiley<br>OT: Scientific society; publishing organisation (publisher)<br>P: Non-profit or not-for-profit<br>T: Atypon/Literatum (closed source)	T: A few days<br>C: No fee to author
F1000 Research [31]<br>(13 July 2012; 3,226) – Verified	"…is an Open Research publishing platform for life scientists, offering immediate publication of articles and other research outputs without editorial bias"	Broad life & biomedical research, including basic scientific, translational and clinical research within the life sciences and medicine	O: F1000 Research Ltd.<br>OT: Publishing organisation (publisher)<br>P: For-profit<br>T: Custom F1000 software (closed source)	T: Up to 1 week<br>C: Author fee applies (min $150; max $2000 US)[d]
JMIR Preprints [32]<br>(11 November 2015; Not available)	"A preprint server for pre-publication/pre-peer-review preprints intended for community review as well as ahead-of-print (accepted) manuscripts"	Broad life & biomedical research	O: JMIR Publications Inc.<br>OT: Publishing organisation (publisher)<br>P: Unknown<br>T: Unknown	T: Unknown<br>C: No fee to author
medRxiv [33]<br>(25 June 2019; 3,412)<br>*Verified*	"…is a free online archive and distribution server for complete but unpublished manuscripts (preprints) in the medical, clinical, and related health sciences"	Broad medical, clinical and related health sciences	O: Cold Spring Harbor Laboratory (CSHL), Yale University and BMJ<br>OT: Academic institution<br>P: Non-profit or not-for-profit<br>T: Highwire Preprint & BenchPress (proprietary software with some open source components)	T: A few days<br>C: No fee to author
MitoFit Preprint Archives [34]<br>(12 February 2019; 15)	"Open Access preprint server for mitochondrial physiology and bioenergetics"	Subject-specific, including mitochondrial physiology, bioenergetics, ergodynamics	O: Oroborus Instruments<br>OT: Life sciences tech company<br>P: Non-profit or not-for-profit<br>T: MediaWiki (open source)	T: Up to 1 week<br>C: No fee to author
NeuroImage: Clinical - *First Look* [35]<br>(01 August 2018; 12)<br>*Verified*	"…is an author opt-in preview of the papers currently under consideration in [NeuroImage: Clinical]"	Subject-specific, including diseases, disorders and syndromes involving the nervous system, neuroimaging	O: Elsevier<br>OT: Publishing organisation (publisher)<br>P: For-profit<br>T: Unknown	T: A few days<br>C: No fee to author
PeerJ Preprints [36]<br>(04 April 2013, 5,068; *note: no new preprints accepted after 30 September 2019*)<br>*Verified*	"…is the 'pre-print' area of PeerJ"	Biological, medical and environmental sciences, matching scope to *PeerJ* journal (excludes Clinical Trials, diagnostic, therapeutic or health implications)	O: PeerJ<br>OT: Publishing organisation (publisher)<br>P: For-profit<br>T: Custom PeerJ Preprints software (closed source)	T: Up to 24 hours<br>C: No fee to author
Preprints with The Lancet [37]<br>(30 June 2018; 5,073)<br>*Verified*	"...a place where journals and other research experts identify content of interest prior to publication...Authors have either opted in at submission to The Lancet family of journals to post their preprints on Preprints with The Lancet, or submitted directly via SSRN"	Medical sciences	O: Elsevier<br>OT: Publishing organisation (publisher)<br>P: For-profit<br>T: Unknown	T: Up to 2 weeks<br>C: No fee to author

Preprints.org [38]<br>(02 July 2016; 14,837)	"…is a multidisciplinary preprint platform that makes scientific manuscripts from all fields of research immediately available"	All scientific fields	O: Multidisciplinary Digital Publishing Institute (MDPI)<br>OT: Publishing organisation (publisher)<br>P: Non-profit or not-for-profit<br>T: Custom software (closed source)	T: Up to 24 hours<br>C: No fee to author
Research Square [39]<br>(15 October 2018; 20,917)<br>*Verified*	"...lets you share your work early, gain feedback from the community, and start making changes to your paper prior to peer review in a journal"	All scientific fields	O: Research Square<br>OT: Publishing services organisation<br>P: For-profit<br>T: Custom software (closed source)	T: A few days<br>C: No fee to author
SciELO Preprints [40]<br>(06 July 2018; 31)<br>*Verified*	"…focus on manuscripts related to the main topics of the SciELO (Scientific Electronic Library Online) 20 Years Week agenda"	Multiple scientific fields including biological, health, human, applied social sciences[c]	O: SciELO and FAPESP Program<br>OT: Research infrastructure program<br>P: Non-profit or not-for-profit<br>T: Open Journals System (open source)	T: A few days<br>C: No fee to author
SSRN [41]<br>(1994; 791,714)<br>*Verified*	"…is an open-access online preprint community providing valuable services to leading academic schools and government institutions…"	Multiple scientific fields including applied sciences, health sciences, life sciences, social sciences[c]	O: Elsevier<br>OT: Publishing organisation (publisher)<br>P: For-profit<br>T: Unknown	T: Up to 48 hours<br>C: No fee to author
Surgery Open Science - *First Look* [42]<br>(23 April 2019; 42)<br>*Verified*	"…open access journal affiliated with the journal *Surgery* and striving to incorporate the strongest tenets of the open science movement including allowing author preprint"	Relating to surgery, matching scope to *Surgery* journal	O: Elsevier<br>OT: Publishing organisation (publisher)<br>P: Unknown<br>T: Unknown	T: A few days<br>C: No fee to author
Therapoid [43]<br>(08 February 2017; 9)<br>*Verified*	"an open web platform for scientific collaboration by Open Therapeutics"	Relating to pharmaceutical, medical device, and medical/health diagnostic technologies	O: Open Therapeutics<br>OT: Life sciences tech company<br>P: For-profit<br>T: Custom software (proprietary software with some open source components)	T: Same day<br>C: No fee to author
ViXra [44]<br>(25 February 2007; 35,405)	"An alternative archive… in Science and Mathematics serving the whole scientific community"	Multiple scientific fields, including biology[c]	O: Small group of enthusiasts<br>OT: Individual or community<br>P: Non-profit or not-for-profit<br>T: Custom viXra software with services from formsite.com and disqus.com (closed source)	T: Up to 48 hours<br>C: No fee to author

APC: Article Processing Charge

[a] As of 21st April 2020 (accept for SSRN, last verified 11th September 2019); [b] OSF communities are still working out the balance of ownership between service leads and the Centre for Open Science; [c] Disciplinary scope of server is broader – we list only those relevant to biomedical and health sciences; [d] Based on article length, surcharges may apply for articles with unusually large word counts; [e] MNI will pay for one publication per lab only.

For peer review only

Table 2: Submission, journal transfer options and external discoverability

Preprint Server	Major content accepted (level of research[a], where known)	Content Language(s)	Permitted submission formats (S)<br>Viewing options (V)<br>Unique identifier (I; if DOI, prefix in brackets)<br>Versioning policy / version identifiers (VP)<br>Licensing options (L)<br>Reader registration required to view or download full content (R)	Journal submission options<br>Preprint displays link to journal publication (PL)	External content indexing (EI) / Metadata availability (MA) / Machine-readable full-text content (MR)
**OSF Communities**					
AfricArxiv [1] – *Verified*	Any scholarly content	Afrikaans, Akan, English, French, Igbo, Swahili, Zulu, other unspecified native African languages – summary must be provided in English and French, non-English languages held in moderation queue awaiting verification	S: PDF, Word doc, other text formats<br>V: By download in file format submitted by author(s), PDF viewable in browser without download<br>I: DOI (10.31730/osf.io)<br>VP: Accepts any new versions / single DOI retained for all versions<br>L: Authors are provided with a choice of licenses: GNU Lesser General Public License (LGPL) 3.0, BSD 3-Clause "New"/"Revised" License, BSD 2-Clause "Simplified" License, GNU Lesser General Public License (LGPL) 2.1, CC-By Attribution 4.0 International, Artistic License 2.0, CC0 1.0 Universal, Apache License 2.0, Mozilla Public License 2.0, Academic Free License (AFL) 3.0, Eclipse Public License 1.0, MIT License, GNU General Public License (GPL) 3.0, GNU General Public License (GPL) 2.0<br>R: No reader registration required	*Journal submission:* Unrestricted - manuscript can be published in any destination<br><br>*Journals directly associated with platform:* None<br><br>*Facilitated submission to other journals:* None<br><br>PL: Can be added by author	EI: Google Scholar, SHARE, Microsoft Academic, Unpaywall<br><br>MA: Openly available via API (application programming interface)<br><br>MR: No (planned in development roadmap)
AgriXiv [2] – *Verified*	Research manuscripts only	Cyrillic, English, Indonesian, unknown if others	S: Any format supported by OSF platform<br>V: By download in file format submitted by author(s), PDF viewable in browser without download<br>I: DOI (10.31220/osf.io)<br>VP: Accepts any new versions / single DOI retained for all versions<br>L: Authors are provided with a choice of licenses: CC0 1.0 Universal, CC-By Attribution 4.0 International<br>R: No reader registration required	*Journal submission:* Unrestricted - manuscript can be published in any destination<br><br>*Journals directly associated with platform:* None<br><br>*Facilitated submission to other journals:* None<br><br>PL: Can be added by author	EI: Google Scholar, SHARE, Microsoft Academic, Unpaywall<br><br>MA: Openly available via API<br><br>MR: No (planned in development roadmap)
Arabixiv [3] – *Verified*	Any scholarly content	Arabic, English	S: PDF<br>V: By download in file format submitted by author(s), PDF viewable in browser without download<br>I: DOI (10.31221/osf.io)<br>VP: Accepts any new versions / single DOI retained for all versions<br>L: Authors are provided with a choice of licenses: No license, GNU Lesser General Public License (LGPL) 3.0, BSD 3-Clause "New"/"Revised" License, BSD 2-Clause "Simplified" License, GNU Lesser General	*Journal submission:* Unrestricted - manuscript can be published in any destination<br><br>*Journals directly associated with platform:* None<br><br>*Facilitated submission to other journals:* None<br><br>PL: Can be added by author	EI: Google Scholar, SHARE, Microsoft Academic, Unpaywall<br><br>MA: Openly available via API<br><br>MR: No (planned in development roadmap)

			Public License (LGPL) 2.1, CC-By Attribution 4.0 International, Artistic License 2.0, CC0 1.0 Universal, Apache License 2.0, Mozilla Public License 2.0, Academic Free License (AFL) 3.0, Eclipse Public License 1.0, MIT License, GNU General Public License (GPL) 3.0, GNU General Public License (GPL) 2.0<br>R: No reader registration required		
EcoEvoRxiv [4] – *Verified*	All article types, datasets (primary and secondary research)	English	S: PDF<br>V: By download in file format submitted by author(s), PDF viewable in browser without download<br>I: DOI (10.32942/osf.io)<br>VP: Accepts any new versions / single DOI retained for all versions<br>L: Authors are provided with a choice of licenses: CC-By Attribution-ShareAlike 4.0 International, CC-By Attribution-NonCommercial-NoDerivatives 4.0 International, CC-By Attribution 4.0 International, CC0 1.0 Universal, CC-By Attribution-ShareAlike 4.0 International, CC-By Attribution-NonCommercial-NoDerivatives 4.0 International, with none preferred<br>R: No reader registration required	*Journal submission:* Unrestricted - manuscript can be published in any destination<br><br>*Journals directly associated with platform:* None<br><br>*Facilitated submission to other journals:* None<br><br>PL: Can be added by author	EI: Google Scholar, SHARE, Microsoft Academic, Unpaywall<br><br>MA: Openly available via API<br><br>MR: No (planned in development roadmap)
FocUS Archive [5] – *Verified*	Unknown	Unknown	S: Any format supported by OSF platform<br>V: By download in file format submitted by author(s), PDF viewable in browser without download<br>I: DOI (10.31225/osf.io)<br>VP: Accepts any new versions / single DOI retained for all versions<br>L: Authors must use CC-BY license<br>R: No reader registration required	*Journal submission:* Unrestricted - manuscript can be published in any destination<br><br>*Journals directly associated with platform:* None<br><br>*Facilitated submission to other journals:* None<br><br>PL: Can be added by author	EI: Google Scholar, SHARE, Microsoft Academic, Unpaywall<br><br>MA: Openly available via API<br><br>MR: No (planned in development roadmap)
Frenxiv [6] – *Verified*	All article types, datasets, posters (primary and secondary research)	Any language (including Arabic, English, French, Indonesian)	S: PDF, Word doc, LaTeX<br>V: By download in file format submitted by author(s), PDF viewable in browser without download<br>I: DOI (10.31226/osf.io)<br>VP: Accepts any new versions / single DOI retained for all versions<br>L: Authors are provided with a choice of licenses: GNU Lesser General Public License (LGPL) 3.0, BSD 3-Clause "New"/"Revised" License, BSD 2-Clause "Simplified" License, GNU Lesser General Public License (LGPL) 2.1, CC-By Attribution 4.0 International, Artistic License 2.0, CC0 1.0 Universal, Apache License 2.0, Mozilla Public License 2.0, Academic Free License (AFL) 3.0, Eclipse Public	*Journal submission:* Unrestricted - manuscript can be published in any destination<br><br>*Journals directly associated with platform:* None<br><br>*Facilitated submission to other journals:* None<br><br>PL: Can be added by author	EI: Google Scholar, SHARE, Microsoft Academic, Unpaywall<br><br>MA: Openly available via API<br><br>MR: No (planned in development roadmap)

			License 1.0, MIT License, GNU General Public License (GPL) 3.0, GNU General Public License (GPL) 2.0, No license<br>R: No reader registration required		
INA-Rxiv [7] – *Verified*	Multiple types (including research manuscripts, essays, datasets, multimedia files; types not accepted are: thesis, report, draft book)	English, Indonesian	S: PDF, Word doc, LaTeX, MarkDown<br>V: By download in file format submitted by author(s), PDF viewable in browser without download<br>I: DOI (10.31227/osf.io)<br>VP: Accepts any new versions / single DOI retained for all versions<br>L: Authors are provided with a choice of licenses: GNU Lesser General Public License (LGPL) 3.0, BSD 3-Clause "New"/"Revised" License, BSD 2-Clause "Simplified" License, GNU Lesser General Public License (LGPL) 2.1, CC-By Attribution 4.0 International, Artistic License 2.0, CC0 1.0 Universal, Apache License 2.0, Mozilla Public License 2.0, Academic Free License (AFL) 3.0, Eclipse Public License 1.0, MIT License, GNU General Public License (GPL) 3.0, GNU General Public License (GPL) 2.0, No license, with CC-BY preferred<br>R: No reader registration required	*Journal submission:* Unrestricted - manuscript can be published in any destination<br><br>*Journals directly associated with platform:* None<br><br>*Facilitated submission to other journals:* None<br><br>PL: Can be added by author	EI: Google Scholar, SHARE, Microsoft Academic, Unpaywall<br><br>MA: Openly available via API<br><br>MR: No (planned in development roadmap)
MarXiv [8] – *note: no longer on the OSF – Verified*	Any scholarly content	Unknown	S: PDF, LaTeX<br>V: By download in file format submitted by author(s), PDF viewable in browser without download<br>I: DOI (10.31230/osf.io)<br>VP: Accepts any new versions / single DOI retained for all versions<br>L: Authors are provided with a choice of licenses: CC-BY, CC-BY-NC-ND, No License, GNU Lesser General Public License (LGPL) 3.0, Eclipse Public License 1.0, CC-BY-SA; with CC-BY preferred<br>R: No reader registration required	*Journal submission:* Unrestricted - manuscript can be published in any destination<br><br>*Journals directly associated with platform:* None<br><br>*Facilitated submission to other journals:* None<br><br>PL: Can be added by author	EI: Google Scholar, SHARE, Microsoft Academic, Unpaywall, Altmetric, OpenChannels.org<br><br>MA: Openly available via API<br><br>MR: No (planned in development roadmap)
MetaArXiv [9] – *Verified*	Multiple types (including manuscripts, theses, conference proceedings, tutorials)	English	S: PDF, Word doc, WordPerfect, LaTeX<br>V: By download in file format submitted by author(s), PDF viewable in browser without download<br>I: DOI (10.31222/osf.io)<br>VP: Accepts any new versions / single DOI retained for all versions<br>L: Authors are provided with a choice of licenses: CC0 1.0 Universal, CC-By Attribution 4.0 International<br>R: No reader registration required	*Journal submission:* Unrestricted - manuscript can be published in any destination<br><br>*Journals directly associated with platform:* None<br><br>*Facilitated submission to other journals:* None<br><br>PL: Can be added by author	EI: Google Scholar, SHARE, Microsoft Academic, Unpaywall<br><br>MA: Openly available via API<br><br>MR: No (planned in development roadmap)
MindRxiv [10] – *Verified*	Research manuscripts only	English	S: Any format supported by OSF platform (PDF preferred)	*Journal submission:* Unrestricted - manuscript can be published in any destination	EI: Google Scholar, SHARE, Microsoft Academic, Unpaywall

			V: By download in file format submitted by author(s), PDF viewable in browser without download<br>I: DOI (10.31231/osf.io)<br>VP: Accepts any new versions / single DOI retained for all versions<br>L: Authors are provided with a choice of licenses: CC0, CC-BY, no license required; with CC0 preferred<br>R: No reader registration required	*Journals directly associated with platform:* None<br><br>*Facilitated submission to other journals:* None<br><br>PL: Can be added by author	MA: Openly available via API<br><br>MR: No (planned in development roadmap)
NutriXiv [11] – *Verified*	Unknown	Unknown	S: Any format supported by OSF platform<br>V: By download in file format submitted by author(s), PDF viewable in browser without download<br>I: DOI (10.31232/osf.io)<br>VP: Accepts any new versions / single DOI retained for all versions<br>L: Authors must use CC0 license<br>R: No reader registration required	*Journal submission:* Unrestricted - manuscript can be published in any destination<br><br>*Journals directly associated with platform:* None<br><br>*Facilitated submission to other journals:* None<br><br>PL: Can be added by author	EI: Google Scholar, SHARE, Microsoft Academic, Unpaywall<br><br>MA: Openly available via API<br><br>MR: No (planned in development roadmap)
OSF Preprints [12] – *Verified*	Multiple types (including research articles, posters, new data)	Any language	S: PDF, Word doc<br>V: By download in file format submitted by author(s), PDF viewable in browser without download<br>I: DOI (10.31219/osf.io)<br>VP: Accepts any new versions / single DOI retained for all versions<br>L: Authors are provided with a choice of licenses: CC0, CC-BY, no license; with no preference for which license chosen<br>R: No reader registration required	*Journal submission:* Unrestricted - manuscript can be published in any destination<br><br>*Journals directly associated with platform:* None<br><br>*Facilitated submission to other journals:* None<br><br>PL: Can be added by author	EI: Google Scholar, SHARE<br><br>MA: Openly available via API<br><br>MR: No (planned in development roadmap)
PaleorXiv [13] – *Verified*	Multiple article types (including systematic reviews, data papers, methods papers)	Any language – non-English languages held in moderation queue awaiting verification	S: PDF, LaTeX, text<br>V: By download in file format submitted by author(s), PDF viewable in browser without download<br>I: DOI (10.31233/osf.io)<br>VP: Accepts any new versions / single DOI retained for all versions<br>L: Authors must use CC-BY license<br>R: No reader registration required	*Journal submission:* Unrestricted - manuscript can be published in any destination<br><br>*Journals directly associated with platform:* None<br><br>*Facilitated submission to other journals:* None<br><br>PL: Can be added by author	EI: Google Scholar, SHARE, Microsoft Academic, Unpaywall<br><br>MA: Openly available via API<br><br>MR: No (planned in development roadmap)
PsyArXiv [14] – *Verified*	Any scholarly content	Unknown	S: PDF, Word doc<br>V: By download in file format submitted by author(s), PDF viewable in browser without download<br>I: DOI (10.31234/osf.io)<br>VP: Accepts any new versions / single DOI retained for all versions<br>L: Authors are provided with a choice of licenses: CC0, CC-BY, no license; with no preference stated<br>R: No reader registration required	*Journal submission:* Unrestricted - manuscript can be published in any destination<br><br>*Journals directly associated with platform:* None<br><br>*Facilitated submission to other journals:* None<br><br>PL: Can be added by author	EI: Google Scholar, SHARE, Microsoft Academic, Unpaywall<br><br>MA: Openly available via API<br><br>MR: No (planned in development roadmap)

For peer review only

SocArxiv [15] – *Verified*	Research manuscripts only	Unknown	S: Any format supported by OSF platform<br>V: By download in file format submitted by author(s), PDF viewable in browser without download<br>I: DOI (10.31235/osf.io)<br>VP: Accepts any new versions / single DOI retained for all versions<br>L: Authors are provided with a choice of licenses: CC0, CC-BY, no license; with CC0 or CC-BY preferred<br>R: No reader registration required	*Journal submission:* Unrestricted - manuscript can be published in any destination<br><br>*Journals directly associated with platform:* None<br><br>*Facilitated submission to other journals:* None<br><br>PL: Can be added by author	EI: Google Scholar, SHARE, Microsoft Academic, Unpaywall<br><br>MA: Openly available via API<br><br>MR: No (planned in development roadmap)
SportRxiv [16] – *Verified*	All article types (including research manuscripts, commentaries, review articles, current opinions, case studies, technical reports, short communications)	Any language – non-English languages held in moderation queue awaiting verification	S: PDF<br>V: By download in file format submitted by author(s), PDF viewable in browser without download<br>I: DOI (10.31236/osf.io)<br>VP: Accepts any new versions / single DOI retained for all versions<br>L: Authors are provided with a choice of licenses: CC0, CC-BY; with CC-BY preferred<br>R: No reader registration required	*Journal submission:* Unrestricted - manuscript can be published in any destination<br><br>*Journals directly associated with platform:* None<br><br>*Facilitated submission to other journals:* None<br><br>PL: Can be added by author	EI Google Scholar, SHARE, Microsoft Academic, Unpaywall<br><br>MA: Openly available via API<br><br>MR: No (planned in development roadmap)
Thesis Commons [17] – *Verified*	Theses only	Any language	S: PDF, Word doc<br>V: By download in file format submitted by author(s), PDF viewable in browser without download<br>I: DOI (10.31237/osf.io)<br>VP: Accepts any new versions / single DOI retained for all versions<br>L: Authors are provided with a choice of licenses: CC0, CC-BY; with no preference stated<br>R: No reader registration required	*Journal submission:* Unrestricted - manuscript can be published in any destination<br><br>*Journals directly associated with platform:* None<br><br>*Facilitated submission to other journals:* None<br><br>PL: Can be added by author	EI: Google Scholar, SHARE, Microsoft Academic, Unpaywall<br><br>MA: Openly available via API<br><br>MR: No (planned in development roadmap)
**Open Research Central infrastructure**					
AAS Open Research [18] – *Verified*<br><br>AMRC Open Research [19] – *Verified*<br><br>Gates Open Research [20] – *Verified*<br><br>HRB Open Research [21] – *Verified*<br><br>MNI Open Research [22] – *Verified*	All article types (primary and secondary research)<br><br>Editorials (Wellcome Open Research only)<br><br>Posters and slides (AAS, AMRC, Gates Open Research only)	English (articles)<br><br>Accepts posters and slides in all languages – metadata and description must be in English	S: Word doc, RTF (Wellcome Open Research also supports LaTeX via Overleaf template)<br>V: By PDF download, full-text viewable in browser without PDF (e.g. as HTML), by XML download<br>I: DOI (10.12688/aasopenres; …/amrcopenres; …/gatesopenres; …/hrbopenres; …/mniopenres; …/wellcomeopenres)<br>VP: Accepts any new versions / each version receives its own DOI and own non-DOI citable identifier<br>L: Authors must use CC-BY license<br>R: No reader registration required	*Journal submission:* Restricted - manuscript can only be published in journal linked to server (AAS/AMRC/Gates/HRB/MNI/Wellcome Open Research, respectively)<br><br>PL: Yes, added by platform within one month of publication	EI: Google Scholar, PrePubMed, Europe PMC, SciLit<br><br>MA: Available for free upon request (AAS/AMRC/Gates/HRB/MNI); Openly available via API (Wellcome Open Research)<br><br>MR: Yes (XML and HTML)

For peer review only

Wellcome Open Research [23] – *Verified*					
**Others**					
arXiv [24] – *Verified*	Research manuscripts only	Any language – abstract must be in English	S: PDF, LaTeX, HTML<br>V: PDF (and other format(s) as supplied by author) viewable in browser without download<br>I: Platform-specific ID (arXiv ID)<br>VP: Accepts any new versions / each version receives its own non-DOI citable identifier<br>L: Authors are provided with a choice of licenses: CC0, CC-BY, CC-BY-SA 4.0, CC-BY-NC-SA 4.0, non-exclusive license to distribute, any other CC license as specified in manuscript text; with no preference for which license chosen<br>R: No reader registration required	*Journal submission:* Unrestricted - manuscript can be published in any destination<br><br>*Journals directly associated with platform:* None<br><br>*Facilitated submission to other journals:* Not available<br><br>PL: Yes, added by platform after one month from publication	EI: Google Scholar, PrePubMed (q-bio only), Europe PMC, SciLit, SHARE, INSPIRE-HEP, The NASA Astrophysics Data System (ADS), The arXiv Search Interface from the National Science Library, Chinese Academy of Sciences (also in Chinese)<br><br>MA: Openly available via API<br><br>MR: Require full-text to be submitted in machine-readable format
Authorea [25] – *Verified*	Any scholarly content (including articles, datasets, code, figures, tables, slides, micropublications, and Jupyter Notebooks)	Any language	S: PDF, Word doc, LaTeX<br>V: Full-text viewable in browser without PDF (e.g. as HTML), by LaTeX download<br>I: DOI (10.22541)<br>VP: Accepts any new versions / each version receives its own DOI<br>L: Authors must use CC-BY license<br>R: No reader registration required	*Journal submission:* Unrestricted - manuscript can be published in any destination<br><br>*Journals directly associated with platform*: None<br><br>*Facilitated submission to other journals:* Yes, listed at https://support.authorea.com/en-us/article/how-do-i-directly-submit-to-a-publisher-atxzf7/<br><br>PL: Yes, added by platform within one month of publication	EI: SciLit, Crossref<br><br>MA: Unknown<br><br>MR: Yes (HTML)
bioRxiv [26] – *Verified*	Research manuscripts only (primary research only; white papers accepted in Scientific Communication and Education category only; types not accepted are: datasets, theses, figures, protocols, correspondence	English – non-English language translations can be included as supplementary files	S: PDF, Word doc, WordPerfect<br>V: By PDF download, PDF viewable in browser without download, full-text viewable in browser without PDF (e.g. as HTML)<br>I: DOI (10.1101)<br>VP: Accepts new versions until acceptance at journal / single DOI retained but new versions have distinct URLs<br>L: Authors are provided with a choice of licenses: CC0, CC-BY, CC-BY-NC, CC-BY-ND, CC-BY-NC-ND, or no reuse; with no preference for which license chosen<br>R: No reader registration required	*Journal submission:* Unrestricted - manuscript can be published in any destination<br><br>*Journals directly associated with platform:* None<br><br>*Facilitated submission to other journals:* Yes, listed at https://www.biorxiv.org/about-biorxiv<br><br>PL: Yes, added by platform within one month of publication	EI: Google Scholar, Microsoft Academic, Meta, EuropePMC, SHARE, Crossref<br><br>MA: Openly available via API<br><br>MR: Yes (HTML)

For peer review only

	without new data or analyses)				
Cell Press Sneak Peek[b] [27] – *Verified*	Content criteria as directed by associated journal(s) (Cell Press portfolio)	Unknown	S: PDF<br>V: By PDF download, PDF viewable in browser without download<br>I: URL only<br>VP: Unknown / Unknown<br>L: Unknown<br>R: Reader can view basic metadata without account but must register to view and download full content	*Journal submission:* Manuscripts must be submitted to a Cell Press Journal first, authors are then provided with the option of displaying their manuscript while under review using SSRN's 'First Look' (aka 'Sneak Peek')<br><br>PL: Unknown	EI: Unknown<br><br>MA: Unknown<br><br>MR: No
ChemRxiv [28]	Research manuscripts only	English	S: Any (PDF)<br>V: By PDF download, PDF viewable in browser without download<br>I: DOI (10.26434)<br>VP: Accepts new versions until acceptance at journal / each version receives its own DOI suffix<br>L: Authors are provided with a choice of licenses: CC-BY, CC-BY-NC, CC-BY-NC-ND; with CC-BY-NC-ND preferred<br>R: No reader registration required	*Journal submission:* Unrestricted - manuscript can be published in any destination<br><br>*Journals directly associated with platform:* None<br><br>*Facilitated submission to other journals:* Yes, journals owned by American Chemical Society, Royal Society of Chemistry, German Chemical Society (other publishers coming soon)<br><br>PL: Yes, added by platform within one month of publication	EI: Google Scholar, EuropePMC, Chemical Abstracts Services, Crossref<br><br>MA: Openly available via API<br><br>MR: If provided by author in machine-readable format
ChinaXiv [29]	Research manuscripts only	Standard Chinese, English, unknown if other	S: PDF, Word doc, LaTeX<br>V: By PDF download<br>I: DOI (10.12074)<br>VP: Accepts any new versions / single DOI retained for all versions<br>L: Authors are provided with a choice of licenses: CC0, CC-BY, CC-BY-SA, CC-BY-NC-SA; with no preference for which license chosen<br>R: No reader registration required	*Journal submission:* Unrestricted - manuscript can be published in any destination<br><br>*Journals directly associated with platform:* Multiple, listed at http://chinaxiv.org/journal/browse.htm<br><br>*Facilitated submission to other journals:* Not available<br><br>PL: Can be added by author or journal	EI: ROAR (Registry of Open Access Repositories)<br><br>MA: Openly available via API<br><br>MR: No
ESSOAr [30] – *Verified*	Scholarly research content (including manuscripts and posters)	English	S: PDF (Word and LaTeX in development)<br>V: By PDF download<br>I: DOI (10.1002)<br>VP: Accepts new versions until acceptance at journal / each version receives its own DOI suffix<br>L: Authors are provided with a choice of licenses: CC-BY, CC-BY-NC, CC-BY-NC-ND, non-exclusive license; with no preference stated<br>R: No reader registration required	*Journal submission:* Unrestricted - manuscript can be published in any destination<br><br>*Journals directly associated with platform:* 21 AGU publications (others in roadmap)<br><br>*Facilitated submission to other journals:* Not yet (on roadmap)<br><br>PL: Yes, added by platform within one month of publication; can be added by author or journal	EI: Google Scholar, SHARE, Crossref<br><br>MA: Openly available via Crossref<br><br>MR: Yes

For peer review only

F1000 Research [31] – *Verified*	Any scholarly content (including articles, posters and slides; primary and secondary research)	English	S: Word doc, RTF, LaTeX via Overleaf only<br>V: By PDF download, full-text viewable in browser without PDF, by XML download<br>I: DOI (10.12688/f1000research)<br>VP: Accepts any new versions / each version receives its own DOI and own non-DOI citable identifier<br>L: Authors must use CC-BY license<br>R: No reader registration required	*Journal submission:* Restricted - manuscript can only be published in journal linked to server (F1000 Research)<br><br>PL: Yes, added by platform within one month of publication	EI: Google Scholar, Europe PMC, SHARE, SciLit, PrePubMed<br><br>MA: Openly available via API<br><br>MR: Yes (XML and HTML)
JMIR Preprints [32]	Research manuscripts, blog posts, "grey" online reports	Unknown	S: Word doc<br>V: By PDF download, PDF viewable in browser without download<br>I: DOI (10.2196/preprints.)<br>VP: Accepts first submission only / not applicable<br>L: Authors must use no license (all rights reserved)<br>R: Author choice: No reader registration required, or reader can view basic content (title, abstract) without account but must register to view and download full content	*Journal submission:* Unrestricted - manuscript can be published in any destination<br><br>*Journals directly associated with platform:* JMIR Publications journals<br><br>*Facilitated submission to other journals:* Unknown<br><br>PL: Unknown	EI: Unknown<br><br>MA: Unknown<br><br>MR: No
medRxiv [33] – *Verified*	Multiple article types (including research articles, systematic reviews and meta-analysis, clinical research design protocols, data articles; types not accepted are: theses, opinion pieces, editorials, narrative reviews, datasets)	English	S: PDF, Word doc, WordPerfect<br>V: By PDF download, PDF viewable in browser without download<br>I: DOI (10.1101)<br>VP: Accepts new versions until acceptance at journal / single DOI retained for all versions<br>L: Authors are provided with a choice of licenses: CC0, CC-BY, CC-BY-NC, CC-BY-ND, CC-BY-NC-ND, or no reuse; with no preference for which license chosen<br>R: No reader registration required	*Journal submission:* Unrestricted - manuscript can be published in any destination<br><br>*Journals directly associated with platform:* None<br><br>*Facilitated submission to other journals:* Yes, listed at https://www.medrxiv.org/content/about-medrxiv<br><br>PL: Yes, added by platform within one month of publication	EI: Google Scholar, Microsoft Academic Search, Crossref<br><br>MA: Openly available via Crossref<br><br>MR: No
MitoFit Preprint Archives [34]	Any scholarly content (primary and secondary research)	English	S: PDF (abstract to be submitted as Word doc)<br>V: By PDF download<br>I: DOI (10.26124)<br>VP: Accepts new versions until acceptance at journal / each version receives its own DOI suffix<br>L: Authors must use CC-BY-NC-ND license<br>R: No reader registration required	*Journal submission:* Unrestricted - manuscript can be published in any destination<br><br>*Journals directly associated with platform:* None<br><br>*Facilitated submission to other journals:* Unknown<br><br>PL: Yes, added by platform within one month of publication	EI: Google Scholar<br><br>MA: Openly available via DataCite<br><br>MR: No
NeuroImage: Clinical - *First Look*[b] [35]	Content criteria as directed by associated journal	Unknown	S: PDF, Word doc<br>V: By PDF download, PDF viewable in browser without download<br>I: URL only	*Journal submission:* Manuscripts must be submitted to the NeuroImage: Clinical first, authors are then provided with the option of	EI: Unknown<br><br>MA: Unknown

For peer review only

	(NeuroImage: Clinical)		VP: Unknown / Unknown<br>L: Unknown<br>R: No reader registration required	displaying their manuscript while under review using SSRN's 'First Look'<br><br>PL: Unknown	MR: No
PeerJ Preprints [36] – *Verified*	Any scholarly content (primary and secondary research)	English	S: PDF<br>V: By PDF download, PDF viewable in browser without download, by XML download<br>I: DOI (10.7287)<br>VP: Accepts any new versions until publication in a journal / each version receives its own non-DOI citable identifier<br>L: Authors are provided with a choice of licenses: CC0, CC-BY; with CC-BY preferred<br>R: No reader registration required	*Journal submission:* Unrestricted - manuscript can be published in any destination<br><br>*Journals directly associated with platform:* PeerJ journals<br><br>*Facilitated submission to other journals:* Unknown<br><br>PL: Can be added by author or journal	EI: Google Scholar, Europe PMC<br><br>MA: Openly available via Crossref<br><br>MR: No
Preprints with The Lancet[b] [37] – *Verified*	Content criteria as directed by associated journal(s) (Lancet family of journals)	Unknown	S: Unknown<br>V: By PDF download, PDF viewable in browser without download<br>I: URL only<br>VP: Accepts first submission only / not applicable<br>L: Unknown<br>R: Reader can view basic content (title, abstract) without account but must register to view and download full content	*Journal submission:* Manuscripts must be submitted to the Lancet family of journals (including EBioMedicine and EClinicalMedicine) first, authors are then provided with the option of displaying their manuscript while under review using SSRN's 'First Look'<br><br>PL: Can be added by author or journal	EI: Unknown<br><br>MA: Unknown<br><br>MR: No
Preprints.org [38]	Multiple article types (including research manuscripts, comprehensive reviews, data articles, case reports; types not accepted are: editorials, discussion papers, commentaries, coursework)	English	S: Word doc, LaTeX<br>V: By PDF download<br>I: DOI (10.20944)<br>VP: Accepts any new versions / each version receives its own DOI and own non-DOI citable identifier<br>L: Authors must use CC-BY license<br>R: No reader registration required	*Journal submission:* Unrestricted - manuscript can be published in any destination<br><br>*Journals directly associated with platform:* None<br><br>*Facilitated submission to other journals:* Not available<br><br>PL: Unknown	EI: Google Scholar, PrePubMed, Europe PMC, SHARE, SciLit<br><br>MA: Openly available via Crossref<br><br>MR: No
Research Square [39] – *Verified*	Multiple article types (including research articles, systematic reviews, method articles, data articles; types not accepted are:	English	S: Word doc, LaTeX<br>V: By PDF download, full-text viewable in browser without PDF (e.g. as HTML)<br>I: DOI (10.21203)<br>VP: Accepts any new versions / each version receives its own DOI<br>L: Authors must use CC-BY license	*Journal submission:* Unrestricted - manuscript can be published in any destination<br><br>*Journals directly associated with platform:* Submit directly or submit from BMC journals and more from Springer Nature portfolio listed at https://www.researchsquare.com/journals	EI: Google Scholar, Crossref, Researcher-app<br><br>MA: Openly available via Crossref<br><br>MR: Yes (HTML)

For peer review only

	literature reviews, hypotheses, opinions, theories or commentaries)		R: No reader registration required	*Facilitated submission to other journals:* Not available<br><br>PL: Yes, added by platform within one month of publication	
SciELO Preprints [40] – *Verified*	Research manuscripts only	English, Portuguese, Spanish	S: PDF or any PDF-convertible format<br>V: By PDF download, PDF viewable in browser without download<br>I: URL only<br>VP: Accepts new versions until acceptance at journal / single DOI retained for all versions<br>L: Authors must use CC-BY license<br>R: No reader registration required	*Journal submission:* Unrestricted - manuscript can be published in any destination<br><br>*Journals directly associated with platform:* None (SciELO journals when full operation launched)<br><br>*Facilitated submission to other journals:* Available for any journal using a SWORD (Simple Web-service Offering Repository Deposit) protocol-based manuscript submission platform<br><br>PL: Can be added by author or journal	EI: Google Scholar<br><br>MA: Openly available via API<br><br>MR: No
SSRN [41] – *Verified*	Multiple types (including research manuscripts, abstracts, editorials, opinion papers)	Any language – English preferred, title and abstract must be provided in English, non-English translations can be submitted and linked to English version	S: PDF<br>V: By PDF download, PDF viewable in browser without download<br>I: DOI (10.2139)<br>VP: Accepts any new versions / unknown<br>L: Unknown<br>R: No reader registration required	*Journal submission:* Unrestricted - manuscript can be published in any destination<br><br>*Journals directly associated with platform:* None<br><br>*Facilitated submission to other journals:* Not available<br><br>PL: Unknown	EI: Unknown<br><br>MA: Unknown<br><br>MR: No
Surgery Open Science – *First Look*[b] [42] – *Verified*	Content criteria as directed by associated journal (Surgery Open Science)	Unknown	S: Unknown<br>V: By PDF download, PDF viewable in browser without download<br>I: URL only<br>VP: Unknown<br>L: Unknown<br>R: Reader can view basic content (title, abstract) without account but must register to view and download full content	*Journal submission:* Manuscripts must be submitted to Surgery Open Science first, authors are then provided with the option of displaying their manuscript while under review using SSRN's 'First Look'<br><br>PL: Unknown	EI: Unknown<br><br>MA: Unknown<br><br>MR: No
Therapoid [43] – *Verified*	Research manuscripts only (multimedia content accepted in associated discussion forum)	English – Chinese also to be supported in future	S: PDF<br>V: By PDF download, PDF viewable in browser without download<br>I: URL only<br>VP: Accepts any new versions / each version receives its own DOI<br>L: Authors must use CC-BY license<br>R: No reader registration required	*Journal submission:* Unrestricted – manuscript can be published in any destination<br><br>*Journals directly associated with platform:* None<br><br>*Facilitated submission to other journals:* Not available yet (to be provided for any and only open access journals)	EI: Not indexed<br><br>MA: Openly available via Crossref<br><br>MR: No (plan to require HTML in future)

For peer review only

				PL: Yes, added by platform within one month of publication	
ViXra [44]	Multiple types (including research articles, books, theses)	Any language – English preferred, non-English translations can be provided with English version, as new versions or submitted separately	S: PDF<br>V: By PDF download, PDF viewable in browser without download<br>I: URL, Platform-specific ID<br>VP: Accepts any new versions / each version retains ID with a version number<br>L: Authors can use any license<br>R: No reader registration required	*Journal submission:* Unrestricted - manuscript can be published in any destination<br><br>*Journals directly associated with platform:* None<br><br>*Facilitated submission to other journals:* Not available<br><br>PL: Can be added by author or journal	EI: Not indexed<br><br>MA: Unknown<br><br>MR: No

[a] Where primary research is research based on empirical data and secondary research is derived from primary research, such as literature reviews and meta-reviews; [b] See SSRN for general platform information

For peer review only

Table 3: Screening, moderation, and permanence of content

Preprint Server	Stated not peer reviewed on manuscript record webpage (M) /general server webpages (S)	Advisory Board (A) / Includes Researchers (R)	Commitment to FAIR (Findable, Accessible, Interoperable and Reusable) principles / Data availability statement (DAS)	Screening checks performed between submitting and posting (stating where this process involves active researchers)	Permanence of content (P) / Reasons for retraction (R) / Web presence of removed content (W)	Mechanisms to report concerns about posted content	Sustainability of the service (S) / Preservation of content (P)
**OSF Communities**							
AfricArxiv [1] – *Verified*	M: No<br>S: Yes	A: Yes<br>R: Yes	Yes, endorses, stated on website<br><br>DAS: Not requested or required	'moderated submission check for quality standard, good scientific practices, open science principles', including data/code availability	P: Permanent with some removal options in extraneous circumstances<br>R: Plagiarism, false or inaccurate content<br>W: Basic information remains on a tombstone page	Email administrator; helpdesk support from COS (Centre for Open Science)	S: COS receive external financial support (e.g. grant, individual); operators report no sources of income, staffed on voluntary basis<br>P: COS Preservation Fund to maintain read access for 50+ years
AgriXiv [2] – *Verified*	M: No<br>S: No	A: Yes<br>R: Yes	Yes<br><br>DAS: Unknown	No information	P: Permanent with some removal options in extraneous circumstances<br>R: Plagiarism, false or inaccurate content<br>W: Basic information remains on a tombstone page	Email administrator; helpdesk support from COS	S: COS receive external financial support (e.g. grant, individual); operators report Open Access India plans to raise funds<br>P: COS Preservation Fund to maintain read access for 50+ years
Arabixiv [3] – *Verified*	M: No<br>S: No	A: Yes<br>A: Unknown	Yes<br><br>DAS: Unknown	Arabixiv editors check for: ensuring it is academic research	P: Permanent with some removal options in extraneous circumstances<br>R: Plagiarism, false or inaccurate content<br>W: Basic information remains on a tombstone page	Email administrator; helpdesk support from COS; Readers encouraged to post concerns publicly as new article	S: COS receive external financial support (e.g. grant, individual); no further information to report from operators<br>P: COS Preservation Fund to maintain read access for 50+ years
EcoEvoRxiv [4] – *Verified*	M: No<br>S: Yes	A: Yes<br>R: Yes	Yes<br><br>DAS: Unknown	Steering committee member (*including active researchers*) checks for: competing interests, plagiarism, misconduct or integrity checks, ethical and legal compliance, funder acknowledgment	P: Permanent with some removal options in extraneous circumstances<br>R: Plagiarism, false or inaccurate content<br>W: Basic information remains on a tombstone page	Email administrator; helpdesk support from COS	S: COS receive external financial support (e.g. grant, individual); no further information to report from operators<br>P: COS Preservation Fund to maintain read access for 50+ years

For peer review only

FocUS Archive [5] – *Verified*	M: No<br>S: No	A: Yes<br>R: Yes	Yes<br><br>DAS: Unknown	There is a moderation process, no further information known	P: Permanent with some removal options in extraneous circumstances<br>R: Plagiarism, false or inaccurate content<br>W: Basic information remains on a tombstone page	Email administrator; helpdesk support from COS	S: COS receive external financial support (e.g. grant, individual); nothing to report from operators<br>P: COS Preservation Fund to maintain read access for 50+ years
Frenxiv [6] – *Verified*	M: No<br>S: No	A: Yes<br>R: Yes	Yes<br><br>DAS: Unknown	Editorial-advisory members (*including active researchers*) check for: competing interests, plagiarism, misconduct or integrity checks, authors are genuine, ethical and legal compliance, data availability	P: Permanent with some removal options in extraneous circumstances<br>R: Plagiarism, false or inaccurate content<br>W: Basic information remains on a tombstone page	Email administrator; helpdesk support from COS	S: COS receive external financial support (e.g. grant, individual); nothing to report from operators<br>P: COS Preservation Fund to maintain read access for 50+ years
INA-Rxiv [7] – *Verified*	M: No<br>S: Yes	A: Yes<br>R: Yes	Yes<br><br>DAS: Unknown	Unknown	P: Permanent with some removal options in extraneous circumstances<br>R: Plagiarism, false or inaccurate content<br>W: Basic information remains on a tombstone page	Email administrator; helpdesk support from COS	S: COS receive external financial support (e.g. grant, individual); operators endorse users to donate to COS<br>P: COS Preservation Fund to maintain read access for 50+ years
MarXiv [8] – *note: no longer on the OSF – Verified*	M: No<br>S: Yes	A: Yes<br>R: Yes	Yes<br><br>DAS: Unknown	The MarXiv team check for: plagiarism, misconduct or integrity checks, ethical and legal compliance	P: Permanent with some removal options in extraneous circumstances<br>R: Plagiarism, false or inaccurate content<br>W: Basic information remains on a tombstone page	Email administrator; helpdesk support from COS	S: COS receive external financial support (e.g. grant, individual); service moving to another host<br>P: COS Preservation Fund to maintain read access for 50+ years
MetaArXiv [9] – *Verified*	M: No<br>S: No	A: Yes<br>R: Yes	Yes<br><br>DAS: Not requested or required	BITSS staff check for: relevance of material, author name matches submitter	P: Permanent with some removal options in extraneous circumstances<br>R: Plagiarism, false or inaccurate content<br>W: Basic information remains on a tombstone page	Email administrator; helpdesk support from COS; Community moderation via commenting	S: COS receive external financial support (e.g. grant, individual); operation supported by BITSS funders and revenue<br>P: COS Preservation Fund to maintain read access for 50+ years
MindRxiv [10] – *Verified*	M: No<br>S: Yes	A: Yes<br>R: Yes	Yes<br><br>DAS: Not requested or required	Science Director at Mind & Life (*including active researchers*) checks for: relevance of material, ensuring it is academic research	P: Permanent with some removal options in extraneous circumstances<br>R: Plagiarism, false or inaccurate content<br>W: Basic information remains on a tombstone page	Email administrator; helpdesk support from COS	S: COS receive external financial support (e.g. grant, individual); nothing to report from operators<br>P: COS Preservation Fund to maintain read access for 50+ years

For peer review only

NutriXiv [11] – *Verified*	M: No<br>S: No	A: Unknown<br>R: Unknown	Yes<br><br>DAS: Unknown	No information	P: Permanent with some removal options in extraneous circumstances<br>R: Plagiarism, false or inaccurate content<br>W: Basic information remains on a tombstone page	Email administrator; helpdesk support from COS	S: COS receive external financial support (e.g. grant, individual); nothing to report from operators<br>P: COS Preservation Fund to maintain read access for 50+ years
OSF Preprints [12] – *Verified*	M: No<br>S: Yes	A: Yes<br>R: Yes	Yes<br><br>DAS: Not requested or required	OSF staff members run automated checks for spam	P: Permanent with some removal options in extraneous circumstances<br>R: Plagiarism, false or inaccurate content<br>W: Basic information remains on a tombstone page	Email administrator; helpdesk support from COS	S: COS receive external financial support (e.g. grant, individual)<br>P: COS Preservation Fund to maintain read access for 50+ years
PaleorXiv [13] – *Verified*	M: No<br>S: Yes	A: Yes<br>R: Yes	Yes<br><br>DAS: Not requested or required	A moderator from the PaleoRxiv steering committee (*including active researchers*) checks for: plagiarism, relevance of material, ensuring it is academic research	P: Permanent with some removal options in extraneous circumstances<br>R: Plagiarism, false or inaccurate content<br>W: Basic information remains on a tombstone page	Email administrator; helpdesk support from COS	S: COS receive external financial support (e.g. grant, individual); nothing to report from operators<br>P: COS Preservation Fund to maintain read access for 50+ years
PsyArXiv [14] – *Verified*	M: No<br>S: Yes	A: Yes<br>R: Yes	Yes<br><br>DAS: Not requested or required	Moderation team checks for: ethical and legal compliance, relevance of material, copyright violations, spam	P: Permanent with some removal options in extraneous circumstances<br>R: Plagiarism, false or inaccurate content<br>W: Basic information remains on a tombstone page	Email administrator; helpdesk support from COS	S: COS receive external financial support (e.g. grant, individual); nothing to report from operators<br>P: COS Preservation Fund to maintain read access for 50+ years
SocArxiv [15] – *Verified*	M: No<br>S: Yes	A: Yes<br>R: Yes	Yes<br><br>DAS: Unknown	There is a moderation process, no further information known	P: Permanent with some removal options in extraneous circumstances<br>R: Plagiarism, false or inaccurate content<br>W: Basic information remains on a tombstone page	Email administrator; helpdesk support from COS	S: COS receive external financial support (e.g. grant, individual); nothing to report from operators<br>P: COS Preservation Fund to maintain read access for 50+ years
SportRxiv [16] – *Verified*	M: No<br>S: Yes	A: Yes<br>R: Yes	Yes<br><br>DAS: Not requested or required	SportRxiv editors (*including active researchers*) check for: basic scientific standards, authors are genuine, all authors provide permission to post	P: Permanent with some removal options in extraneous circumstances<br>R: W: Basic information remains on a tombstone page	Email administrator; helpdesk support from COS	S: COS receive external financial support (e.g. grant, individual); operators report membership fees help offset costs<br>P: COS Preservation Fund to maintain read access for 50+ years
Thesis Commons [17] – *Verified*	M: No<br>S: No	A: Yes<br>R: Yes	Yes	There is no moderation process beyond that for OSF Preprints (OSF staff check for spam)	P: Permanent with some removal options in extraneous circumstances	Email administrator; helpdesk support from COS	S: COS receive external financial support (e.g. grant,

For peer review only

			DAS: Not requested or required		R: Plagiarism, false or inaccurate content<br>W: Basic information remains on a tombstone page		individual); nothing to report from operators<br>P: COS Preservation Fund to maintain read access for 50+ years
**Open Research Central infrastructure**							
AAS Open Research [18] – *Verified*	M: Yes<br>S: Yes	A: Yes<br>R: Yes	Yes, compliant, stated on website<br><br>DAS: Required	F1000's in-house editorial team check for: competing interest declaration, plagiarism, authors are genuine and at least one eligible for platform, authors provide permission to post, ethical and legal compliance, funder acknowledgment, clinical trial registration, data/code availability, minimum reporting standards	P: Permanent with some removal options in extraneous circumstances<br>R: Contravention of copyright, plagiarism, false or inaccurate content, ethical and legal issues<br>W: Basic information remains on a tombstone page	Community moderation via commenting	S: Article processing charges, covered by funding agency for AAS, Gates, HRB, MNI and Wellcome (MNI - limited to one article per lab per year)<br>P: Preprints permanently archived in Portico
AMRC Open Research [19] – *Verified*	M: Yes<br>S: Yes	A: Not yet<br>R: Still finalising advisory board					
Gates Open Research [20] – *Verified*	M: Yes<br>S: Yes	A: Yes<br>R: Yes					
HRB Open Research [21] – *Verified*	M: Yes<br>S: Yes	A: Yes<br>R: Yes					
MNI Open Research [22] – *Verified*	M: Yes<br>S: Yes	A: Not yet<br>R: Not applicable					
Wellcome Open Research [23] – *Verified*	M: Yes<br>S: Yes	A: Yes<br>R: Yes					
**Others**							
arXiv [24] – *Verified*	M: No<br>S: Yes	A: Yes<br>R: Yes	None as yet<br><br>DAS: Not requested or required	arXiv moderators (*including active researchers*) check for: plagiarism, misconduct or integrity checks, authors are genuine, ethical and legal compliance, relevance of material	P: Permanent with no removal options (as long as archive exists)<br>R: None<br>W: Not applicable	Social media or direct with authors (except copyright violations or significant issues)	S: External financial support (grants and membership program: libraries, research labs, philanthropy, government funding)<br>P: Persistent access through mirror sites, no external preservation services used as yet
Authorea [25] – *Verified*	M: No<br>S: Yes	A: No<br>R: Not applicable (no advisory board)	Yes, in process of becoming compliant, without stating on website<br><br>DAS: Not requested or required	Submissions are checked for spam	P: Content maybe removed by operator/owner only<br>R: Policy not yet in place<br>W: Not applicable (at this time)	Email administrator	S: Other business model not based on direct preprinting charges or associated journal (acquisition and investment from Atypon, part of Wiley)<br>P: Preprints permanently archived in Portico

For peer review only

bioRxiv [26] – *Verified*	M: Yes<br>S: Yes	A: Yes<br>R: Yes	None as yet<br><br>DAS: Not requested or required	bioRxiv staff and scientific affiliates (*including active researchers*) check for: plagiarism, misconduct or integrity checks, ethical and legal compliance, clinical trial registration, submission is within scope and no danger to human health	P: Permanent with withdrawal options and (rare) removal in extraneous circumstances<br>R: Removal: Legal or biohazard reasons; Withdrawal: Plagiarism, false or inaccurate content, ethical and legal issues, co-author(s) did not consent to posting<br>W: Withdrawn articles remain visible; For removed articles, basic information remains on a tombstone page	Email administrator	S: Internal (Cold Spring Harbor Laboratory) and external financial support (multiyear grant funding from Chan Zuckerberg Initiative)<br>P: Preprints permanently archived in Portico
Cell Press Sneak Peek [27] – *Verified*	M: Yes<br>S: Yes	A: Yes<br>R: Yes	Unknown	No information	P: Unknown<br>R: Unknown<br>W: Unknown	Unknown	S: Unknown<br>P: Unknown
ChemRxiv [28]	M: Yes<br>S: Yes	A: Yes<br>R: Yes	Yes, compliant, without stating on website<br><br>DAS: Not requested or required	PhD-level chemists (*including active researchers*) check for: competing interests declared, plagiarism, misconduct or integrity checks, authors are genuine, all authors provide permission to post, ethical and legal compliance, funder acknowledgment, external data/code availability	P: Permanent with some removal options in extraneous circumstances<br>R: Contravention of copyright, plagiarism, ethical and legal issues<br>W: Basic information remains on a tombstone page	Email administrator, or contact ethics departments of governing partners	S: Nothing to report from server<br>P: Agreement to dark archive content with TIB (Leibniz Information Centre for Science and Technology University Library)
ChinaXiv [29]	M: No<br>S: No	A: Yes<br>R: Yes	Unknown<br><br>DAS: Not requested or required	Project team and scientists (*including active researchers*) check for: plagiarism, misconduct or integrity checks, authors are genuine	P: Permanent with no removal options<br>R: Contravention of copyright, plagiarism, false or inaccurate content, ethical and legal issues<br>W: Basic information remains on a tombstone page	None	S: Nothing to report from server<br>P: Long term preservation in local
ESSOAr [30] – *Verified*	M: Yes<br>S: Yes	A: Yes<br>R: Yes	Yes, without stating on website<br><br>DAS: Not requested or required	Editorial board members (*including active researchers*) check for: plagiarism, misconduct or integrity checks, authors are genuine, all authors provide permission to post, submission is scholarly and within scope	P: Permanent with some removal options in extraneous circumstances<br>R: Copyright infringement or other ethical or legal issues the Advisory Board deems critical	Email administrator	S: Internal financial support (investment from Wiley and AGU)<br>P: On roadmap: preprints to be permanently archived in Portico

For peer review only

					W: Unknown		
F1000 Research [31] – *Verified*	M: Yes<br>S: Yes	A: Yes<br>R: Yes	Yes, compliant, stated on website<br><br>DAS: Required	F1000's in-house editorial team check for: competing interest declaration, plagiarism, authors are genuine and at least one eligible for platform, authors provide permission to post, ethical and legal compliance, funder acknowledgment, clinical trial registration, data/code availability, minimum reporting standards, relevance of material	P: Permanent with some removal options in extraneous circumstances<br>R: Contravention of copyright, false or inaccurate content, ethical and legal issues<br>W: Basic information remains on a tombstone page	Email administrator	S: Nothing to report from server<br>P: Preprints permanently archived in Portico
JMIR Preprints [32]	M: No<br>S: Yes	A: Unknown<br>R: Unknown	Unknown	Unknown	P: Unknown<br>R: Unknown<br>W: Unknown	Unknown	S: Unknown<br>P: Unknown
medRxiv [33] – *Verified*	M: Yes<br>S: Yes	A: In formation (January 2020)<br>R: Yes	None as yet<br><br>DAS: Required	Submission is checked for: competing interests declared, plagiarism, misconduct or integrity checks, minimal reporting standards, ethical and legal compliance, clinical trial registration, funder acknowledgment, submission is within scope and no danger to human health	P: Permanent with withdrawal options and (rare) removal in extraneous circumstances<br>R: Removal: Legal or biohazard reasons; Withdrawal: Plagiarism, false or inaccurate content, ethical and legal issues, co-author(s) did not consent to posting<br>W: Withdrawn articles remain visible; For removed articles, basic information remains on a tombstone page	Unknown	S: Supported by CSHL<br>P: Preprints permanently archived in Portico
MitoFit Preprint Archives [34]	M: Yes<br>S: Yes	A: Yes<br>R: Yes	Yes, endorses, stated on website<br><br>DAS: Encouraged	Scientific Advisory Board members (*including active researchers*) check for: competing interests declared, misconduct or integrity checks, authors are genuine, funder acknowledgment	P: Permanent with some removal options in extraneous circumstances<br>R: Plagiarism, ethical and legal issues, no appropriate declaration of conflicts of interest<br>W: Basic information remains on a tombstone page	Community moderation via commenting	S: Other business model not based on direct preprinting charges or associated journal (maintained by MitoFit DOI Data Center)<br>P: Backup kept in secondary server
NeuroImage: Clinical - *First Look* [35] – *Verified*	M: Yes<br>S: Yes	A: Unknown<br>R: Unknown	Unknown	No information	P: Unknown<br>R: Unknown<br>W: Unknown	Unknown	S: Unknown<br>P: Unknown

For peer review only

PeerJ Preprints [36] – *Verified*	M: Yes<br>S: Yes	A: Yes<br>R: Yes	None<br><br>DAS: Encouraged	PeerJ staff check for: competing interests declared, misconduct or integrity checks, ethical and legal compliance, funder acknowledgment	P: Permanent with some removal options in extraneous circumstances<br>R: False or inaccurate content, additional reasons not stated<br>W: Basic information remains on a tombstone page	Email administrator	S: Supported by associated journal publishing (PeerJ)<br>P: Preprints permanently archived in Portico and EuropePMC
Preprints with The Lancet [37] – *Verified*	M: Yes<br>S: Yes	A: Unknown<br>R: Unknown	Unknown	SSRN staff and a Lancet editor check for: competing interests declared, ethical and legal compliance, funder acknowledgment, clinical trial registration, relevance of material	P: Unknown<br>R: Unknown<br>W: Unknown	Unknown	S: Unknown<br>P: Unknown
Preprints.org [38]	M: Yes<br>S: Yes	A: Yes<br>R: Yes	None as yet<br><br>DAS: Encouraged	Preprints staff and advisory board members (*including active researchers*) check for: plagiarism (as needed), authors are genuine, ethical and legal compliance, basic scientific content, data/code availability	P: Permanent with some removal options in extraneous circumstances<br>R: Contravention of copyright, plagiarism, false or inaccurate content, ethical and legal issues, misconduct<br>W: Basic information remains on a tombstone page if DOI has been issued, otherwise no web presence retained	Email administrator	S: Supported by associated journal publishing (MDPI)<br>P: Currently no long-term preservation strategy
Research Square [39] – *Verified*	M: Yes<br>S: Yes	A: Not yet<br>R: Not applicable	None as yet<br><br>DAS: Not requested or required	In-house editorial team checks for: competing interests declared, plagiarism (only for submissions to BMC journals), ethical and legal compliance, funder acknowledgment, clinical trial registration, relevance of material, basic scientific content, language, potential for harm	P: Permanent with some removal options in extraneous circumstances<br>R: Contravention of copyright, ethical and legal issues<br>W: Basic information remains on a tombstone page	Email administrator	S: Other business model not based on direct preprinting charges or associated journal publishing (publishing services by Research Square)<br>P: Preprints permanently archived in Portico
SciELO Preprints [40] – *Verified*	M: No<br>S: No	A: Yes<br>R: Yes	Yes, endorses, without stating on website<br><br>DAS: Not requested or required	SciELO editors (*including active researchers*) will check for: competing interests declared, plagiarism, misconduct and integrity checks, ethical and legal compliance, authors are genuine, all authors provide permission to post, clinical trial registration, funder acknowledgment, data/code availability	P: Permanent with some removal options in extraneous circumstances<br>R: Contravention of copyright, plagiarism, false or inaccurate content, ethical and legal issues<br>W: No web presence retained	Email administrator	S: Internal financial support (SciELO program)<br>P: Plan to use LOCKSS-compatible preservation strategy [once full operation launched]

For peer review only

SSRN [41] – *Verified*	M: No<br>S: No	A: Yes<br>R: Yes	Unknown	SSRN staff check for relevance of material	P: Content maybe removed by author (or operator/owner)<br>R: Contravention of copyright, plagiarism, false or inaccurate content, ethical and legal issues<br>W: Unknown	Email administrator	S: Unknown<br>P: Unknown
Surgery Open Science - *First Look* [42] – *Verified*	M: No<br>S: No	A: Unknown<br>R: Unknown	Unknown	Unknown	P: Unknown<br>R: Unknown<br>W: Unknown	Unknown	S: Unknown<br>P: Unknown
Therapoid [43] – *Verified*	M: No<br>S: No	A: Yes<br>R: Yes	None as yet<br><br>DAS: Not requested or required	There is no pre-moderation of submissions; any checks are performed by site users (*including active researchers*) after posting	P: Permanent with some removal options in extraneous circumstances<br>R: Contravention of copyright, plagiarism, false or inaccurate content, ethical and legal issues<br>W: No web presence retained	Not yet in place: expect community moderation via forum	S: Unknown<br>P: All content is preserved – no further information reported by server
ViXra [44]	M: Yes<br>S: Yes	A: Yes<br>R: Yes	Mostly compliant, without endorsing, without stating on website<br><br>DAS: Not requested or required	Administrators do not perform formal screening checks but reject submissions in response to issues relating to: plagiarism, misconduct and integrity checks, ethical and legal compliance, format (PDF)	P: Content maybe removed by author (or operator/owner)<br>R: Contravention of copyright, plagiarism, false or inaccurate content, ethical and legal issues<br>W: No web presence retained	Email administrator	S: Sustainable low-cost web hosting service secured, previously run on donations<br>P: Long-term archiving provided by mirror site and cloud storage of database

For peer review only

Table 4: Usage metrics and other features

Preprint Server	Usage metrics on abstract page						Other features			
**OSF Communities**	Number of views	Number of downloads	Number of citations (e.g. Google Scholar)	Number of comments	Altmetrics	Social media interactions (from platform not Altmetric)	Commenting (including annotation plug-ins)	Onsite search	Alerts (e.g. RSS (Really Simple Syndication) feeds, email alerts)	Other
AfricArxiv [1] – *Verified*		⊗					⊗	⊗		
AgriXiv [2] – *Verified*		⊗					⊗	⊗		
Arabixiv [3] – *Verified*		⊗					⊗	⊗		
EcoEvoRxiv [4] – *Verified*		⊗						⊗		
FocUS Archive [5] – *Verified*		⊗						⊗		
Frenxiv [6]] – *Verified*		⊗					⊗	⊗		
INA-Rxiv [7]] – *Verified*		⊗					⊗	⊗		
MarXiv [8] – *note: no longer on the OSF – Verified*		⊗					⊗	⊗		
MetaArXiv [9] – *Verified*		⊗					⊗	⊗		
MindRxiv [10] – *Verified*		⊗					⊗	⊗		
NutriXiv [11] – *Verified*		⊗					⊗	⊗		
OSF Preprints [12] – *Verified*		⊗					⊗	⊗		
PaleorXiv [13] – *Verified*		⊗					⊗	⊗		
PsyArXiv [14] – *Verified*		⊗					⊗	⊗		
SocArxiv [15] – *Verified*		⊗					⊗	⊗	⊗	
SportRxiv [16] – *Verified*		⊗					⊗	⊗		
Thesis Commons [17] – *Verified*		⊗					⊗	⊗		
**Open Research Central infrastructure**										

For peer review only

AAS Open Research [18] – *Verified*	⊗	⊗	⊗		⊗		⊗	⊗		'Link to Google Scholar citations, Blog and gateways'
AMRC Open Research [19] – *Verified*	⊗	⊗	⊗		⊗		⊗	⊗		'Link to Google Scholar citations'
Gates Open Research [20] – *Verified*	⊗	⊗	⊗		⊗		⊗	⊗		'Link to Google Scholar citations, Gateways'
HRB Open Research [21] – *Verified*	⊗	⊗	⊗		⊗		⊗	⊗		'Link to Google Scholar citations, Blog'
MNI Open Research [22] – *Verified*	⊗	⊗	⊗		⊗		⊗	⊗		'Link to Google Scholar citations'
Wellcome Open Research [23] – *Verified*	⊗	⊗	⊗		⊗		⊗	⊗		'Link to Google Scholar citations, Blog and gateways'
**Others**										
arXiv [24] – *Verified*								⊗	⊗	
Authorea [25] – *Verified*	⊗	⊗				⊗	⊗			'Forking (copying an article that may or may not be authored by you), follow the article'
bioRxiv [26] – *Verified*	⊗	⊗		⊗	⊗	⊗	⊗	⊗	⊗	'Channels, PREreview reviews linked, PreLights blog linked, Pingbacks to blog, PCI, biOverlay and Twitter thread embedded'
Cell Press Sneak Peek [27] – *Verified*	⊗	⊗								Unknown 'other'
ChemRxiv [28]	⊗	⊗	⊗		⊗					Unknown 'other'
ChinaXiv [29]	⊗	⊗					⊗		⊗	'News, Author blacklist'
ESSOAr [30] – *Verified*		⊗			⊗			⊗	⊗	'Plaudit, QR code, bookmarks, reference management tools'
F1000 Research [31] – *Verified*	⊗	⊗	⊗		⊗		⊗	⊗	⊗	'Link to Google Scholar citations'
JMIR Preprints [32]						⊗	⊗	⊗		
medRxiv [33] – *Verified*	⊗	⊗		⊗	⊗	⊗	⊗	⊗	⊗	
MitoFit Preprint Archives [34]										None

NeuroImage: Clinical - *First Look* [35] – *Verified*	⊗	⊗								Unknown 'other'
PeerJ Preprints [36] – *Verified*	⊗	⊗		⊗		⊗	⊗	⊗	⊗	
Preprints with The Lancet [37] – *Verified*	⊗	⊗								Unknown 'other'
Preprints.org [38]	⊗	⊗		⊗	⊗		⊗	⊗	⊗	Embedded bookmark tabs for reading and reference management tools
Research Square [39] – *Verified*	⊗	⊗		⊗	⊗		⊗	⊗		Email alerts with comments
SciELO Preprints [40] – *Verified*	⊗	⊗			⊗	⊗ (when regular operation launches in 2020)	⊗ (when regular operation launches in 2020)	⊗ (when regular operation launches in 2020)		
SSRN [41] – *Verified*	⊗	⊗								Plum metrics
Surgery Open Science - *First Look* [42] – *Verified*	⊗	⊗						⊗	⊗ (SSRN subscription)	
Therapoid [43] – *Verified*	'metrics coming soon'						⊗	⊗	⊗	'Blockchain technology being implemented'
ViXra [44]	⊗						⊗		⊗	

Table 5: Metadata

Preprint Server	Title	DOI or identifier	Publication or deposition date	Author name(s)	Author affiliation(s)	Funder acknowledge-ment(s)	Subject category	License	Abstract	Full-text content	References	Relational link to journal publication version (where it exists)	Other
**OSF Communities**													
AfricArxiv [1] – *Verified*	⊗	⊗	⊗	⊗	⊗		⊗	⊗	⊗			⊗	
AgriXiv [2] – *Verified*	⊗	⊗	⊗	⊗			⊗	⊗	⊗			⊗	
Arabixiv [3] – *Verified*	⊗	⊗	⊗	⊗			⊗	⊗	⊗			⊗	
EcoEvoRxiv [4] – *Verified*	⊗	⊗	⊗	⊗			⊗	⊗	⊗				
FocUS Archive [5] – *Verified*	⊗	⊗	⊗	⊗			⊗	⊗	⊗			⊗	
Frenxiv [6] – *Verified*	⊗	⊗	⊗	⊗			⊗	⊗	⊗			⊗	
INA-Rxiv [7] – *Verified*	⊗	⊗	⊗	⊗			⊗	⊗	⊗			⊗	
MarXiv [8] – *Verified*	⊗	⊗	⊗	⊗			⊗	⊗	⊗			⊗	
MetaArXiv [9]– *Verified*	⊗	⊗	⊗	⊗			⊗	⊗	⊗			⊗	
MindRxiv [10] – *Verified*	⊗	⊗	⊗	⊗			⊗	⊗	⊗			⊗	
NutriXiv [11] – *Verified*	⊗	⊗	⊗	⊗					⊗				
OSF Preprints [12] – *Verified*	⊗	⊗	⊗	⊗			⊗	⊗	⊗			⊗	
PaleorXiv [13] – *Verified*	⊗	⊗	⊗	⊗			⊗	⊗	⊗			⊗	
PsyArXiv [14] – *Verified*	⊗	⊗	⊗	⊗			⊗	⊗	⊗			⊗	
SocArxiv [15] – *Verified*	⊗	⊗	⊗	⊗			⊗	⊗	⊗			⊗	
SportRxiv [16] – *Verified*	⊗	⊗	⊗	⊗			⊗	⊗	⊗			⊗	
Thesis Commons [17] – *Verified*	⊗	⊗	⊗	⊗			⊗	⊗	⊗			⊗	
**Open Research Central infrastructure**													

For peer review only

AAS Open Research [18] – *Verified*	⊗	⊗	⊗	⊗	⊗	⊗	⊗		⊗	⊗			Competing interests, Referee (name, affiliation, report), Commenter (name, affiliation, comment text), Study type, Article type
AMRC Open Research [19] – *Verified*													
Gates Open Research [20] – *Verified*													
HRB Open Research [21] – *Verified*													
MNI Open Research [22] – *Verified*													
Wellcome Open Research [23] – *Verified*													
**Others**													
arXiv [24] – *Verified*	⊗	⊗	⊗	⊗					⊗			⊗	
Authorea [25] – *Verified*	⊗	⊗	⊗	⊗	⊗		⊗		⊗	⊗			Keywords
bioRxiv [26] – *Verified*	⊗	⊗	⊗	⊗	⊗		⊗	⊗	⊗	⊗	⊗	⊗	
Cell Press Sneak Peek[b] [27] – *Verified*													No information
ChemRxiv [28]	⊗	⊗	⊗	⊗	⊗	⊗	⊗	⊗	⊗	⊗	⊗		
ChinaXiv [29]	⊗	⊗	⊗	⊗			⊗		⊗				
ESSOAr [30] – *Verified*	⊗	⊗	⊗	⊗	⊗	⊗	⊗	⊗	⊗			⊗	ORCID, keywords
F1000 Research [31] – *Verified*	⊗	⊗	⊗	⊗	⊗	⊗	⊗		⊗	⊗			Competing interests, Article is a Faculty Review (yes/no), Referee (name, affiliation, report), Commenter (name, affiliation,

For peer review only

													comment text), Study type, Article type
JMIR Preprints [32]	⊗	⊗	⊗	⊗				⊗	⊗				Journal currently submitted to
medRxiv [33] – *Verified*	⊗	⊗	⊗	⊗	⊗	⊗	⊗	⊗	⊗		⊗	⊗	
MitoFit Preprint Archives [34]	⊗	⊗	⊗	⊗	⊗	⊗	⊗	⊗	⊗				ORCID if provided, Language, Version
NeuroImage: Clinical - *First Look*[b] [35] – *Verified*													No information
PeerJ Preprints [36] – *Verified*	⊗	⊗	⊗	⊗	⊗	⊗	⊗	⊗	⊗			⊗	
Preprints with The Lancet[b] [37] – *Verified*													No information
Preprints.org [38]	⊗	⊗	⊗	⊗				⊗	⊗			⊗	
Research Square [39] – *Verified*	⊗	⊗	⊗	⊗	⊗	⊗	⊗	⊗	⊗	⊗	⊗	⊗	
SciELO Preprints [40] – *Verified*	⊗		⊗	⊗	⊗		⊗	⊗	⊗				Language
SSRN [41] – *Verified*	⊗	⊗	⊗	⊗					⊗		⊗		
Surgery Open Science – *First Look*[b] [42] – *Verified*													No information
Therapoid [43] – *Verified*	⊗	⊗	⊗	⊗			⊗		⊗				"Anything else the author provides"
ViXra [44]	⊗	⊗	⊗	⊗					⊗				

For peer review only

Preprint platform websites

1. AfricaArxiv https://info.africarxiv.org/
2. AgriXiv https://agrixiv.org
3. Arabixiv https://arabixiv.org/
4. EcoEvoRxiv https://ecoevorxiv.org
5. FocUS Archive https://osf.io/preprints/focusarchive/
6. Frenxiv https://frenxiv.org
7. INA-Rxiv https://osf.io/preprints/inarxiv
8. MarXiv https://marxiv.org
9. MetaArXiv https://osf.io/preprints/metaarxiv/
10. MindRxiv https://mindrxiv.org
11. NutriXiv https://osf.io/preprints/nutrixiv
12. OSF Preprints https://osf.io/preprints/
13. PaleorXiv https://paleorxiv.org
14. PsyArXiv https://psyarxiv.com
15. SocArXiv https://osf.io/preprints/socarxiv
16. SportRxiv https://osf.io/preprints/sportrxiv
17. Thesis Commons https://thesiscommons.org
18. AAS Open Research https://aasopenresearch.org/
19. AMRC Open Research https://amrcopenresearch.org/
20. Gates Open Research https://gatesopenresearch.org/
21. HRB Open Research https://hrbopenresearch.org/
22. MNI Open Research https://mniopenresearch.org/
23. Wellcome Open Research https://wellcomeopenresearch.org/
24. arXiv https://arxiv.org
25. Authorea https://www.authorea.com
26. bioRxiv https://www.biorxiv.org/
27. Cell Press Sneak Peek https://papers.ssrn.com/sol3/Jeljour_results.cfm?form_name=journalBrowse&journal_id=3184889
28. ChemRxiv https://chemrxiv.org
29. ChinaXiv http://chinaxiv.org
30. ESSOAr https://www.essoar.org
31. F1000 Research https://www.essoar.org
32. JMIR Preprints https://preprints.jmir.org/
33. medRxiv https://www.medrxiv.org
34. MitoFit Preprint Archives https://www.mitofit.org/index.php/MitoFit_Preprint_Archives
35. NeuroImage: Clinical – *First Look*
    https://papers.ssrn.com/sol3/JELJOUR_Results.cfm?form_name=journalBrowse&journal_id=3178959
36. PeerJ Preprints https://peerj.com/preprints/
37. Preprints with The Lancet https://papers.ssrn.com/sol3/JELJOUR_Results.cfm?form_name=journalBrowse&journal_id=3184962
38. Preprints.org https://www.preprints.org/
39. Research Square https://www.researchsquare.com
40. SciELO Preprints https://preprints.scielo.org/index.php/scielo
41. SSRN https://www.ssrn.com
42. Surgery Open Science – *First Look*
    https://papers.ssrn.com/sol3/Jeljour_results.cfm?form_name=journalBrowse&journal_id=3303309
43. Therapoid https://therapoid.net
44. ViXra http://vixra.org

For peer review only

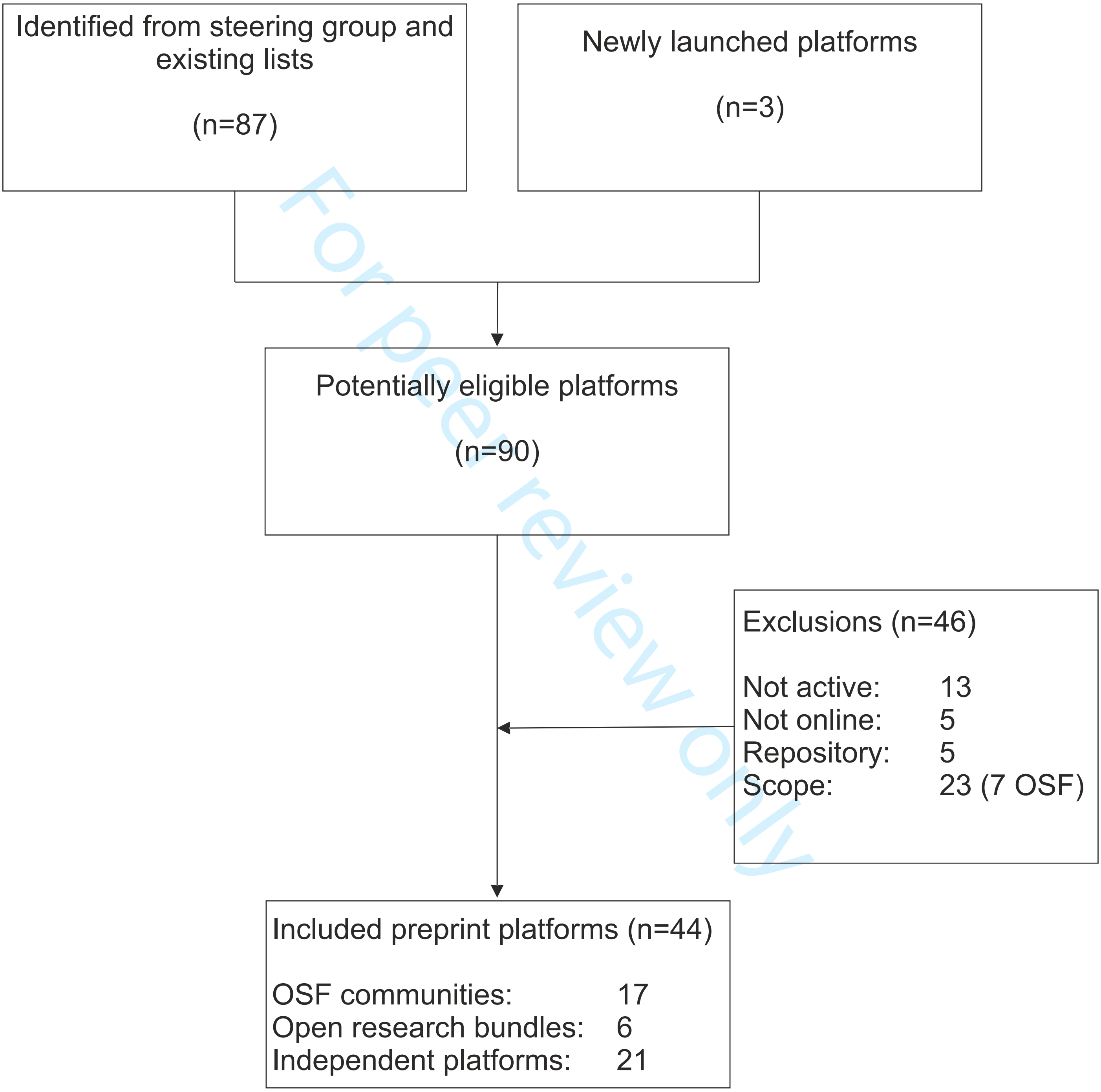
Identified from steering group and existing lists
(n=87)
Newly launched platforms
(n=3)
Potentially eligible platforms
(n=90)
Exclusions (n=46)
Not active: 13
Not online: 5
Repository: 5
Scope: 23 (7 OSF)
Included preprint platforms (n=44)
OSF communities: 17
Open research bundles: 6
Independent platforms: 21

Supplementary Table 1: Reasons for excluded preprint platforms

Name of Site	Reason for Exclusion
Chemweb	Inactive
Centre for Health Economics and Policy Analysis (CHEPA)	Inactive
ClinMed NetPrints	Inactive
Cogprints	Inactive
CSTC	Inactive
K-Theory Preprint Archives	Inactive
Mathematics Preprint Search System (MPRESS)	Inactive
National Advisory Committee for Aeronautics (NACA)	Inactive
Nature Precedings	Inactive
The Winnower	Inactive
Geometry Center's Preprints	Inactive
World Health Organisation Zika Open repository	Inactive
Instant Math Preprints (Yale Mathematics Preprint Bulletin Board)	Inactive
Organisation Européenne pour la Recherche Nucléaire document platform	Repository
Hyper Articles en Ligne (HAL)	Repository
ResearchGate	Repository
White Rose Consortium e-Prints Repository	Repository
Zenodo	Repository
Organisation Européenne pour la Recherche Nucléaire Print	Offline
Fermilab	Offline
Physics Information Exchange (PIE)	Offline
Radio Astronomy Preprints – RAPsheet	Offline
Space Telescope Preprints – STEPsheet	Offline
Advance: a SAGE preprints community	Scope: Humanities and Social Sciences
BodoArXiv	Scope: Medieval Studies
Cryptology ePrint Archive	Scope: Cryptology
CORE repository	Scope: Humanities
EarthArXiv	Scope: Earth Sciences
EconStor	Scope: Economics and Business Studies
ECSarXiv	Scope: Electrochemistry and solid state science
engrXiv	Scope: Engineering
E-LIS	Scope: Library and Information Science
Electronic Colloquium on Computational Complexity	Scope: Computer Science
Institute for Fiscal Studies (IFS) Working Papers	Scope: Economics
LawArxiv	Scope: Law
LIS Scholarship Archive	Scope: Library and Information Science
LingBuzz	Scope: Linguistics
MediArXiv	Scope: Media, Film and Communication Studies
Mathematical Physics Preprint Archive (mp_arc)	Scope: Mathematical Physics and Related Areas
National Bureau of Economic Research (NBER) Working Papers	Scope: Economics
Networked Computer Science Technical Reference Library (NCSTRL)	Scope: Computer Science
Philsci Archive	Scope: Philosophy of Science
Munich Personal RePEc Archive (MPRA)	Scope: Economics
Social Science Open Access Repository (SSOAR)	Scope: Social Sciences
Stanford Physics Information Retrieval System (SPIRES)	Scope: Physics
WorldBank's Policy Research Working Paper Series (PRWPs)	Scope: Economics

For peer review only
